# Molecular cloning of PRD-like homeobox genes expressed in bovine oocytes and early IVF embryos

**DOI:** 10.1186/s12864-024-10969-w

**Published:** 2024-11-06

**Authors:** Barış Yaşar, Nina Boskovic, Marilin Ivask, Jere Weltner, Eeva-Mari Jouhilahti, Piibe Vill, Tiina Skoog, Ülle Jaakma, Juha Kere, Thomas R. Bürglin, Shintaro Katayama, Tõnis Org, Ants Kurg

**Affiliations:** 1 Department of Biotechnology, Institute of Molecular and Cell Biology, University of Tartu, Tartu, Estonia; 2 Department of Medicine Huddinge, Karolinska Institutet, Huddinge, Sweden; 3 Department of Obstetrics and Gynecology, Helsinki University Hospital, University of Helsinki, Helsinki, Finland; 4 Chair of Animal Breeding and Biotechnology, Institute of Veterinary Medicine and Animal Sciences, Estonian University of Life Sciences, Tartu, Estonia; 5 Department of Pathophysiology, Institute of Biomedicine and Translational Medicine, University of Tartu, Tartu, Estonia; 6 Folkhälsan Research Centre, Helsinki, Finland; 7 Stem Cells and Metabolism and Research Program, University of Helsinki, Helsinki, Finland; 8 Department of Biomedicine, University of Basel, Basel, Switzerland; 9 Centre for Genomics, Evolution and Medicine, Institute of Genomics, University of Tartu, Tartu, Estonia

**Keywords:** Bovine, Reproduction, Preimplantation embryo development, Homeodomain, PRD-like (PRDL) domain

## Abstract

**Background:**

Embryonic genome activation (EGA) is a critical step in early embryonic development, as it marks the transition from relying on maternal factors to the initiation of transcription from embryo’s own genome. The factors associated with EGA are not well understood and need further investigation. PRD-like (PRDL) homeodomain transcription factors (TFs) are considered to play crucial roles in this early event during development but these TFs have evolved differently, even within mammalian lineages. Different numbers of PRDL TFs have been predicted in bovine (*Bos taurus*); however, their divergent evolution requires species-specific confirmation and functional investigations.

**Results:**

In this study, we conducted molecular cloning of mRNAs for the PRDL TFs ARGFX, DUXA, LEUTX, NOBOX, TPRX1, TPRX2, and TPRX3 in bovine oocytes or in vitro fertilized (IVF) preimplantation embryos. Our results confirmed the expression of PRDL TF genes in early bovine development at the cDNA level and uncovered their structures. For each investigated PRDL TF gene, we isolated at least one homeodomain-encoding cDNA fragment, indicative of DNA binding and thus potential role in transcriptional regulation in developing bovine embryos. Additionally, our cDNA cloning approach allowed us to reveal breed-related differences in bovine, as evidenced by the identification of a high number of single nucleotide variants (SNVs) across the PRDL class homeobox genes. Subsequently, we observed the prediction of the 9aa transactivation domain (9aaTAD) motif in the putative protein sequence of TPRX3 leading us to conduct functional analysis of this gene. We demonstrated that the *TPRX3* overexpression in bovine fibroblast induces not only protein-coding genes but also short noncoding RNAs involved in splicing and RNA editing. We supported this finding by identifying a shared set of genes between our and published bovine early embryo development datasets.

**Conclusions:**

Providing full-length cDNA evidence for previously predicted homeobox genes that belong to PRDL class improves the annotation of the bovine genome. Updating the annotation with seven developmentally-important genes will enhance the accuracy of RNAseq analysis with datasets derived from bovine preimplantation embryos. In addition, the absence of *TPRX3* in humans highlights the species-specific and TF-specific regulation of biological processes during early embryo development.

**Supplementary Information:**

The online version contains supplementary material available at 10.1186/s12864-024-10969-w.

## Background

Embryo development begins with the utilization of the maternal sources inside the oocyte following fertilization by the sperm and the subsequent fusion of the two pronuclei. As the newly formed embryo gradually takes over the control of development, it halts its dependency on the maternal mRNAs and proteins, which is achieved through a process called embryonic genome activation (EGA) [1]. EGA refers to the global initiation of transcription from the zygotic genome, which prepares the embryo for further development. PRD-like (PRDL) homeobox genes have been reported to be almost exclusively expressed during this event in humans, especially at 4- and 8-cell stages of early embryo development [2, 3].

PRDL homeobox genes encode transcription factors (TFs) with a DNA-binding homeodomain, but no PAIRED (PRD) domain, and without a serine residue at position 50 of the homeodomain [4]. Based on position 50, PRDL proteins can be divided in 3 groups: R50, Q50 and K50. The R-50 type includes ARGFX (arginine-fifty homeobox), whereas DUXA (double homeobox A) and NOBOX (newborn ovary homeobox) belong to the Q50-type PRDL proteins. The K50-type contains LEUTX (leucine twenty homeobox), TPRX1 (tetra-peptide repeat homeobox 1), TPRX2 (tetra-peptide repeat homeobox 2) and TPRX3 (tetra-peptide repeat homeobox 3). PRDL TF mRNAs are found in high abundance in early embryo development, and they are either absent or at very low levels in somatic cells in adult tissues [5]. Their roles, predominantly exclusive to preimplantation development, have been explored in several studies. Both through modeling and functional experiments LEUTX has been characterized to have DNA binding and transcriptional activation properties in humans [6, 7]. The *DUXA*, being among the first upregulated genes during early embryo development in humans, has also been suggested to compete with, key inducer of EGA, *DUX4* (double homeobox 4) for binding DNA [2, 8]. Knockdown experiments in human embryos revealed that TPRX genes are essential for EGA [9].

Identifying the key factors and species-specific differences involved in EGA is essential for deciphering the intricate process of fertilized egg development in key model organisms. For example, among these factors, inhibition of *c-MYC* and *Plag1* have been demonstrated to result in decreased cleavage/blastocyst formation rates and decreased fetal growth in porcine and mice, respectively [10, 11]. Other potential candidates with significance within the first few days after embryo formation include PRDL TF genes, which are not sufficiently annotated in the current bovine genome, necessitating cDNA cloning of respective genes. To further identify these candidates and demonstrate proof for the expression of EGA-related PRDL homeobox TF genes in bovine preimplantation embryo development, we here cloned cDNA fragments of seven PRDL homeobox genes: *ARGFX*, *DUXA*, *LEUTX*, *NOBOX*, *TPRX1*, *TPRX2* and *TPRX3*. Our cloning approach enabled us to reveal Holstein breed-related single nucleotide variants (SNVs) in our target TF genes. We focused on an unannotated K-50 TF *TPRX3*, because of its homeodomain type and a 9aa transactivation domain (9aaTAD) motif, and tested its transcriptional activation capacity through ectopic expression in bovine fetal fibroblasts. *TPRX3* overexpression induces not only protein-coding genes but also short noncoding RNAs. Our findings aim to benefit the scientific community interested in transcriptomics aspects of bovine early embryo development by providing more accurate annotation.

## Results

### PRDL genes are expressed in early bovine oocytes and IVF embryos

**Fig. 1:**
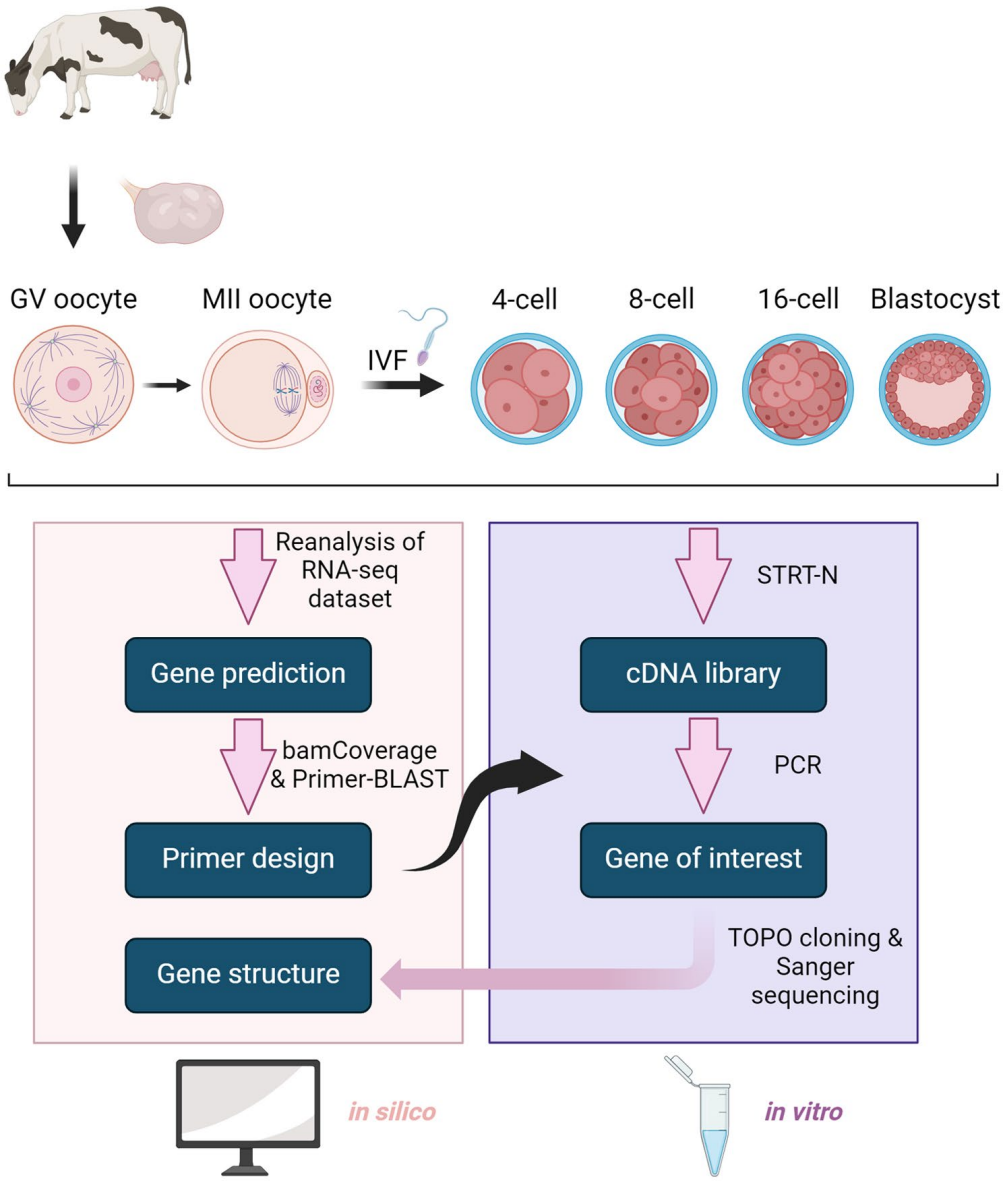
Scheme of the computational and experimental work. Utilization of human PRDL sequences for the gene prediction and cloning strategy are summarized. A dataset (GSE52415) was used to locate PRDL genes in the bovine genome in silico, priming the work for synthesizing cDNA from oocytes and IVF embryos to clone and confirm these genes in vitro. Figure 1 was created in BioRender. Yasar, B. (2024) www.BioRender.com/t22o977

In order to study the role of PRDL genes in bovine preimplantation embryo development, we performed molecular cloning of *ARGFX*, *DUXA*, *LEUTX*, *NOBOX*, *TPRX1*, and *TPRX2* genes, facilitated by complementary *in silico* and in vitro experimental approaches (Fig. 1). Based on the transcriptomic data and homology-based investigation, we pinpointed previously unannotated (*TPRX2*) or misannotated (*ARGFX*, *DUXA*, *LEUTX*, *NOBOX*, and *TPRX1*) PRDL genes in the bovine genome. These genes were either absent or inaccurately annotated in the NCBI Refseq genes and/or Ensemble Gene Predictions (Supplementary Figure S1).

Due to their functional importance as TFs for EGA, we prioritized cDNA cloning of transcripts that encode complete homeodomains [4]. The consensus transcriptome formed by merging transcriptomes from all developmental stages enabled to generate predictions of PRDL TF gene structures (top track, Supplementary Figure S1). The merged transcriptome was used as a reference to determine the expected length of transcripts of interest, enabling us to identify the correct bands on agarose gel after PCR amplification of cDNA. The lengths of the cDNA transcripts that encode complete homeodomains for *ARGFX*, *DUXA*, *LEUTX*, *NOBOX*, *TPRX1*, and *TPRX2* were estimated to be 999 nucleotides (nt), 774 nt, 1809 nt, 1949 nt, 1264 nt, 1207 nt, respectively (Supplementary Figure S2-S8).

**Fig. 2:**
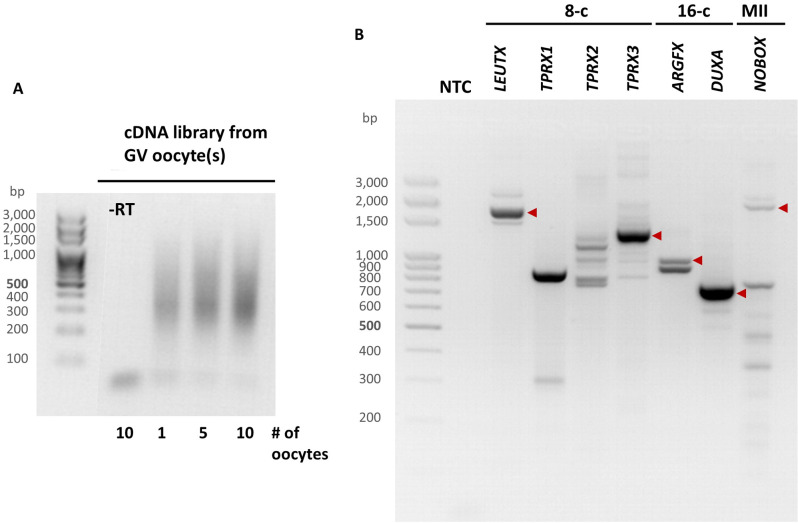
Agarose gel visualizations of cDNA preparation and gene-specific PCR. (**A**) The initial steps of the STRT-N protocol were utilized for cDNA preparation from relevant stages. cDNA libraries from 1, 5, or 10 bovine GV oocytes are shown as an example. (**B**) cDNA preparations derived from respective stages were used for PCR reaction using the primers from Table 1. Amplified homeodomain-encoding PRDL cDNA fragments are marked by arrowheads. NTC: Non-template Control; -RT: minus reverse transcriptase control; 8-c: 8-cell stage; 16-c: 16-cell stage; GV: GV oocyte; MII: MII oocyte. Full-length gels are presented in Supplementary Figure S15

The gene sequence information, obtained through the prediction of PRDL gene structures in bovine, was utilized in the design of cloning primers (Table 1). STRT-N, which is used for the preparation of cDNA with small amounts of RNA, allowed generating cDNAs from bovine material which were later primed for gene-specific PCR [12] (Fig. 2A). Since PRDL transcription factors (TFs) are transiently expressed during early embryonic stages, we selected the developmental stage where each gene is highly expressed based on the published RNAseq data to increase the success of PCR amplification [13]. This meant using cDNA mainly from MII oocyte for *NOBOX*, 8-cell stage for *LEUTX*, *TPRX1* and *TPRX2*, 16-cell stage for *ARGFX* and *DUXA* for PCR amplification (Fig. 2B). Even though we obtained several bands for some genes, the identification of the target band was facilitated by estimated transcript lengths. We were not able to replicate PCR products for genes *TPRX1* and *TPRX2* with 8-cell stage cDNA. Since modifying the PCR conditions did not lead to an improvement of the results, we opted to utilize cDNA from the 16-cell stage, during which the expression levels of *TPRX1* and *TPRX2* are at levels to enable PCR (Supplementary Figure S9A, S9B). In addition to the amplified fragment from the 16-cell stage, the highly prominent band from the 8-cell stage was also selected for *TPRX1* molecular cloning despite its migration at a different length than expected (Fig. 2B). For *DUXA* and *LEUTX*, we designed an additional pair of cloning primers to detect isoforms that have exclusive sequences at their 5’- or 3’-end (Table 1). Overall, the PCR results show that fragments, which are amplified to contain homeodomain and putative transcription start site (TSS), are visible at the expected lengths on agarose gel, confirming the expression of studied PRDL homeobox genes in oocytes and early embryos (Fig. 2B).

**Table 1:** Cloning of PRDL homeobox genes

Gene acronym	Full name	Locus (bosTau9)	Strand	Forward/Reverse Primer	Developmental stage
*ARGFX*	arginine-fifty homeobox	chr1:66070409–66087657	+	AGGGTATTTCACCATAAGGACACA/GTGCACTGTAATGGAGGGGT	16-cell
*DUXA*	double homeobox A	chr18:64398319–64406694	-	CAAGACGGTCCTTCAGACAAGA/TGGATTTGGTCACTAGGGCTG	16-cell
				TTATTGGTCTCTCTCTCCCCAG/TCACTAGGGCTGCATGGTTC	
*LEUTX*	leucine twenty homeobox	chr18:49330772–49336014	+	GCAACACCTGGTCGAATCACT/GGGTTTTCGCACCATACGTG	8-cell
				TCAGGCAACACCTGGTCGAA/AGCCTGAGAATACGGAGTGG	
*NOBOX*	newborn ovary homeobox	chr4:107667138–107674196	-	GTGCGGGAGTCCAGGC/CTGTGGAGCAAGGAGAGCAA	MII oocyte
*TPRX1*	tetra-peptide repeat homeobox 1	chr18:54684552–54686759	-	CAGCAGGAGGATGCAAGAACC/TCAGCGTATCATCAGTGTGCCA	8-/16-cell
*TPRX2*	tetra-peptide repeat homeobox 2	chr18:54725806–54727922	+	AGCATCAAACTCCGACAGCA/ACAGGTAAGGATAGAGACAAGCA	16-cell
*TPRX3*	tetra-peptide repeat homeobox 3	chr18:62775020–62777476	-	GCAGCAGTGAGGAACCAAGA/AGAGGCACTGAAATAGACAGGAA	8-cell

Our transcript assembly analysis did not generate a complete *DPRX* transcript, which is in line with the earlier report suggesting *DPRX* to be a pseudogene in bovine [14]. Another study, which refers to a TPRX duplicate as *TPRX3* in bovine, prompted us to examine this gene in bovine genome [15]. As a result of the same workflow applied to the rest of the PRDL genes, we predicted the gene structure of *TPRX3* with its transcript length being 1403 nt (Supplementary Figure S8). Putative protein sequence of human TPRX1 from our earlier study helped identify the locus of *TPRX3* through tblastn (Materials and methods) [5]. Like for other genes, the primers were designed to amplify predicted TSS and conserved homeodomain. The cloning result demonstrated the presence of *TPRX3* transcripts in bovine preimplantation embryos (Fig. 2B).

**Fig. 3:**
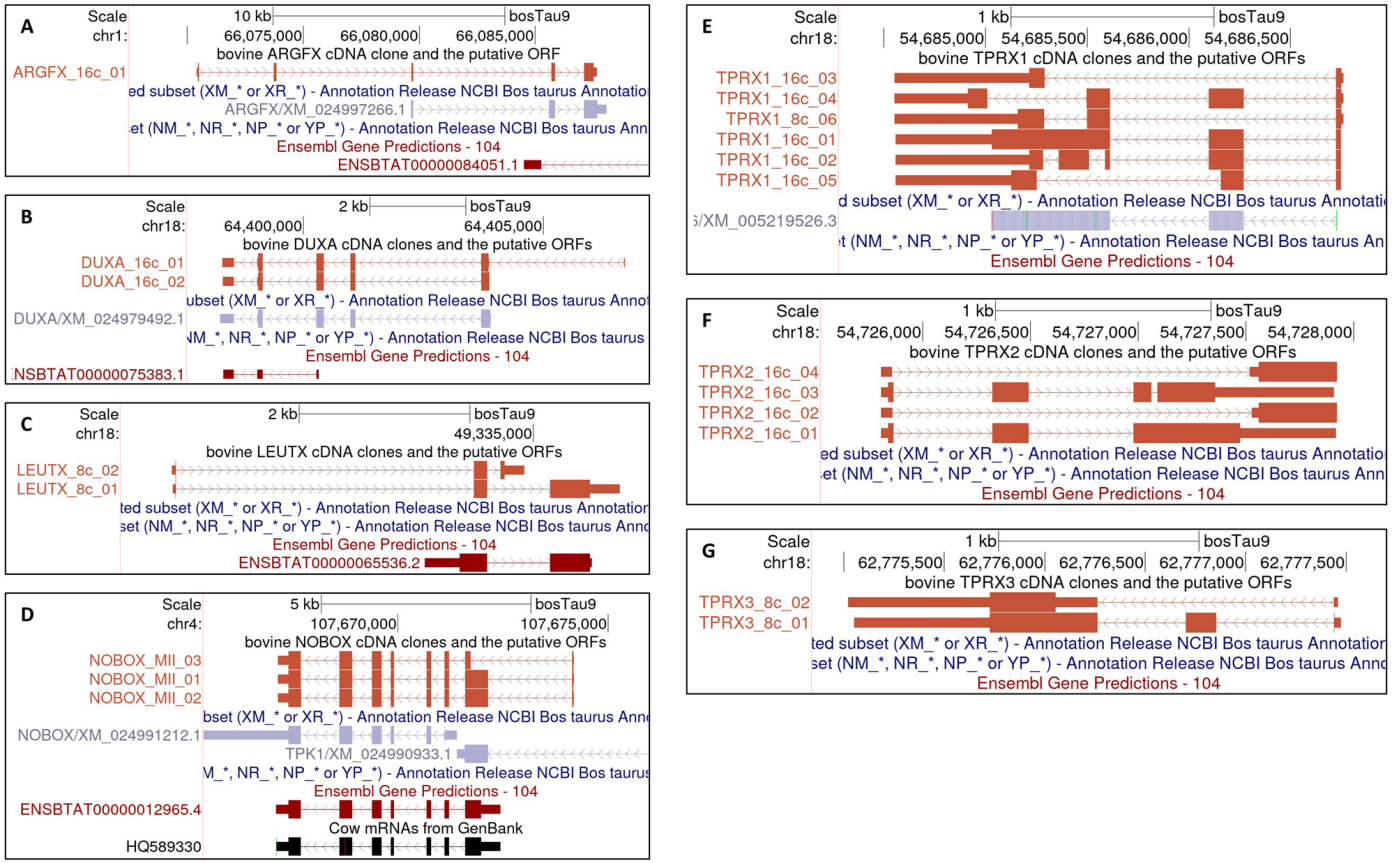
Gene structures of PRDL genes deduced from cDNA cloning. Each unique transcript for all analyzed PRDL genes, namely (**A**) *ARGFX*, (**B**) *DUXA*, (**C**) *LEUTX*, (**D**) *NOBOX*, (**E**) *TPRX1*, (**F**) *TPRX2*, (**G**) *TPRX3*, is visualized on UCSC Genome Browser in a rust brown color. Gene names are followed by the origin of developmental stage, and numbers ranging from 01 to 06 are assigned depending on the number of unique transcripts. For comparison, current bosTau9 gene annotation (NCBI RefSeq genes; light purple, Ensembl Gene Predictions; maroon, etc.) is included in the lower tracks. Previously cloned *NOBOX* (HQ589330) is also shown in black for comparison

Having amplified seven PRDL gene cDNA fragments from bovine oocytes and/or early embryos, we performed molecular cloning followed by Sanger sequencing in order to determine their sequences. The alignment of cloned cDNA sequences to the latest genome assembly of bovine, bosTau9, revealed exon-intron structures of PRD-like homeobox genes in *Bos taurus* (Fig. 3, Supplementary Text 1). Our findings indicate that *ARGFX* and *NOBOX* are located on chromosomes 1 and 4, respectively, while *DUXA*, *LEUTX*, *TPRX1*, *TPRX2* and *TPRX3* are located on chromosome 18. This is cDNA sequencing-based confirmation of an earlier report that predicted the genomic positions and structures of PRDL TFs [15]. Putative protein sequences of *LEUTX*, *NOBOX*, *TPRX1*, *TPRX2* and *TPRX3* were each found to contain one homeodomain, whereas two homeodomains were detected in *DUXA*’s putative protein sequence (Supplementary Figure S10). *DUXA*, *NOBOX*, *TPRX1* and *TPRX3* are transcribed from the negative (-) strand while *ARGFX*, *LEUTX* and *TPRX2* are transcribed from the positive (+) strand. In addition, with the exception of *ARGFX*, we were able to identify more than one isoform for all PRDL genes.

### cDNA cloning revealed and confirmed 5’-end novel exons in PRDL genes

We identified two novel exons at the 5’-end of *ARGFX*, which are lacking in current RefSeq annotation (Fig. 3A). These two exons are part of the putative open reading frame (ORF) enabling the prediction of a more reliable primary amino acid sequence with a complete homeodomain. ARGFX protein sequence derived from our cDNA evidence validates *Bos taurus* ARGFX protein sequence reported earlier in the multiple sequence alignment, with only minor differences observed between the sequences that may be attributed to breed-specific SNVs and indels [15].

A member of the intron-containing *DUX* gene family, *DUXA*, was found to have two isoforms, one of which includes a novel exon located upstream of the currently predicted first exon (Fig. 3B). Both variants retain their putative DNA binding ability through their homeodomains. Our cDNA aligned with the present proposed model for *DUXA*, but it does not cover the sequence encoding the potential first methionine. Instead, it aligned with an alternative downstream methionine-encoding codon as a start site for translation of *DUXA* (Supplementary Figure S11A). Either ORF will result in a protein with double homeodomains, which is a unique feature of DUX family among other homeodomain-containing transcription factors that may contain another DNA-binding domain, but not of the same type [8]. Based on NCBI RefSeq predicted gene subset, the neighboring anchor genes upstream (*USP29*, *ZIM3*) and downstream (*AURKC*, *ZNF543*) of *DUXA* confirmed the syntenic relationship between human and bovine (Supplementary Figure S11B).

Querying *LEUTX* in UCSC Genome Browser takes users to an irrelevant position in the bosTau9 genome on chromosome 29. Furthermore, neither predicted nor curated gene models are available for *LEUTX* in the NCBI Refseq. According to our cDNA cloning results, *LEUTX* has two transcript variants, both of which provide a novel 5’-end exon where the ORF starts, compared to the current Ensembl Gene Predictions version 104 (Fig. 3C). Apart from the breed-related differences, our cDNA evidence confirmed almost the entire protein sequence of bovine LEUTX that was proposed in a study investigating LEUTX across different species from an evolutionary aspect [6, 15].

The cDNA cloning for *NOBOX* was previously reported to reveal an additional exon compared to the RefSeq gene prediction, XM_024991212.1 [16]. However, our cDNA cloning discovered one more exon upstream of the first exon of the clone HQ589330, extending the possible ORF (Fig. 3D).

*TPRX1* was found to undergo alternative splicing to a very high extent, as demonstrated by our cDNA cloning, which identified six different transcript variants (Fig. 3E). Two of these *TPRX1* transcripts do not code for a homeodomain. For *TPRX2*, we detected four transcript variants, which were completely missing in all current bovine gene annotations (Fig. 3F). *TPRX3*, which is so far known to be exclusive to bovine and porcine, displayed two isoforms (Fig. 3G) [14]. *TPRX1*, *TPRX2* and *TPRX3* all had at least one transcript variant that included a homeodomain coding sequence (Supplementary Figure S6-8).

Using our cDNA cloning-based annotations of seven PRDL TF genes and an early bovine embryo dataset from another study that utilized spike-in RNAs for highly accurate relative quantification, we plotted the dynamic changes in the expression of these genes. The results showed that *ARGFX*, *DUXA*, *LEUTX*, *TPRX1*, *TPRX2* and *TPRX3* reached their highest expression levels at the 16-cell stage suggesting their role in bovine EGA [17] (Supplementary Figure S12).

### EGA genes contain high number SNVs in Estonian Holstein breed of *Bos taurus*

**Fig. 4:**
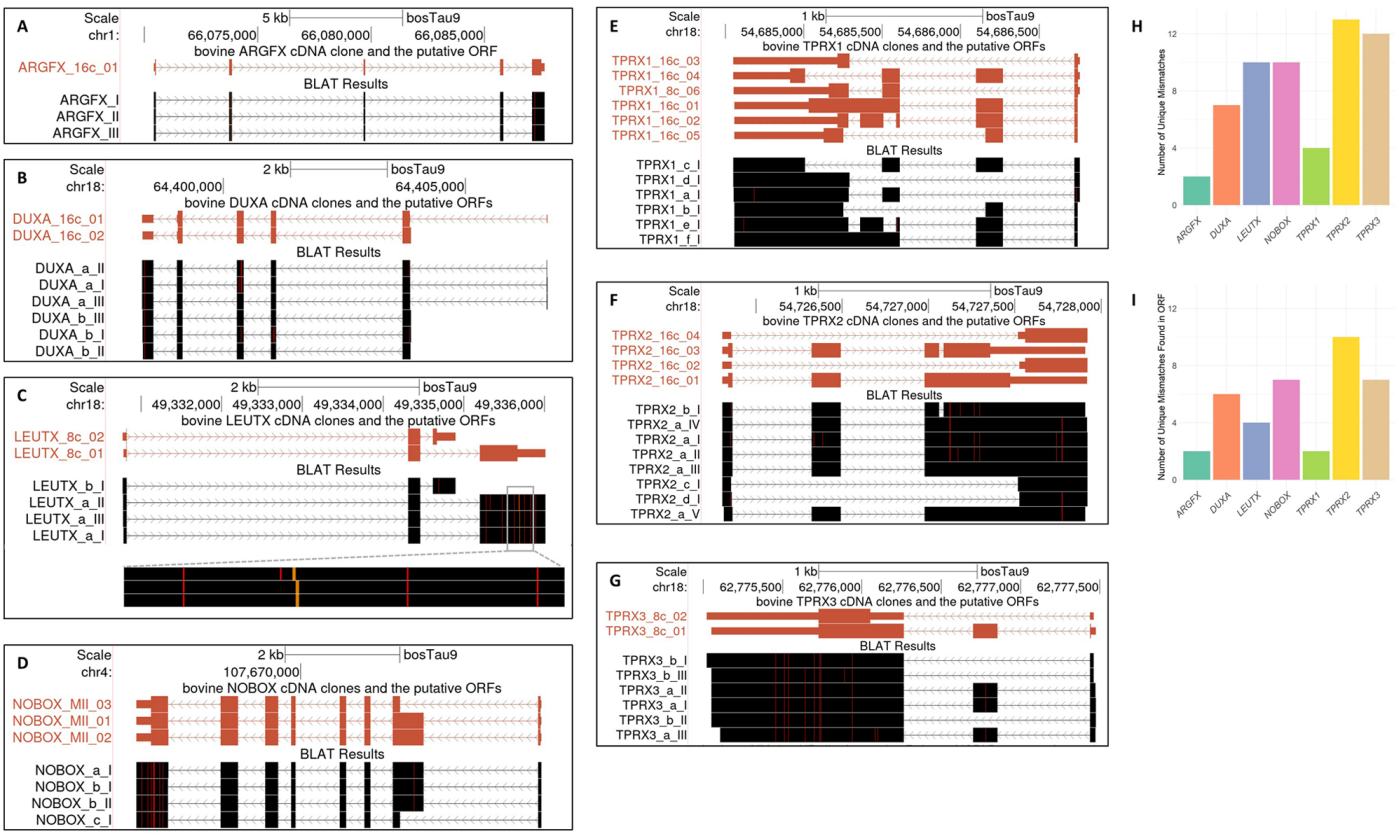
Mismatches in the alignment of PRDL cDNA clones from Estonian Holstein oocytes or IVF embryos. (**A**-**G**) Each cDNA clone is labeled with a letter (a, b, c, d, e, f, g) corresponding to its unique structure, followed by the replicate number (I, II, III) of that specific clone. Red vertical lines indicate a mismatch at that specific location, while orange vertical line represents an insertion. (**H**) The distribution of unique 58 mismatches across PRDL genes investigated. (**I**) The distribution of mismatches found in ORF for the given gene. Supplementary Text 1 and 2 can be used for custom visualization on the UCSC Genome Browser

The alignment of cDNA clones, derived from Estonian Holstein breed, against the reference bovine genome assembly revealed several mismatches in the gene body as well as in the 5’- and 3’- UTRs (Fig. 4A-G). As the reference bosTau9 genome originates from the Hereford breed, we conducted further analysis to confirm these observed mismatches as SNVs, potentially representing Estonian Holstein-specific SNVs. For this purpose, we reanalyzed whole-genome sequencing (WGS) data obtained from Estonian Holstein bovine and Holstein bull (PRJNA183919, PRJNA184837) [18]. In addition, we reanalyzed *Bos taurus* Angler breed WGS data from the Swiss Comparative Bovine Resequencing (SCBR) project (ERX1815535).

Among the isolated cDNA fragments, we identified a total of 58 unique SNVs or indels (Supplementary Table S1-2, Supplementary Text 2). Thirty-seven of them were found in at least one of the genomic read alignments of Holstein origin, while 21 of them were not found in the re-analyzed three WGS datasets. Out of 21 SNVs or indels, 18 were in the ORF with three being in 3’-UTR. Interestingly, among the 18 SNVs found in ORF, almost half of them turned out to be synonymous. Each PRDL class homeobox gene we cloned seemed to contain an SNV and/or an indel in line with their divergent nature [19]. The distribution of total 58 unique SNVs across seven PRDL class genes was similar for *LEUTX*, *NOBOX*, *TPRX2*, and *TPRX3*, with a slightly lower number in *DUXA* (Fig. 4H). *ARGFX* and *TPRX1* had a relatively lower number of SNVs. The number of unique SNVs that occurred in the ORF was at least two, and above four for four of the seven genes analyzed (Fig. 4I).

All *ARGFX* cDNA clones exhibited one non-synonymous SNV outside the homeobox in the ORF, along with a 13 bp indel affecting the homeobox. Aligning our wild-type *ARGFX* sequence from Holstein to the reference genome derived from a Hereford origin, which was earlier reported to have a minor allele frequency of 0.667 for the given 13 bp indel [19] appeared as an insertion in our visualization (Fig. 4A). In fact, this was the 13 bp deletion in the original reference bosTau9 genome, which is also found in other bovine breeds [19].

The *DUXA* cDNA contained six SNVs (one synonymous and five non-synonymous) in the ORF and one SNV in the 3’UTR. Among the cDNA clones, only one contained an SNV in the second homeobox of *DUXA*, leading to the truncation of the encoded homeodomain (Supplementary Figure S13). The SNVs in *LEUTX* were shared among all clones, except for one exclusive synonymous SNV that was identified in a single *LEUTX* cDNA clone. There were four SNVs located outside the homeobox in the ORF, along with a total of five SNVs and a 3 bp indel in the 3’UTR. The *NOBOX* cDNA clones exhibited 10 unique SNVs, with seven found in the ORF (three synonymous and four non-synonymous) and three in the 3’UTR. Out of the seven SNVs found in the ORF, six were located in the coding region for the C-terminus, while none were found in the region encoding the homeodomain. The *TPRX1* cDNA revealed two synonymous SNVs in the ORF, with one located in the homeobox not altering the amino acid sequence. Furthermore, two SNVs were identified in the 3’UTR. The *TPRX2* cDNA contained 10 SNVs in the ORF, consisting of four synonymous and six non-synonymous SNVs. None of these SNVs were found in the homeobox. Additionally, there were three SNVs identified in the 3’UTR. The *TPRX3* cDNA clones exhibited 12 SNVs, with seven found in the ORF (three synonymous and four non-synonymous) and five in the 3’UTR. Overall, a closer inspection of SNVs showed that nucleotide changes occurring in the coding region did not disturb the amino acid sequence encoding the homeodomain of the investigated PRDL genes, except for one *DUXA* cDNA clone.

### Comparison of PRDL proteins

**Fig. 5:**
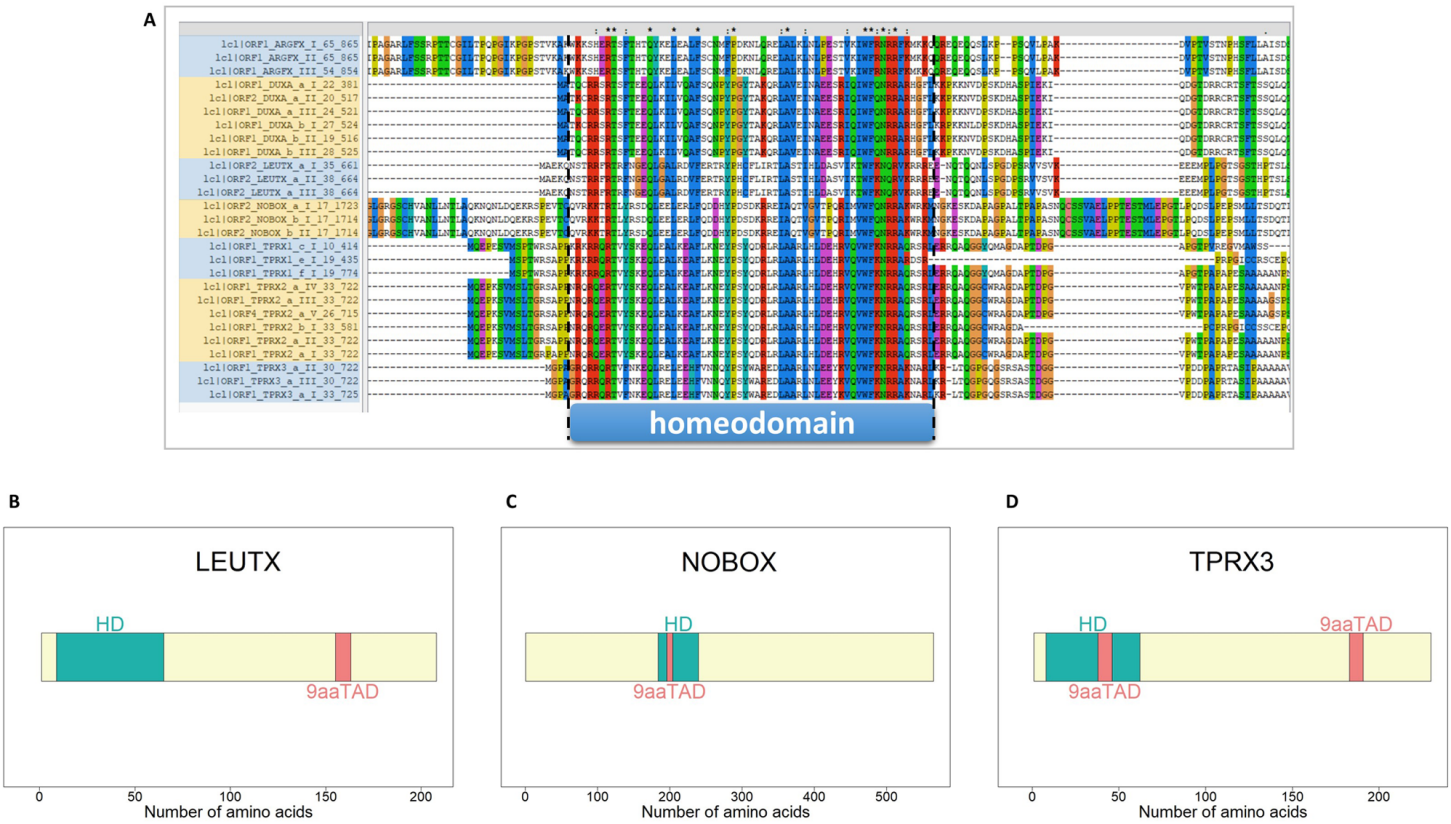
Primary structures of PRDL proteins. (**A**) Alignment of amino acid sequences corresponding to homeodomains in PRDL TFs. cDNA clones encoding complete putative homeodomains from *ARGFX*, *DUXA*, *LEUTX*, *NOBOX*, *TPRX1*, *TPRX2*, *TPRX3* were selected to visualize conserved residues. SNV-related differences among the same proteins from different clones are also shown. First homeodomain of DUXA is shown. similar residue; *same residue. (**B**-**D**) Domain structures of bovine LEUTX, NOBOX, and TPRX3 proteins. Transcripts that encode for the longest ORF and homeodomain were chosen to visualize proteins. LEUTX_a_I, NOBOX_b_II, and TPRX3_a_I cDNA clones were used for ORF and domain predictions in the figure. Based on the putative protein’s primary FASTA sequence homeodomain and nine amino acid transactivation domain (9aaTAD) were marked in light pale green and light coral, respectively

We searched every cDNA clone for the presence of a homeodomain using hmmscan and performed multiple sequence alignment in order to visualize their intact primary structure independent of the majority of the SNVs we reported earlier. Known conserved residues forming the homeodomain for this protein class such as the tryptophan (W) at position 48, the asparagine (N) at position 51, and the arginine (R) at position 53 were verified to be present in all of the putative PRDL protein sequences (Fig. 5A). In addition, the residue 50 confirms our cloned *ARGFX* to be R-type, *DUXA* and *NOBOX* to be Q-type, and *LEUTX* and *TPRX* s to be K-type PRDL TF genes. According to SMART illustration (Supplementary Figure S10), LEUTX, TPRX1, TPRX2, and TPRX3 have the homeodomain on their N-terminus whereas for ARGFX and NOBOX it is positioned relatively in the middle. DUXA has the same domains at both the N- and C-termini (Supplementary Figure S10). With the exception of one DUXA cDNA clone, no SNVs were found in the regions encoding the homeodomain. The visualization of primary protein structures highlights that all seven PRDL genes are likely to have a DNA binding function in early bovine development.

Further evidence for a TF to have a potential functional significance is the presence of the 9aa transactivation domain (9aaTAD) in its primary sequence. The 9aaTAD motif is a nine-amino-acid-long sequence within transactivation domains (TADs) experimentally found to be involved in autonomous activation of transcription [20]. Human ARGFX as well as LEUTX contains this motif in their C-terminal regions [6]. Deletion of the 9aaTAD from LEUTX was reported to cause the loss of the stable interaction with its binding partners, histone acetyltransferase EP300 and cofactor CBP [21]. For this reason, we used 9aaTAD Prediction tool and found out that among the analyzed seven PRDL TF proteins, only three of them contained putative 9aaTADs (Fig. 5B-D). Similar to humans, bovine LEUTX is predicted to have a 9aaTAD towards the carboxy terminus [6]. Interestingly, only one member of TPRXs, TPRX3 was predicted to have not just one but two 9aaTADs. One of the 9aaTADs in TPRX3 was in the homeodomain, similar to NOBOX, whereas the other one was, similar to LEUTX, at the C-terminus. The significance of putative 9aaTADs in the homeodomain is unclear, as it may not form the expected structure. Moreover, none of the observed SNVs were found within the DNA region coding for the residues forming the 9aaTAD, supporting the potential conserved role for this domain in development.

### Overexpression of *TPRX3* to test its effect on transcription

**Fig. 6:**
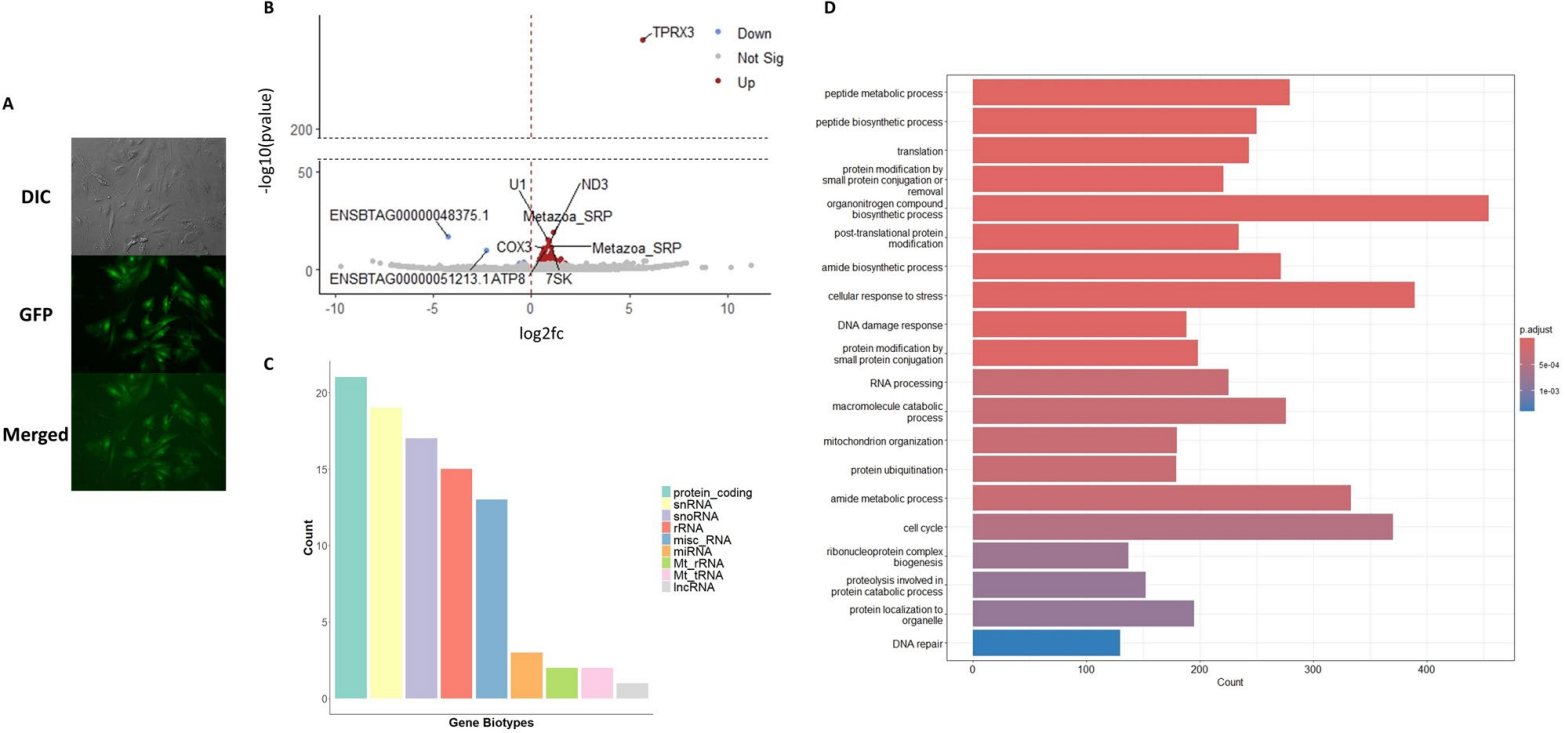
Overexpression of *TPRX3* in bovine fetal fibroblasts (BFFs). (**A**) Images were taken 24 h following the induction with doxycycline for 24 h. (**B**) The volcano plot illustrates differentially expressed genes by the experiment. Points are genes and the significant ones (adjusted *P* < 0.05) were colored by red (as up-regulated) or blue (as down-regulated). The top 10 significant genes were labeled. Ensembl gene ID was used to label for genes that did not have an assigned gene name. (**C**) The bar plot shows distribution of significantly differentially expressed genes based on their gene biotypes. (**D**) Gene ontology (GO) enrichment analysis. DIC: Differential Inference Contrast; GFP: Green Fluorescent Protein

The identification of 9aaTADs in the putative TPRX3 protein raised the possibility that it has transcriptional activation capacity, which might contribute significantly to bovine EGA. For this reason, we set out to test if *TPRX3* can modulate gene expression using a doxycycline-inducible overexpression system in bovine fetal fibroblast cells (BFF). We cloned the longer variant of the *TPRX3* cDNA fragment into the respective expression vector, where *TPRX3* is connected to downstream GFP reporter through the IRES sequence. Following electroporation and antibiotic selection, we treated the cells with doxycycline for 24 h, which implied the successful integration and expression of *TPRX3* (Fig. 6A).

Two replicates qualified for the downstream RNAseq analysis. We obtained minimum of 44,500,066 and maximum of 59,887,867 reads after trimming and filtering. The alignment rate ranged from 80.41 to 89.76% (Supplementary Table S3; Supplementary Figure S14). Principal Component Analysis demonstrated a separation of replicates along PC1 (74%) and a separation of the treatment along PC2 (21%), which was also reflected in the hierarchical clustering (Supplementary Figure S14A and B). Having data that provided good fit for the DESeq2 (Supplementary Figure S14B), we identified 87 significantly upregulated and six significantly downregulated genes (adjusted *P* < 0.05) (Supplementary Data 3), suggesting that *TPRX3* mostly acts as a transcriptional activator. The overexpression of *TPRX3* in BFF cells was confirmed in the volcano plot where it stood out as the gene with the highest log fold change and the highest level of significance (adjusted *P* < 0.05) (Fig. 6B). Interestingly, even though protein coding genes make up the highest proportion of the DE genes, non-coding genes such as small nuclear RNA (snRNA), small nucleolar RNA (snoRNA), ribosomal RNA (rRNA) were also activated as a result of *TPRX3* ectopic expression (Fig. 6C). In line with this observation, top three enriched gene ontology (GO) terms included peptide metabolic process, peptide biosynthetic process, and translation (Fig. 6D).

### Comparison of *TPRX3* overexpression dataset with datasets derived from bovine early embryos

**Fig. 7:**
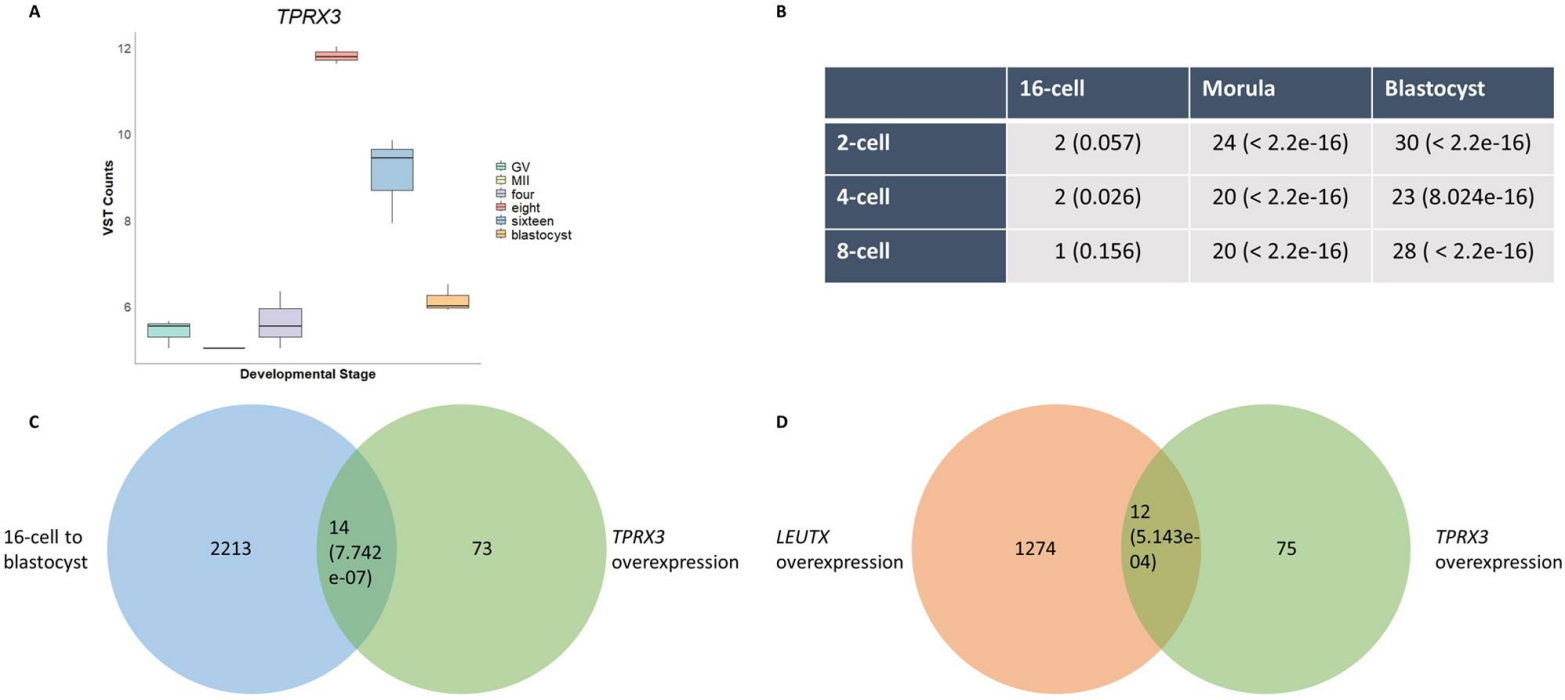
Comparison of *TPRX3* overexpression dataset with other datasets. (**A**) The boxplot shows the expression levels of *TPRX3* based on variance stabilizing transformation (VST) counts derived from Graf et al. [13] (**B**) The table illustrates a comparative analysis of significantly upregulated genes between the dataset by Paulson et al. [22] and *TPRX3*-overexpressed bovine fetal fibroblast cells, conducted in a stage-specific manner. Stages of embryo development compared can be seen on the left side and top of the table. The table provides the number of shared genes between the datasets and the adjusted P-values enclosed in the brackets. (**C-D**) Venn diagrams show the number of significantly upregulated genes in this study in comparison to that of (**C**) Graf et al. [13] and (**D**) Lewin et al. [19]. Fisher’s Exact Test was performed for calculating P-values, shown in parenthesis

During the preimplantation period, according to the dataset derived from Graf et al., *TPRX3* expression reached its peak at the 8-cell stage (Fig. 7A). In order to identify whether *TPRX3* overexpression-mediated upregulated genes in bovine fibroblasts might be relevant in early embryogenesis, we compared our dataset with another dataset derived from bovine IVF embryos whose library preparation did not exclude non-poly(A) tailed RNAs [22]. The comparison of upregulated DE genes in both datasets revealed significant overlaps especially when morula and blastocyst stages were considered with respect to earlier stages (Supplementary Table S4). Twenty-four and 30 significantly upregulated DE genes in the 2-cell-to-morula and 2-cell-to-blastocyst transition detected by small RNA sequencing were also significantly upregulated (adjusted *P* < 0.05) in our dataset, respectively (Fig. 7B). Significantly upregulated DE genes in the 16-cell-to-blastocyst transition derived from another dataset [13] had 14 common genes with the *TPRX3*-mediated significantly upregulated (adjusted *P* < 0.05) genes (Fig. 7C, Supplementary Table S5). Considering the key residues aiding DNA binding shared between TPRX3 and LEUTX, specifically, lysine 57 [7] and arginines found in the homeodomain at the position 2/3 and 5 in the homeodomain [6], we compared data on *LEUTX* ectopic expression in fibroblasts from an earlier study [19] with our dataset. This comparison revealed 12 overlapping significantly upregulated genes between two datasets (Fig. 7D). As a result, comparing the overexpression approach we employed with datasets generated from developing preimplantation embryos suggest that observed results are not only secondary effects of ectopic expression but also indicate the biological relevance of *TPRX3* in the bovine embryo development.

## Discussion

Molecular cloning is considered a gold standard method for verifying gene structures, identifying alternative transcript variants, discovering novel TSS, and providing evidence for updating the annotation of the genome to RefSeq. Although transcript assembly tools aid in predicting novel transcripts, when combined with short-read sequencing-based methods, they do not ensure accurate representation of gene structures. In our study, we used bovine early oocytes and IVF embryos to experimentally validate the expression of PRDL TF genes, which have been shown to have important roles in early human embryo development in several studies. More specifically, *ARGFX*, *LEUTX* and *TPRX* s have been shown to have transcription activation role, while *DUXA* is hypothesized as a competitive inhibitor of *DUX4* given their shared chromatin occupancy [2, 5–8].

Due to the similarities between bovine and human in terms of reproduction, and its suggested significance as a better model organism for preimplantation embryo development, we investigated the PRDL TF genes *ARGFX*, *DUXA*, *LEUTX*, *NOBOX*, *TPRX1*, *TPRX2*, and *TPRX3* in bovine [23–28]. Our results not only provide an update for genome annotation, which is likely to change the interpretation of bovine preimplantation embryo-derived dataset analyses, but also highlight the involvement of similar TFs between bovine and human, possibly during EGA.

The method of choice for cDNA preparation, STRT-N, enabled us to get cDNA evidence and detect transcript far 5’-ends (TFEs) [2, 12]. Defining the first exon of a gene based on experimental evidence is an essential step for accurate gene annotation and analyses such as motif enrichment, which rely on the annotated TSS.

We identified novel 5’-end exons for *ARGFX*, *DUXA*, *LEUTX*, *NOBOX* and several transcript variants for *TPRX* s. Similarly, several PRDL genes have previously been cloned using human embryos [5]. Moreover, our prior related study shed light on the role of *LEUTX* in human EGA and highlighted the discovery of a novel first exon that encodes the first methionine in the ORF [7]. This agrees with our bovine *LEUTX* as well as *TPRX3* gene model based on molecular gene cloning. Earlier analysis of the bovine *DUXA* gene demonstrated a model with four exons and the absence of synteny information [29]. Our results on *DUXA* suggest shared synteny between human and bovine. In addition, our confirmed gene models for *ARGFX*, *LEUTX*, *TPRX1*, *TPRX2* and *TPRX3* seem to be in line with the gene structures predicted earlier [15]. The presence of shared PRDL TF genes suggests a close relationship between human and bovine, as mice are believed to have lost *Argfx*, *Leutx*, and *Tprx1* from their genome, indicating the involvement of different players or mechanisms in EGA [30].

Certain residues in human LEUTX and ARGFX homeodomains that are under positive selection have been identified as determinants of DNA binding specificity, namely residue R7 and S43, respectively [15]. The bovine counterparts of these residues are identical suggesting their functions in DNA binding. LEUTX homeodomain residues suggested to recognize minor groove of the DNA in human, R2,3 and 5, are also found in bovine LEUTX [6]. Another residue that was found to be under positive selection is Q1 in the homeodomain of human TPRX1 and TPRX2 [15]. In bovine, this residue is conserved in TPRX2 and TPRX3 but not in TPRX1 implying their more pronounced roles. Unlike human ARGFX, bovine ARGFX is predicted to lack the 9aaTAD motif [6].

K-50 type homeodomain PRDL TFs have a DNA binding site that allows them to bind to a consensus motif TAATCC [31, 32]. In humans, the transcription activation ability of LEUTX, as assessed by a luciferase reporter driven by EGA-enriched Alu-motif (EEA motif), was thought to be mediated by the TAATCC sequence within the EEA [7]. In addition, a later study confirmed the activation of genes targeted by TPRX1, with low activation of genes targeted by TPRX2, in addition to those targeted by LEUTX, through a shorter core sequence centered around TAATCC [6]. A recent study compared bovine SCNT and IVF embryos and found that the DUX4/DUXC binding motif was enriched in chromatin regions that could not be opened in the former according to scATAC-seq results [33]. The predicted DUX4/DUXC binding motif contains the subset sequence TAATCC [33]. This subset sequence could be a potential binding site for the PRDL TFs, including TPRX3. Through these binding sites, the bovine PRDL TFs could exert their early developmental role. Another study hypothesizes that LEUTX and DPRX act as a transcription activator and repressor in human 8-cell stage embryos, respectively [7]. However, the absence of *DPRX* expression in bovines leads us to speculate that there may be another gene that assumes the repressor role of DPRX.

Furthermore, our data revealed the presence of *TPRX3*, which has been predicted to be exclusively present in bovine and porcine species [14]. This prompted us to evaluate the role of TPRX3 in bovine early development. The fact that *TPRX3* mRNA encodes a complete homeodomain and residues constituting the 9aaTAD makes it an interesting candidate for a more specialized role in the regulation of gene expression during embryonic development.

Unlike studies that traditionally used poly(A)-targeted methods for cDNA library preparation to study PRDL TFs [5, 7], we utilized random primers in RNAseq preparation, allowing the detection of deadenylated maternal transcripts and enabling us to uncover the capacity of *TPRX3* to activate various noncoding RNA. Combining gene and domain structure predictions with a functional experiment and its subsequent comparative analysis with related datasets, this study suggests previously undiscovered roles for *TPRX3* in bovine embryo development. However, low replicate number of RNAseq experiment presents a major weakness of our study, which is likely the reason of identifying low number of DEGs. Additionally, our experimental design did not include a control for the effect of doxycycline and, mitochondrial-encoded genes identified as DEGs might be a secondary effect of doxycycline [34] (Supplementary Data S3). Another limitation of this study is the cell model used for the overexpression experiment. Although it might be informative for understanding indirect and direct transcriptional effect, a differentiated cell has a distinct chromatin architecture that might expose completely different accessible genomic regions for TPRX3 to bind as compared to early period when this gene is mostly active. With their possible wider implementation and easier access in future, bovine embryonic stem cells could provide more insights into TPRX3 through functional experiments when combined with a higher number of replicates.

Even though our study also shows interesting similarities between human and bovine early embryos in terms of PRDL homeobox genes, the absence of *TPRX3* in humans necessitates the acknowledgement of differences, as well. Therefore, it is also important to underscore the significance of studying EGA in a species- and TF-specific manner.

## Conclusions

In summary, this study confirms the expression of seven PRDL homeobox transcription factor transcripts in bovine early embryo development, reveals the evolutionary pressure on domesticated animals judged by the SNVs discovered in developmental genes, and uncovers the transcriptional activation capacity of TPRX3, which points to its potential role during initial days of bovine embryo development.

## Materials and methods

### Embryo culture

The oocytes and embryos of Holstein breed bovine origin were provided, through a collaboration, by the Estonian University of Life Sciences (Tartu, Estonia). Oocyte collection, in vitro maturation, in vitro fertilization, and in vitro cultivation were conducted as described earlier [35]. Estonian Holstein origin Cardell (27794) or Ziard (27481) semen was used for in vitro fertilization.

### Preparation of cDNA for bovine oocytes and embryos

Three to five oocytes from MII-oocyte stage or three to five embryos from the 8-cell, 16-cell stages were collected in two separate batches. Each batch was pooled based on the stage, the collected sample were lysed in STRT lysis buffer. The lysis buffer contained 2.5 mM dNTP mix (ThermoFisher Scientific), 1 U/µl RiboLock RNase Inhibitor (ThermoFisher Scientific), 5 mM Pierce™ DTT (ThermoFisher Scientific), 10 mM Tris pH 8.0, 0.1% Triton™ X-100 (Sigma-Aldrich), and 0.525 µM STRT-T30 V-LNA (TTAAGCAGTGGTATCAACGCAGAGTCGACT_29_ + V). The cDNA preparations were done according to the STRT-N protocol with minor modifications [12]. Briefly, frozen samples in STRT buffer without pre-thawing were incubated at 80 °C for 2 min to denature RNA and immediately placed on ice prior to first strand synthesis. Reverse transcription reaction was composed of 952 mM Betaine solution, 1x SuperScript™ IV RT buffer (ThermoFisher Scientific), 4.76 U/µl SuperScript™ IV Reverse Transcriptase (ThermoFisher Scientific), 4.8 mM DTT, 0.95 µM template switching oligo (TSO; Biotin-ACGACGCTCTTCCGATCTN_8_rGrGrG), 0.95 U/µl RiboLock RNase Inhibitor (ThermoFisher Scientific). The following program was used for reverse transcription: 42 °C for 60 min; 10 cycles of 50 °C for 2 min, 42 °C for 2 min; 85 °C for 5 min for inactivation. Following reaction cleanup with Dynabeads™ MyOne™ Carboxylic Acid (ThermoFisher Scientific) coupled with capture buffer (25% PEG-600, 2.25 M KCl), PCR amplification was performed. The PCR reaction was assembled with the components of 1x Phusion GC buffer (ThermoFisher Scientific), 3% DMSO, 0.197 mM dNTP mix (ThermoFisher Scientific), 0.04 U/µl Phusion DNA Polymerase (ThermoFisher Scientific), 1 µM universal primer (AAGCAGTGGTATCAACGCAGAGT), and 1 µM primer 1 (Biotin-ACACGACGCTCTTCCGATCT). The following program was used for cDNA amplification: 98 °C for 1 min; 98 °C for 10 s; 2 cycles of 55 °C for 30 s and 72 °C for 5 min; 98 °C for 10 s; 23 cycles of 62 °C for 30 s and 72 °C for 5 min. Next, PCR products were treated with exonuclease I (Sigma-Aldrich) and Shrimp Alkaline Phosphate (New England Biolabs) to remove remaining primers and dNTPs.

### Gene prediction

A dataset consisting of bovine oocytes and preimplantation embryos was used for reanalysis [13]. The reanalysis was performed on the Galaxy platform [36]. The downloaded FASTQ files served as input for mapping against the bosTau9 reference genome using HISAT2 [37]. The specific option “Report alignments tailored for transcript assemblers including StringTie” was selected. The resulting BAM files from each replicate per developmental stage were then inputted into samtools merge [38]. For transcript assembly, merged BAM files and reference annotation file obtained from UCSC Table Browser were used as inputs for StringTie [39, 40]. The Apr. 2018 (ARS-UCD1.2/bosTau9) assembly, Genes and Gene Predictions group, NCBI RefSeq track, RefSeq All (ncbiRefSeq) table was selected to retrieve reference annotation from UCSC Table Browser from the entire genome in GTF output format. StringTie’s merge function with default parameters in Galaxy platform was utilized to combine all the outputs from each stage [39]. The human PRDL protein sequences from our previous study were utilized for conducting tblastn search to predict and/or confirm the genomic loci of *Bos taurus* PRDL genes in bosTau9 genome [5].

### Designing of PCR primers

Putative full-length transcripts of PRDL genes containing at least one homeodomain were selected as targets for PCR amplification. Primers were designed using the NCBI Primer-BLAST tool, with untranslated regions predicted using the NCBI ORFfinder and EMBOSS Sixpack tools [41–43]. Forward and reverse primers were designed within the 5’- and 3’-UTRs, respectively, and PCR product sizes were chosen accordingly. Primer specificity was assessed using *Bos taurus* as the reference organism, and primer pairs were filtered for repeat regions using the mammalian repeat database. Primers with low self-complementarity were selected and further evaluated using the In-Silico PCR tool available in the UCSC genome browser.

### Cloning of ARGFX, DUXA, LEUTX, NOBOX, TPRX1, TPRX2, TPRX3

The amplification of *ARGFX*, *DUXA*, *LEUTX*, *NOBOX*, *TPRX1*, *TPRX2*, and *TPRX3* was carried out by PCR using 1 µl of cDNA library derived from different developmental stages of bovine oocytes or embryos. Specifically, cDNA from MII oocyte stage was used to amplify *NOBOX*, whereas cDNA from 8-cell stage embryos was employed to amplify *LEUTX*, and *TPRX3*. cDNA of the 16-cell stage was used for amplification of *TPRX1*, *TPRX2*, *ARGFX*, and *DUXA*. The PCR reaction was performed using Phusion™ High–Fidelity DNA Polymerase (ThermoFisher Scientific) in accordance with the manufacturer’s protocol. The PCR cycling conditions were as follows: an initial denaturation at 98 °C for 30 s, followed by 30 cycles of denaturation at 98 °C for 10 s, annealing at 64 °C for 30 s, and extension at 72 °C for 80 s, with a final extension at 72 °C for 10 min. The primer sequences used for amplification are listed in Table 1. The PCR products were extracted from 2% agarose gel using GeneJet Gel Extraction Kit (ThermoFisher Scientific) and were then cloned into the pCR4Blunt-TOPO vector using the Zero Blunt TOPO PCR Cloning kit (Invitrogen). Plasmid extraction was performed using the NucleoSpin Plasmid DNA Purification kit (Macherey Nagel) after inoculating individual colonies in LB medium with ampicillin (100 µg/ml) and incubating them for 12–16 h at 37 °C. The presence of inserts in the clones was verified by EcoRI digestion, and subsequently by Sanger sequencing using T7 and T3 primers (Core Facility of Genomics, Institute of Genomics, University of Tartu). Cloning of homeodomain-encoding fragments was carried out in triplicates for *ARGFX*, *DUXA*, *LEUTX*, *TPRX2*, and *TPRX3*, and in duplicates for *NOBOX*. Highly spliced *TPRX1* was represented by unique clones. Sequences were visualized on UCSC Genome Browser by Blat [44].

### Generation of PB-TetOn-bgi-TPRX3-ires-GFP-pA-PGK-Puro plasmids

The longer *TPRX3* variant inserted into pCR4Blunt-TOPO was used as a template for PCR amplification with primers containing AgeI and NotI restriction sites denoted by lower-case letters: 5’-AATATaccggtACCATGGGACCTGCAGGGAGGCAGC-3’, 5’-ATAAAgcggccgcCTATAGATTCAGACACTGCTGGGGG-3’, respectively. The PCR was performed using Phusion™ High–Fidelity DNA Polymerase (ThermoFisher Scientific) in accordance with the manufacturer’s protocol. The PCR cycling conditions were as follows: an initial denaturation at 98 °C for 30 s, followed by 30 cycles of denaturation at 98 °C for 10 s, annealing at 67 °C for 30 s, and extension at 72 °C for 80 s, with a final extension at 72 °C for 10 min. The PCR amplified *TPRX3* fragment was inserted into TetON piggyBac plasmid backbone between AgeI and NotI sites [21]. The resulting pB-TetOn-bgi-TPRX3-IRES-GFP-PGK-Puro construct was confirmed by Sanger sequencing.

### Overexpression of *TPRX3* in bovine fetal fibroblasts

Bovine fetal fibroblast (BFF) cells were grown in Dulbecco’s Modified Eagle’s Medium (DMEM, Gibco) supplemented with 10% FBS, 1% Penicillin-Streptomycin in 5% CO_2_ at 38.5 °C. Cells were split when they reached ~ 70–80% confluency using Trypsin-EDTA (0.25%). Neon Electroporation System was used for transfection of plasmids following the protocol recommended for adherent cells using 100 µl tips. One µg of pB-TetOn-bgi-TPRX3-IRES-GFP-PGK-Puro, 0.5 µg of pPB-CAG-hyPBase-IRES-Venus, and 0.5 µg of pPB-CAG-rtTA-M2-IN plasmids were used per electroporation using 1600 V 20 ms 1 pulse settings. The cells were grown for 24 h and checked for Venus expression under GFP channel with emission at 486 nm. The antibiotic selection started 24 h post electroporation in Puromycin (0.2–0.4 µg/ml) for ~ 3 days and G418 (200–400 µg/ml) for ~ 7 days after electroporation by gradually increasing concentration depending on condition of cells. In the overexpression group, cells were treated with 1 µg/ml doxycycline for 24 h, whereas in the control group cells were left untreated. Treated cells were collected 24 h following treatment.

### Preparation of RNAseq library

Two replicates of doxycycline-treated and non-treated BFF cells were harvested and processed for RNA extraction using Direct-zol™ RNA Microprep kit (Zymo Research). cDNA libraries were prepared according to the kit SMARTer^®^ Stranded Total RNA-Seq Kit v2 - Pico Input Mammalian (Takara). cDNA concentrations were assessed by Qubit Fluorometer (ThermoFisher Scientific), and 47.9 ng of cDNA library from each sample were pooled. Paired-end sequencing using the Illumina NovaSeq 6000 platform and demultiplexing were performed at Novogene.

### Reanalysis of WGS datasets

PRJNA183919, PRJNA184837, and ERX1815535 were retrieved from NCBI SRA. After exclusion of orphan reads in the retrieved sequences, the pairs were mapped to bosTau9 genome using Bowtie [45] version 1.2.3 with the parameter “--chunkmbs 1024 -a -m 1 --best --strata -S -f --ff -X 2500”.

### Prediction of ORFs, homeodomains and 9aaTAD and alignment of on UCSC Genome Browser

Each PRDL gene cDNA clone sequence in FASTA format was used as an input for running NCBI’s ORFfinder on the command line to predict ORFs [42]. The Pfam-A.hmm database (version Pfam33.1) was acquired from the FTP server hosted at EBI. The FASTA file containing the predicted ORFs was scanned against the downloaded database using hmmscan command with the --domtblout option [46]. Putative proteins with a homeodomain match were extracted. 9aaTAD regions were identified as previously described [20]. Those proteins that possessed both a homeodomain and a 9aaTAD were selected and marked in the illustration (Fig. 5B-C). For visual representation of homeodomains Simple Modular Architecture Research Tool (SMART) was used [47]. ClustalX 2.1 was used for multiple sequence alignment [48].

### RNAseq analysis

The first three nucleotides of Read 2, as well as Illumina adaptor sequences from both reads of paired-end sequencing were trimmed, and reads shorter than 18 nt were discarded using cutadapt (3.1) [49]. HISAT2 with the option --rna-strandness RF was used to map reads against bosTau9 genome [37]. The count matrix was formed using StringTie, specifying reverse (RF) strand, the reference annotation file with the -e option, and creating an output file compatible with downstream DESeq2 analysis with default options [39]. The annotation file was generated on 06.10.2023 using genePredToGtf command by pulling gene file directly from the UCSC public database and converting it to the bosTau9.ensGene.gtf file. DESeq2 in R was used for differential gene expression, in which the design formula consisted of status of doxycycline-induction and cell line, with each replicate being considered a unique cell line [50]. Non-treated samples were set as the reference for differential analysis. DESeq2 default Benjamini and Hochberg method was used to calculate adjusted P-values. For comparison, datasets from Paulson et al. [22], Lewin et al. [19], and Graf et al. [13] were reanalyzed the same way. The quantification of *TPRX3* was performed by normalizing using the median of ratios method implemented in DESeq2 [50]. Gene set enrichment analysis for biological processes was performed using genome wide annotation for bovine [51, 52]. The statistical significance of commonly upregulated genes between datasets was assessed by P-values calculated through Fisher’s Exact Test.

## Electronic supplementary material

Below is the link to the electronic supplementary material.

Supplementary Material 1: Additional file 1: Table S1. cDNA clone identifiers and their corresponding ENA accession numbers. Additional file 2: Table S2. SNVs discovered in PRDL homeobox genes and corresponding cDNA clones and residue changes. Additional file 3: Table S3. RNAseq read numbers. Additional file 4: Table S4. Significantly upregulated genes in Paulson et al and TPRX3 overexpression dataset. Additional file 5: Table S5. Significantly upregulated genes in Graf et al and TPRX3 overexpression dataset.

Supplementary Material 2: Additional file 6: Data S1. Count matrix of doxycycline-treated and non-treated samples. (.xlsx). Additional file 7: Data S2. DESeq2 results output. Additional file 8: Data S3. Differentially expressed genes in BFFs upon TPRX3 overexpression (adjusted P < 0.05).

Supplementary Material 3: Additional file 9: Figure S1. UCSC Genome Browser visualization of all investigated PRDL genes. Top track illustrates assembled transcript(s) from GV and MII oocytes and 4-cell, 8-cell, 16-cell and blastocyst stages. The transcripts are labeled with their MSTRG IDs but not with gene names on the StringTie -- merge track. Prefixes MSTRG.934, MSTRG.22530, MSTRG.21460, MSTRG.47279, MSTRG.21871, MSTRG.21873, and MSTRG.22438 in the StringTie --merge output correspond to the genes (A) ARGFX, (B) DUXA, (C) LEUTX, (D) NOBOX, (E) TPRX1, (F) TPRX2, and (G) TPRX3, respectively. Additional file 17: Figure S9. Gene-specific PCR amplification visualizations on 1.5% agarose gel. PCR reactions contained 1.5mM MgCl2 and DMSO only for TPRX1 and TPRX2 (A), and 2mM MgCl2 and DMSO (B). Amplified homeodomain-encoding PRDL cDNA fragments are marked by arrowheads. NTC: Non-template Control; 8-c: 8-cell stage; 16-c: 16-cell stage; MII: MII oocyte. Additional file 18: Figure S10. Homeodomain prediction of PRDL TFs. SMART (Simple Modular Architecture Research Tool) illustration shows ARGFX, DUXA, LEUTX, NOBOX, TPRX1, TPRX2, TPRX3 TFs with homeodomains depicted as pentagons. Pink rectangles represent low complexity regions. The relative positions of these domains in each amino acid sequence are displayed in the table on the right. Additional file 19: Figure S11. UCSC Genome Browser visualization of DUXA in human and cow genome. (A) Zoomed view of the 5’-end of the current annotation of DUXA. 1st exon (DUXA_16c_02) and 2nd exon (DUXA_16c_01) of cDNA clones derived from 16-cell stage is shown on the top track. DUXA_16c_02 cDNA clone does not cover the codon encoding predicted 1st methionine. (B) DUXA syntenic positions in human and bovine. Additional file 20: Figure S12. Dynamic expression changes of seven PRDL homeobox transcription factor genes through early bovine embryo development. Additional file 21: Figure S13. Homeodomain prediction of the clone DUXA_a_I. SMART (Simple Modular Architecture Research Tool) illustration shows DUXA_a_I with its homeodomains depicted as pentagons. The prediction does not illustrate the second homeodomain, which is disturbed by the SNV found in coding sequence. The relative position of the domain in each amino acid sequence are displayed in the table on the right. Additional file 22: Figure S14. Assessment of RNAseq data and analysis. Quality control (A) principal component analysis, (B) hierarchical clustering, and DESeq2 dispersion estimation plot (C) are plotted. Additional file 23: Figure S15. Uncropped images of agarose gels from Figure 2A and B.

Supplementary Material 4: Additional file 10: Figure S2. The prediction of ARGFX derived from Bos taurus isolate L1 Dominette 01449 registration number 42190680 breed Hereford chromosome 1, ARS-UCD2.0, whole genome shotgun sequence. Three possible ORFs for exons, but not introns, are depicted. Putative protein sequence is highlighted in yellow. Sequences from StringTie merge prediction and confirmed cDNA are drawn as lines below the corresponding sequences. Cloning primers are drawn as line arrows. Splice sites are underlined and codons split by two exons are coloured red. Hereford derived sequence was manually edited to contain the wildtype version (without the 13-bp deletion) of ARGFX, with a higher likelihood of being found in the Holstein breed. The 13-bp deletion found in reference bosTau9 genome is highlighted in purple. The homeodomain is highlighted in green.

Supplementary Material 5: Additional file 11: Figure S3. The prediction of DUXA derived from Bos taurus isolate L1 Dominette 01449 registration number 42190680 breed Hereford chromosome 18, ARS-UCD1.2, whole genome shotgun sequence. Three possible ORFs for exons, but not introns, are depicted. Putative protein sequence is highlighted in yellow. Sequences from StringTie merge prediction and confirmed cDNA are drawn as lines below the corresponding sequences. Cloning primers are drawn as line arrows. Splice sites are underlined and codons split by two exons are coloured red. The homeodomains are highlighted in green.

Supplementary Material 6: Additional file 12: Figure S4. The prediction of LEUTX derived from Bos taurus isolate L1 Dominette 01449 registration number 42190680 breed Hereford chromosome 18, ARS-UCD1.2, whole genome shotgun sequence. Three possible ORFs for exons, but not introns, are depicted. Putative protein sequence is highlighted in yellow. Sequences from StringTie merge prediction and confirmed cDNA are drawn as lines below the corresponding sequences. Cloning primers are drawn as line arrows. Splice sites are underlined and codons split by two exons are coloured red. The homeodomain is highlighted in green.

Supplementary Material 7: Additional file 13: Figure S5. The prediction of NOBOX derived from Bos taurus isolate L1 Dominette 01449 registration number 42190680 breed Hereford chromosome 4, ARS-UCD1.2, whole genome shotgun sequence. Three possible ORFs for exons, but not introns, are depicted. Putative protein sequence is highlighted in yellow. Sequences from StringTie merge prediction and confirmed cDNA are drawn as lines below the corresponding sequences. Cloning primers are drawn as line arrows. Splice sites are underlined and codons split by two exons are coloured red. The homeodomain is highlighted in green.

Supplementary Material 8: Additional file 14: Figure S6. The prediction of TPRX1 derived from Bos taurus isolate L1 Dominette 01449 registration number 42190680 breed Hereford chromosome 18, ARS-UCD1.2, whole genome shotgun sequence. Three possible ORFs for exons, but not introns, are depicted. Putative protein sequence is highlighted in yellow. Sequences from StringTie merge prediction and confirmed cDNA are drawn as lines below the corresponding sequences. Cloning primers are drawn as line arrows. Splice sites are underlined and codons split by two exons are coloured red. The homeodomain is highlighted in green.

Supplementary Material 9: Additional file 15: Figure S7. The prediction of TPRX2 derived from Bos taurus isolate L1 Dominette 01449 registration number 42190680 breed Hereford chromosome 18, ARS-UCD1.2, whole genome shotgun sequence. Three possible ORFs for exons, but not introns, are depicted. Putative protein sequence is highlighted in yellow. Sequences from StringTie merge prediction and confirmed cDNA are drawn as lines below the corresponding sequences. Cloning primers are drawn as line arrows. Splice sites are underlined and codons split by two exons are coloured red. The homeodomain is highlighted in green.

Supplementary Material 10: S10 Additional file 16: Figure S8. The prediction of TPRX3 derived from Bos taurus isolate L1 Dominette 01449 registration number 42190680 breed Hereford chromosome 18, ARS-UCD1.2, whole genome shotgun sequence. Three possible ORFs for exons, but not introns, are depicted. Putative protein sequence and homeodomain are highlighted in yellow and green, respectively. Sequences from StringTie merge prediction and confirmed cDNA are drawn as lines below the corresponding sequences. Cloning primers are drawn as line arrows. Splice sites are underlined and codons split by two exons are coloured red. The homeodomain is highlighted in green.

Supplementary Material 11: Additional file 24: Text S1. UCSC genome browser tracks for PRDL TF genes. (.pdf) Additional file 25: Text S2. UCSC genome browser track for visualizing SNVs.

## Data Availability

The cloned cDNA sequences have been deposited at the European Nucleotide Archive (ENA) with accession number PRJEB70908 (www.ebi.ac.uk/ena/browser/view/PRJEB70908). Overexpression RNAseq dataset has been deposited at ENA with the accession number PRJEB71013 (www.ebi.ac.uk/ena/browser/view/PRJEB71013). The data generated after the analysis are included in the additional files.

## References

[1] Vastenhouw NL, Cao WX, Lipshitz HD. The maternal-to-zygotic transition revisited. Development. 2019;146:dev161471. 31189646 10.1242/dev.161471

[2] Töhönen V, Katayama S, Vesterlund L, Jouhilahti EM, Sheikhi M, Madissoon E, et al. Novel PRD-like homeodomain transcription factors and retrotransposon elements in early human development. Nat Commun. 2015;6:8207. 26360614 10.1038/ncomms9207 PMC4569847

[3] Bürglin TR, Affolter M. Homeodomain proteins: an update. Chromosoma. 2016;125:497–521. 26464018 10.1007/s00412-015-0543-8 PMC4901127

[4] Bürglin TR. Homeodomain subtypes and functional diversity. Subcell Biochem. 2011;52:95–122. 21557080 10.1007/978-90-481-9069-0_5

[5] Madissoon E, Jouhilahti E-M, Vesterlund L, Töhönen V, Krjutškov K, Petropoulos S, et al. Characterization and target genes of nine human PRD-like homeobox domain genes expressed exclusively in early embryos. Sci Rep. 2016;6:28995. 27412763 10.1038/srep28995 PMC4944136

[6] Katayama S, Ranga V, Jouhilahti E-M, Airenne TT, Johnson MS, Mukherjee K, et al. Phylogenetic and mutational analyses of human LEUTX, a homeobox gene implicated in embryogenesis. Sci Rep. 2018;8:17421. 30479355 10.1038/s41598-018-35547-5 PMC6258689

[7] Jouhilahti E-M, Madissoon E, Vesterlund L, Töhönen V, Krjutškov K, Plaza Reyes A, et al. The human PRD-like homeobox gene LEUTX has a central role in embryo genome activation. Development. 2016;143:3459–69. 27578796 10.1242/dev.134510 PMC5087614

[8] Bosnakovski D, Toso EA, Ener ET, Gearhart MD, Yin L, Lüttmann FF, et al. Antagonism among DUX family members evolved from an ancestral toxic single homeodomain protein. iScience. 2023;26:107823. 37744032 10.1016/j.isci.2023.107823 PMC10514451

[9] Zou Z, Zhang C, Wang Q, Hou Z, Xiong Z, Kong F, et al. Translatome and transcriptome co-profiling reveals a role of TPRXs in human zygotic genome activation. Science. 2022;378:abo7923. 36074823 10.1126/science.abo7923

[10] Madissoon E, Damdimopoulos A, Katayama S, Krjutškov K, Einarsdottir E, Mamia K, et al. Pleomorphic Adenoma Gene 1 is needed for timely zygotic genome activation and early embryo development. Sci Rep. 2019;9:8411. 31182756 10.1038/s41598-019-44882-0 PMC6557853

[11] Zhai Y, Yu H, An X, Zhang Z, Zhang M, Zhang S, et al. Profiling the transcriptomic signatures and identifying the patterns of zygotic genome activation – a comparative analysis between early porcine embryos and their counterparts in other three mammalian species. BMC Genomics. 2022;23:772. 36434523 10.1186/s12864-022-09015-4 PMC9700911

[12] Boskovic N, Yazgeldi G, Ezer S, Tervaniemi MH, Inzunza J, Deligiannis SP, et al. Optimized single-cell RNA sequencing protocol to study early genome activation in mammalian preimplantation development. STAR Protoc. 2023;4:102357. 37314922 10.1016/j.xpro.2023.102357 PMC10277609

[13] Graf A, Krebs S, Zakhartchenko V, Schwalb B, Blum H, Wolf E. Fine mapping of genome activation in bovine embryos by RNA sequencing. Proc Natl Acad Sci U S A. 2014;111:4139–44. 24591639 10.1073/pnas.1321569111 PMC3964062

[14] Maeso I, Dunwell TL, Wyatt CDR, Marlétaz F, Veto B, Bernal JA, et al. Evolutionary origin and functional divergence of totipotent cell homeobox genes in eutherian mammals. BMC Biol. 2016;14:45. 27296695 10.1186/s12915-016-0267-0 PMC4904359

[15] Lewin TD, Royall AH, Holland PWH. Dynamic molecular evolution of mammalian homeobox genes: duplication, loss, divergence and Gene Conversion sculpt PRD class repertoires. J Mol Evol. 2021;89:396–414. 34097121 10.1007/s00239-021-10012-6 PMC8208926

[16] Tripurani SK, Lee KB, Wang L, Wee G, Smith GW, Lee YS, et al. A Novel Functional Role for the Oocyte-Specific Transcription Factor Newborn Ovary Homeobox (NOBOX) during early embryonic development in cattle. Endocrinology. 2011;152:1013–23. 21193554 10.1210/en.2010-1134 PMC3040056

[17] Boskovic N, Ivask M, Yazgeldi G, Yaşar B, Katayama S, Salumets A et al. Oxygen level alters energy metabolism in bovine preimplantation embryos. bioRxiv. 2024. 10.1038/s41598-025-95990-z PMC11965477 40175462

[18] Kõks S, Lilleoja R, Reimann E, Salumets A, Reemann P, Jaakma Ü. Sequencing and annotated analysis of the Holstein cow genome. Mamm Genome. 2013;24:309–21. 23893136 10.1007/s00335-013-9464-0

[19] Lewin TD, Fouladi-Nashta AA, Holland PWH. PRD-Class homeobox genes in bovine early embryos: function, evolution, and overlapping roles. Mol Biol Evol. 2022;39:msac098. 35512670 10.1093/molbev/msac098 PMC9117796

[20] Piskacek S, Gregor M, Nemethova M, Grabner M, Kovarik P, Piskacek M. Nine-amino-acid transactivation domain: establishment and prediction utilities. Genomics. 2007;89:756–68. 17467953 10.1016/j.ygeno.2007.02.003

[21] Gawriyski L, Jouhilahti EM, Yoshihara M, Fei L, Weltner J, Airenne TT, et al. Comprehensive characterization of the embryonic factor LEUTX. iScience. 2023;26:106172. 36876139 10.1016/j.isci.2023.106172 PMC9978639

[22] Paulson EE, Fishman EL, Schultz RM, Ross PJ. Embryonic microRNAs are essential for bovine preimplantation embryo development. Proc Natl Acad Sci U S A. 2022;119:e2212942119. 36322738 10.1073/pnas.2212942119 PMC9659414

[23] Daigneault BW, Rajput S, Smith GW, Ross PJ. Embryonic POU5F1 is required for expanded bovine blastocyst formation. Sci Rep. 2018;8:7753. 29773834 10.1038/s41598-018-25964-x PMC5958112

[24] Halstead MM, Ma X, Zhou C, Schultz RM, Ross PJ. Chromatin remodeling in bovine embryos indicates species-specific regulation of genome activation. Nat Commun. 2020;11:4654. 32943640 10.1038/s41467-020-18508-3 PMC7498599

[25] Jiang Z, Sun J, Dong H, Luo O, Zheng X, Obergfell C, et al. Transcriptional profiles of bovine in vivo pre-implantation development. BMC Genomics. 2014;15:756. 25185836 10.1186/1471-2164-15-756 PMC4162962

[26] Rodriguez-Osorio N, Wang H, Rupinski J, Bridges SM, Memili E. Comparative functional genomics of mammalian DNA methyltransferases. Reprod Biomed Online. 2010;20:243–55. 20113962 10.1016/j.rbmo.2009.11.006

[27] Santos RR, Schoevers EJ, Roelen BAJ. Usefulness of bovine and porcine IVM/IVF models for reproductive toxicology. Reproductive Biology Endocrinol. 2014;12:117. 10.1186/1477-7827-12-117 PMC4258035 25427762

[28] Zhou C, Halstead MM, Elie Bonnet-Garnier A, Schultz RM, Ross PJ. Histone remodeling reflects conserved mechanisms of bovine and human preimplantation development. EMBO Rep. 2023;24:e55726. 36779365 10.15252/embr.202255726 PMC9986824

[29] Leidenroth A, Hewitt JE. A family history of DUX4: phylogenetic analysis of DUXA, B, C and Duxbl reveals the ancestral DUX gene. BMC Evol Biol. 2010;10:364. 21110847 10.1186/1471-2148-10-364 PMC3004920

[30] Zhong YF, Holland PWH. The dynamics of vertebrate homeobox gene evolution: gain and loss of genes in mouse and human lineages. BMC Evol Biol. 2011;11:169. 21679462 10.1186/1471-2148-11-169 PMC3141429

[31] Jolma A, Yan J, Whitington T, Toivonen J, Nitta KR, Rastas P, et al. DNA-Binding Specificities Hum Transcription Factors Cell. 2013;152:327–39. 10.1016/j.cell.2012.12.009 23332764

[32] Berger MF, Badis G, Gehrke AR, Talukder S, Philippakis AA, Peña-Castillo L, et al. Variation in Homeodomain DNA binding revealed by high-resolution analysis of sequence preferences. Cell. 2008;133:1266–76. 18585359 10.1016/j.cell.2008.05.024 PMC2531161

[33] Grow EJ, Liu Y, Fan Z, Perisse IV, Patrick T, Regouski M et al. Chromatin Reprogramming of In Vitro Fertilized and Somatic Cell Nuclear Transfer Bovine Embryos During Embryonic Genome Activation. bioRxiv. 2023.

[34] Moullan N, Mouchiroud L, Wang X, Ryu D, Williams EG, Mottis A, et al. Tetracyclines disturb mitochondrial function across eukaryotic models: a call for caution in Biomedical Research. Cell Rep. 2015;10:1681–91. 25772356 10.1016/j.celrep.2015.02.034 PMC4565776

[35] Org T, Hensen K, Kreevan R, Mark E, Sarv O, Andreson R, et al. Genome-wide histone modification profiling of inner cell mass and trophectoderm of bovine blastocysts by RAT-ChIP. PLoS ONE. 2019;14:e0225801. 31765427 10.1371/journal.pone.0225801 PMC6876874

[36] Afgan E, Baker D, Batut B, Van Den Beek M, Bouvier D, Ech M, et al. The Galaxy platform for accessible, reproducible and collaborative biomedical analyses: 2018 update. Nucleic Acids Res. 2018;46:W537–44. 29790989 10.1093/nar/gky379 PMC6030816

[37] Kim D, Paggi JM, Park C, Bennett C, Salzberg SL. Graph-based genome alignment and genotyping with HISAT2 and HISAT-genotype. Nat Biotechnol. 2019;37:907–15. 31375807 10.1038/s41587-019-0201-4 PMC7605509

[38] Li H, Handsaker B, Wysoker A, Fennell T, Ruan J, Homer N, et al. The sequence Alignment/Map format and SAMtools. Bioinformatics. 2009;25:2078–9. 19505943 10.1093/bioinformatics/btp352 PMC2723002

[39] Pertea M, Pertea GM, Antonescu CM, Chang TC, Mendell JT, Salzberg SL. StringTie enables improved reconstruction of a transcriptome from RNA-seq reads. Nat Biotechnol. 2015;33:290–5. 25690850 10.1038/nbt.3122 PMC4643835

[40] Karolchik D, Hinricks AS, Furey TS, Roskin KM, Sugnet CW, Haussler D, et al. The UCSC table browser data retrieval tool. Nucleic Acids Res. 2004;32:D493–6. 14681465 10.1093/nar/gkh103 PMC308837

[41] Ye J, Coulouris G, Zaretskaya I, Cutcutache I, Rozen S, Madden TL. Primer-BLAST: a tool to design target-specific primers for polymerase chain reaction. BMC Bioinformatics. 2012;13:134. 22708584 10.1186/1471-2105-13-134 PMC3412702

[42] Sayers EW, Barrett T, Benson DA, Bolton E, Bryant SH, Canese K, et al. Database resources of the National Center for Biotechnology Information. Nucleic Acids Res. 2011;39:D38–51. 21097890 10.1093/nar/gkq1172 PMC3013733

[43] Rice P, Longden L, Bleasby A. EMBOSS: the European Molecular Biology Open Software suite. Trends Genet. 2000;16:276–7. 10827456 10.1016/s0168-9525(00)02024-2

[44] Kent WJ. BLAT – the BLAST-like alignment tool. Genome Res. 2002;12:656–64. 11932250 10.1101/gr.229202 PMC187518

[45] Langmead B, Trapnell C, Pop M, Salzberg SL. Ultrafast and memory-efficient alignment of short DNA sequences to the human genome. Genome Biol. 2009;10:R25. 19261174 10.1186/gb-2009-10-3-r25 PMC2690996

[46] Finn RD, Clements J, Arndt W, Miller BL, Wheeler TJ, Schreiber F, et al. HMMER web server: 2015 update. Nucleic Acids Res. 2015;43:W30–8. 25943547 10.1093/nar/gkv397 PMC4489315

[47] Letunic I, Khedkar S, Bork P. SMART: recent updates, new developments and status in 2020. Nucleic Acids Res. 2021;49:D458–60. 33104802 10.1093/nar/gkaa937 PMC7778883

[48] Larkin MA, Blackshields G, Brown NP, Chenna R, Mcgettigan PA, McWilliam H, et al. Clustal W and Clustal X version 2.0. Bioinformatics. 2007;23:2947–8. 17846036 10.1093/bioinformatics/btm404

[49] Martin M. Cutadapt removes adapter sequences from high-throughput sequencing reads. EMBnet J. 2011;17:10–2.

[50] Love MI, Huber W, Anders S. Moderated estimation of Fold change and dispersion for RNA-seq data with DESeq2. Genome Biol. 2014;15:550. 25516281 10.1186/s13059-014-0550-8 PMC4302049

[51] Wu T, Hu E, Xu S, Chen M, Guo P, Dai Z, et al. clusterProfiler 4.0: a universal enrichment tool for interpreting omics data. Innov. 2021;2:100141. 10.1016/j.xinn.2021.100141 PMC8454663 34557778

[52] Carlson M. org.Bt.eg.db: Genome wide annotation for Bovine. R package version 3.8.2. 2019.

