# Supplementary material for: Molecular cloning of PRD-like homeobox genes expressed in bovine oocytes and early IVF embryos
Source: BMC Genomics. 2024 Nov 6;25:1048. doi: 10.1186/s12864-024-10969-w (PMC11542365; doi:10.1186/s12864-024-10969-w)
Supplement: Supplementary file 3 — Supplementary Material 3: Additional file 9: Figure S1. UCSC Genome Browser visualization of all investigated PRDL genes. Top track illustrates assembled transcript(s) from GV and MII oocytes and 4-cell, 8-cell, 16-cell and blastocyst stages. The transcripts are labeled with their MSTRG IDs but not with gene names on the StringTie -- merge track. Prefixes MSTRG.934, MSTRG.22530, MSTRG.21460, MSTRG.47279, MSTRG.21871, MSTRG.21873, and MSTRG.22438 in the StringTie --merge output correspond to the genes (A) ARGFX, (B) DUXA, (C) LEUTX, (D) NOBOX, (E) TPRX1, (F) TPRX2, and (G) TPRX3, respectively. Additional file 17: Figure S9. Gene-specific PCR amplification visualizations on 1.5% agarose gel. PCR reactions contained 1.5mM MgCl2 and DMSO only for TPRX1 and TPRX2 (A), and 2mM MgCl2 and DMSO (B). Amplified homeodomain-encoding PRDL cDNA fragments are marked by arrowheads. NTC: Non-template Control; 8-c: 8-cell stage; 16-c: 16-cell stage; MII: MII oocyte. Additional file 18: Figure S10. Homeodomain prediction of PRDL TFs. SMART (Simple Modular Architecture Research Tool) illustration shows ARGFX, DUXA, LEUTX, NOBOX, TPRX1, TPRX2, TPRX3 TFs with homeodomains depicted as pentagons. Pink rectangles represent low complexity regions. The relative positions of these domains in each amino acid sequence are displayed in the table on the right. Additional file 19: Figure S11. UCSC Genome Browser visualization of DUXA in human and cow genome. (A) Zoomed view of the 5’-end of the current annotation of DUXA. 1st exon (DUXA_16c_02) and 2nd exon (DUXA_16c_01) of cDNA clones derived from 16-cell stage is shown on the top track. DUXA_16c_02 cDNA clone does not cover the codon encoding predicted 1st methionine. (B) DUXA syntenic positions in human and bovine. Additional file 20: Figure S12. Dynamic expression changes of seven PRDL homeobox transcription factor genes through early bovine embryo development. Additional file 21: Figure S13. Homeodomain prediction of the clone DUXA_a_I. SMART [file 12864_2024_10969_MOESM3_ESM.pdf]

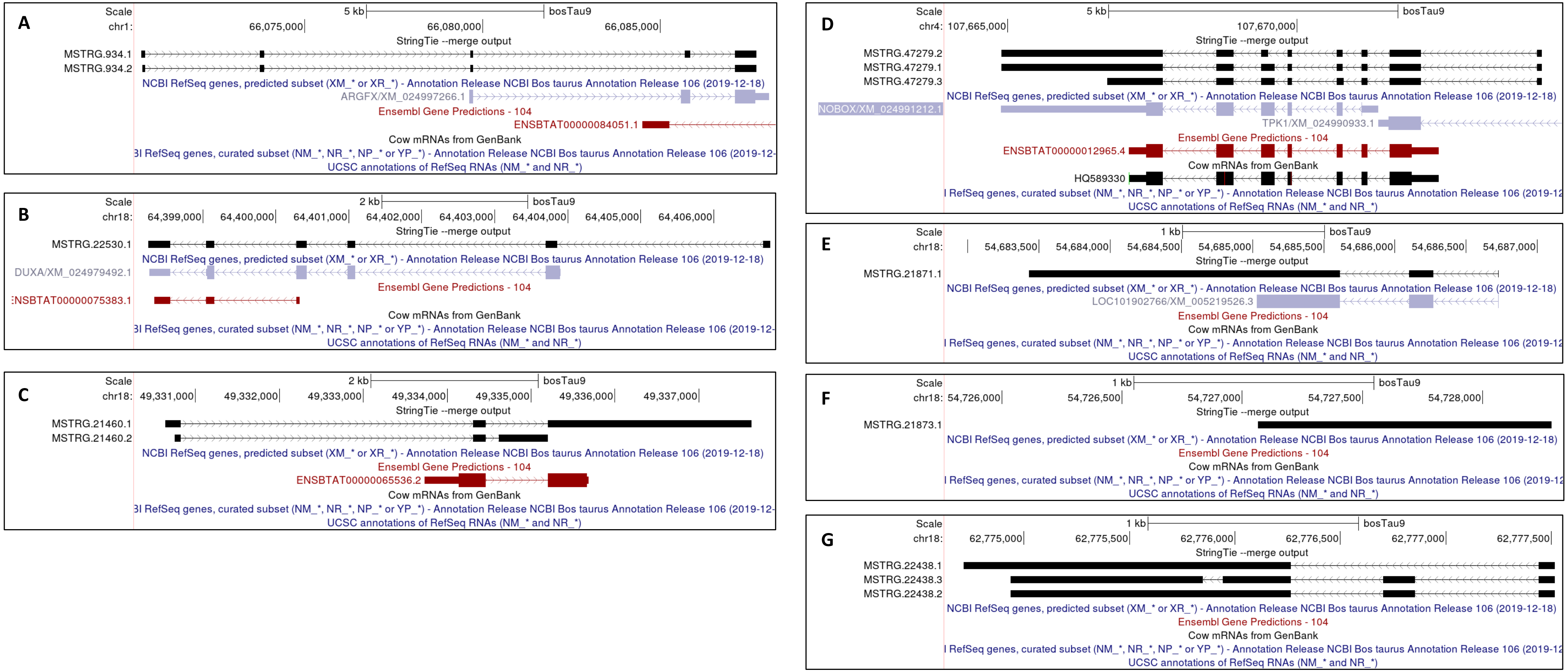

Supplementary Figure S1. UCSC Genome Browser visualization of all investigated PRDL genes. Top track illustrates assembled transcript(s) from GV and MII oocytes and 4-cell, 8-cell, 16-cell and blastocyst stages. The transcripts are labeled with their MSTRG IDs but not with gene names on the StringTie --merge track. Prefixes MSTRG.934, MSTRG.22530, MSTRG.21460, MSTRG.47279, MSTRG.21871, MSTRG.21873, and MSTRG.22438 in the StringTie --merge output correspond to the genes **(A)** *ARGFX*, **(B)** *DUXA*, **(C)** *LEUTX*, **(D)** *NOBOX*, **(E)** *TPRX1*, **(F)** *TPRX2*, and **(G)** *TPRX3*, respectively.

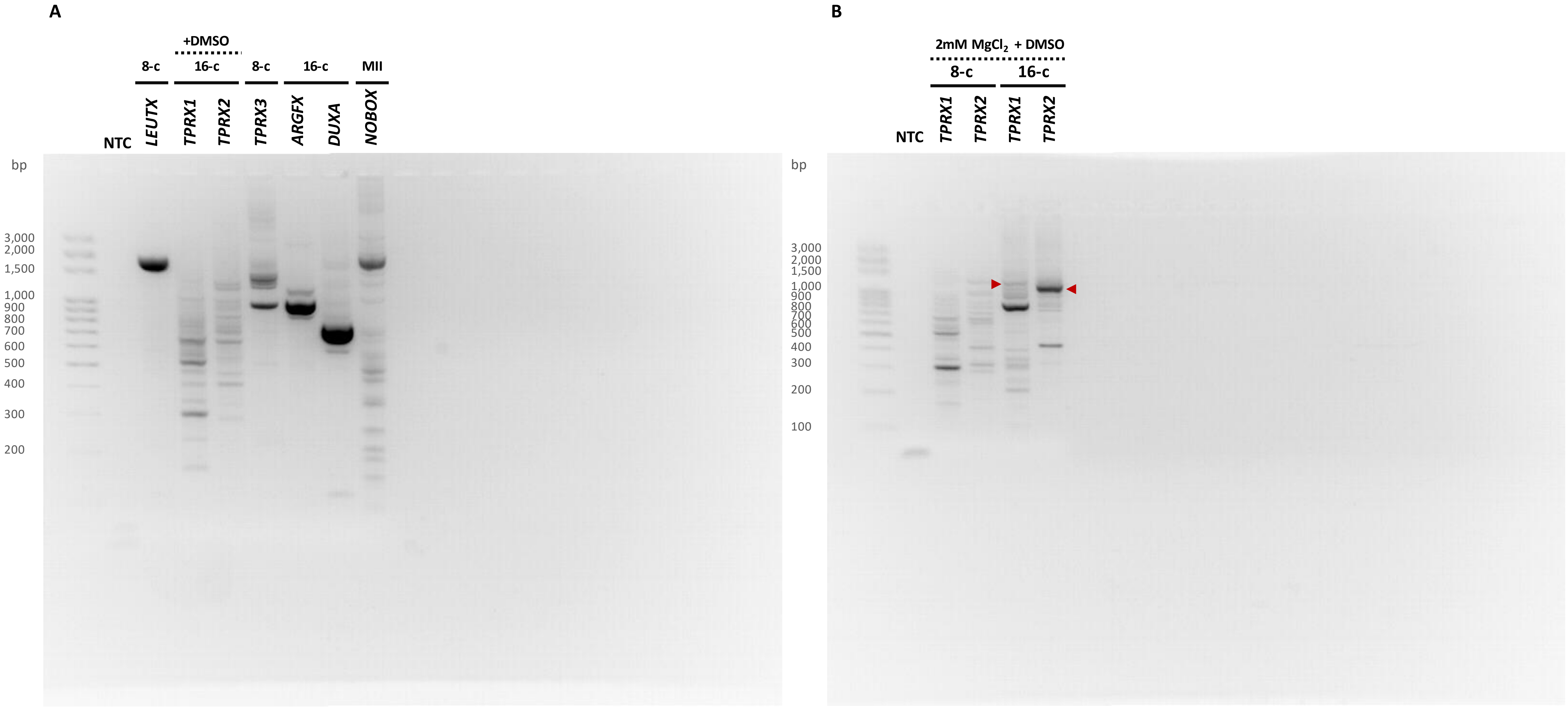

Supplementary Figure S9. Gene-specific PCR amplification visualizations on 1.5% agarose gel. PCR reactions contained 1.5mM $MgCl_2$ and DMSO only for *TPRX1* and *TPRX2* **(A)**, and 2mM $MgCl_2$ and DMSO **(B)**. Amplified homeodomain-encoding PRDL cDNA fragments are marked by arrowheads. NTC: Non-template Control; 8-c: 8-cell stage; 16-c: 16-cell stage; MII: MII oocyte.

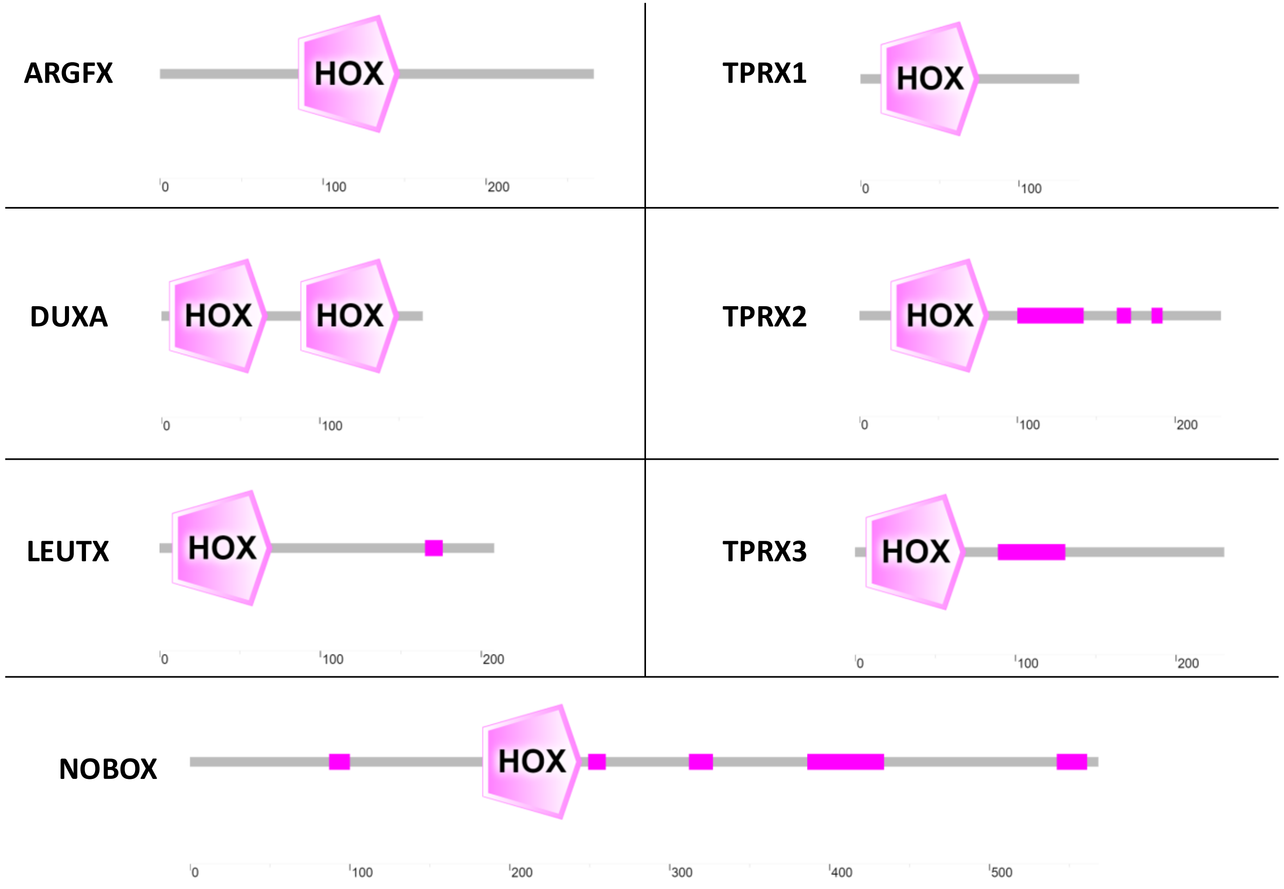

Protein	Domain	Start residue	End residue	E-value
ARGFX	Homeodomain	85	147	1.74E-17
DUXA	Homeodomain	5	67	8.26E-19
	Homeodomain	88	150	7.35E-25
LEUTX	Homeodomain	8	70	5.58E-16
NOBOX	Homeodomain	183	245	1.37E-24
TPRX1	Homeodomain	13	75	4.64E-22
TPRX2	Homeodomain	20	82	2.69E-18
TPRX3	Homeodomain	7	69	2.97E-20

Supplementary Figure S10. Homeodomain prediction of PRDL TFs. SMART (Simple Modular Architecture Research Tool) illustration shows ARGFX, DUXA, LEUTX, NOBOX, TPRX1, TPRX2, TPRX3 TFs with homeodomains depicted as pentagons. Pink rectangles represent low complexity regions. The relative positions of these domains in each amino acid sequence are displayed in the table on the right.

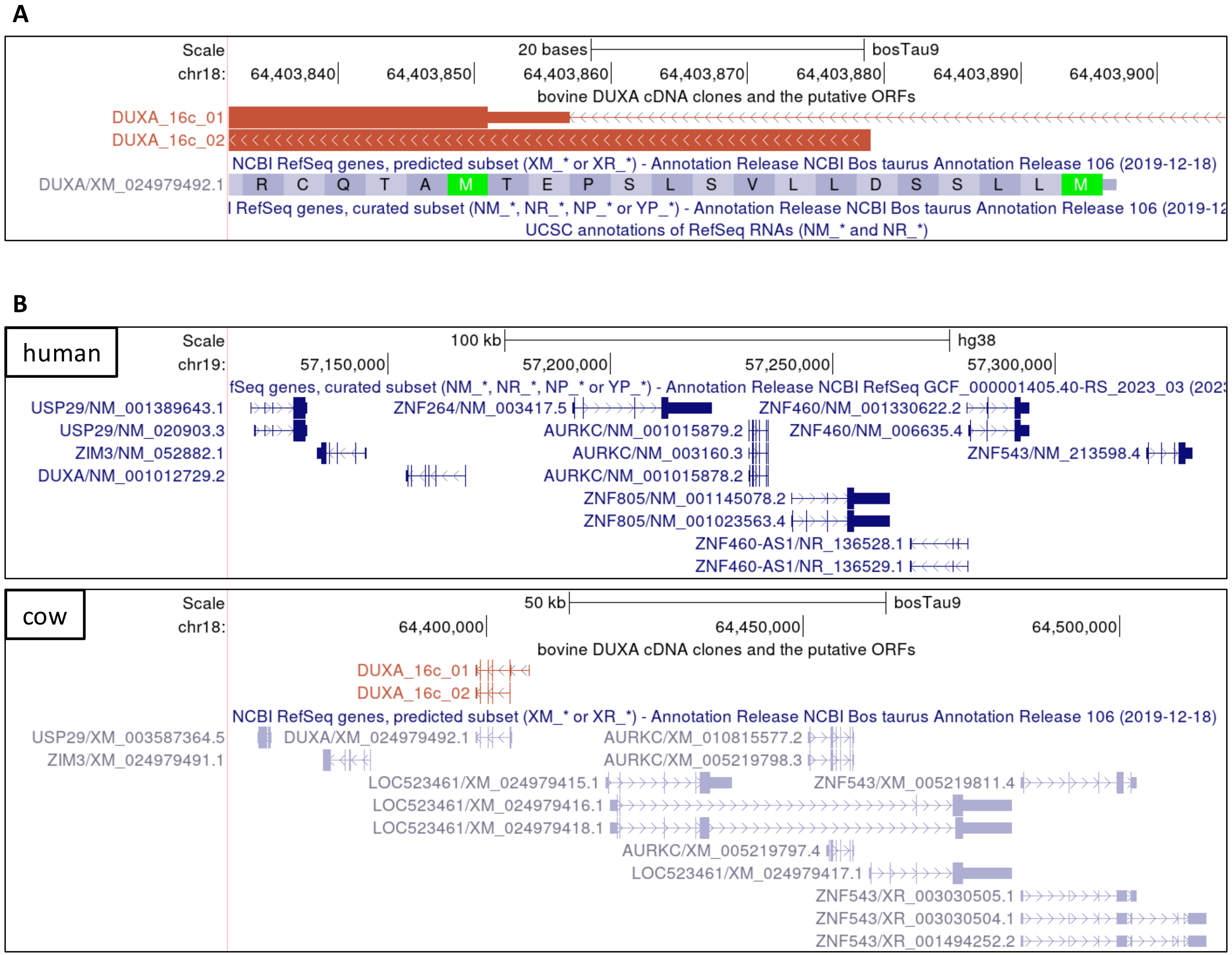

Supplementary Figure S11. UCSC Genome Browser visualization of *DUXA* in human and cow genome. (A) Zoomed view of the 5′-end of the current annotation of *DUXA*. 1st exon (DUXA_16c_02) and 2nd exon (DUXA_16c_01) of cDNA clones derived from 16-cell stage is shown on the top track. DUXA_16c_02 cDNA clone does not cover the codon encoding predicted 1st methionine. **(B)** *DUXA* syntenic positions in human and bovine.

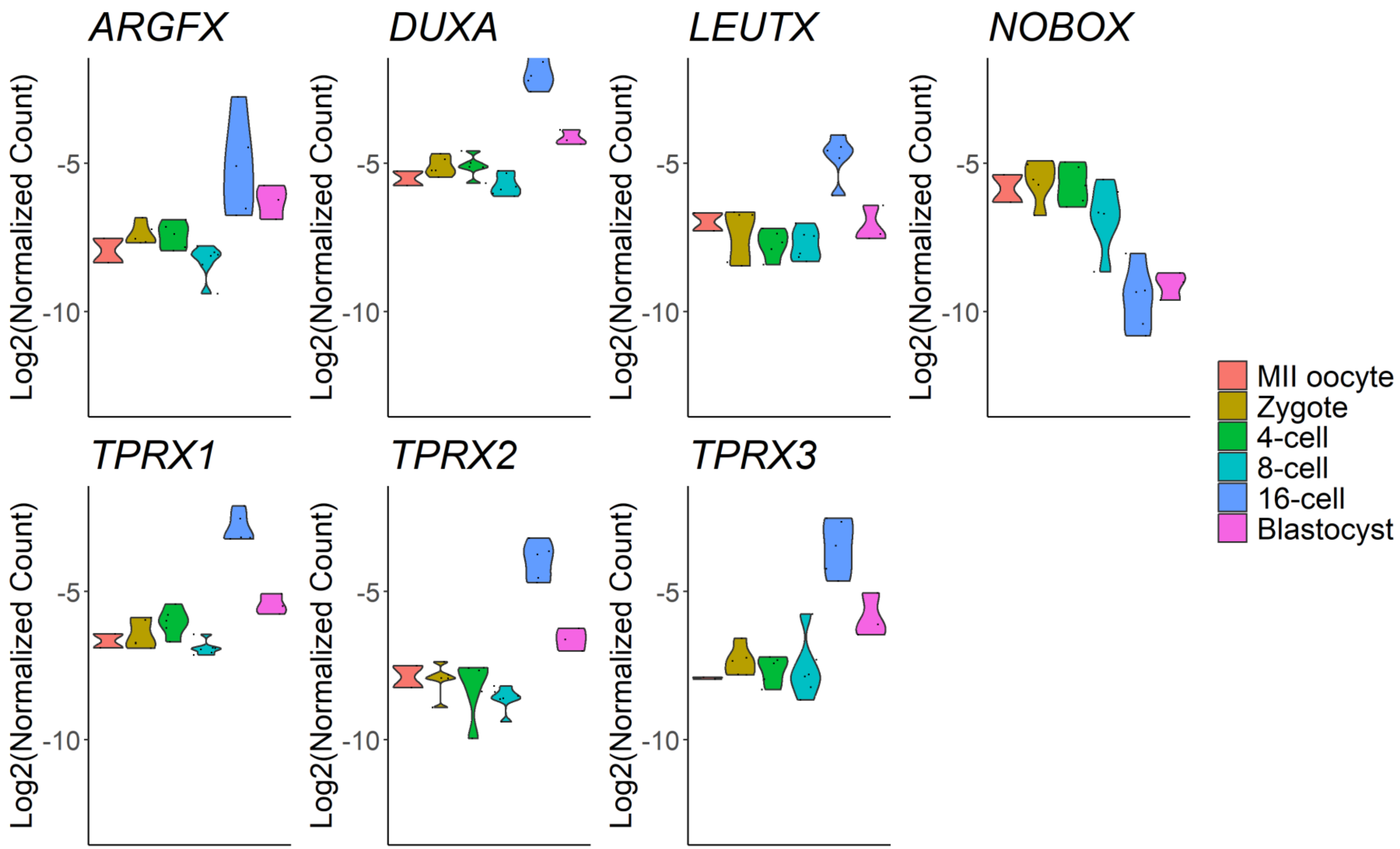

Supplementary Figure S12. Dynamic expression changes of seven PRDL homeobox transcription factor genes through early bovine embryo development.

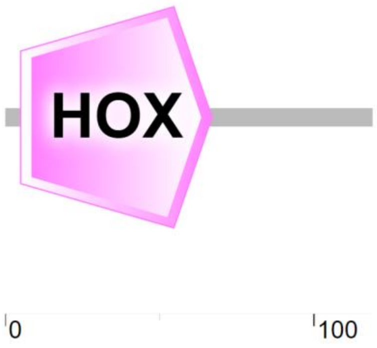

Protein	Domain	Start residue	End residue	E-value
DUXA_a_I	Homeodomain	5	67	8.26E-19

Supplementary Figure S13. Homeodomain prediction of the clone DUXA_a_I. SMART (Simple Modular Architecture Research Tool) illustration shows DUXA_a_I with its homeodomains depicted as pentagons. The prediction does not illustrate the second homeodomain, which is disturbed by the SNV found in coding sequence. The relative position of the domain in each amino acid sequence are displayed in the table on the right.

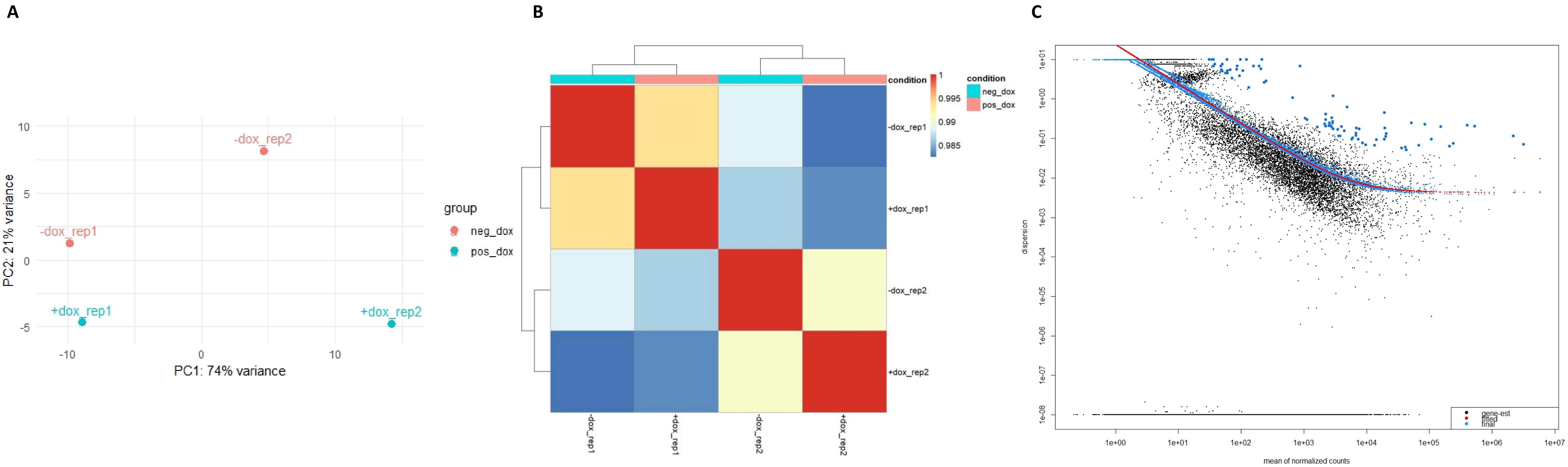

Supplementary Figure S14. Assessment of RNAseq data and analysis. Quality control **(A)** principal component analysis, **(B)** hierarchical clustering, and DESeq2 dispersion estimation plot **(C)** are plotted.

A

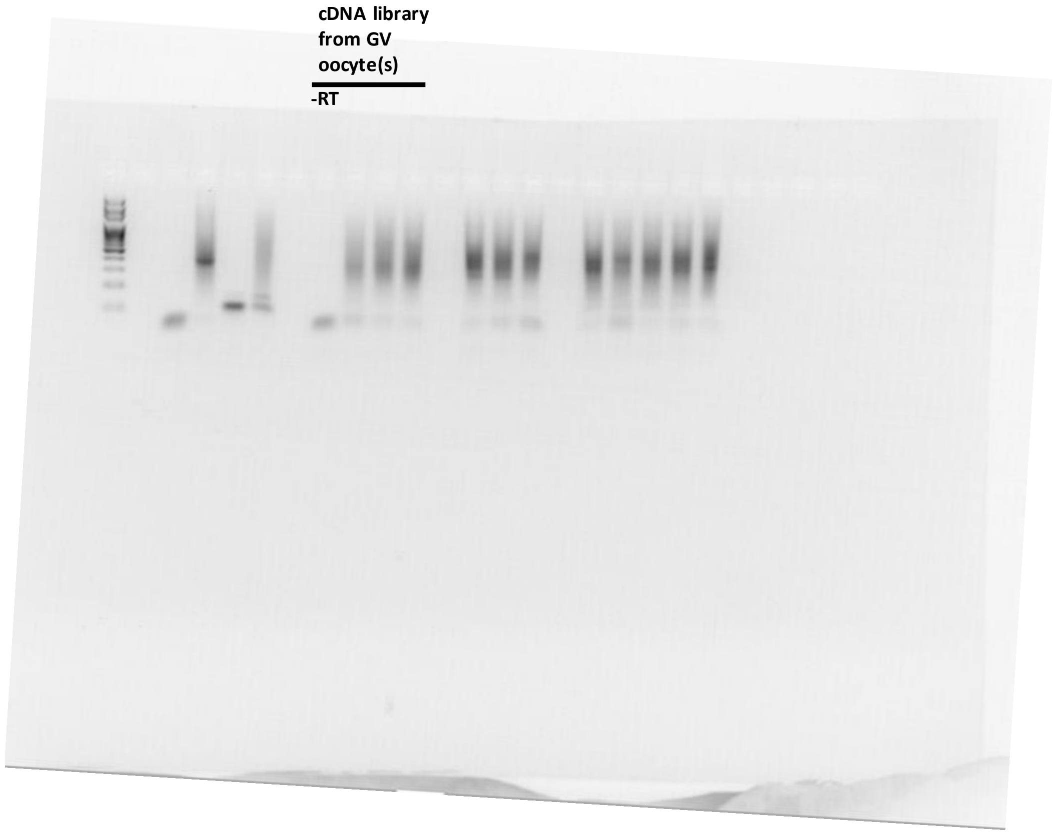

B

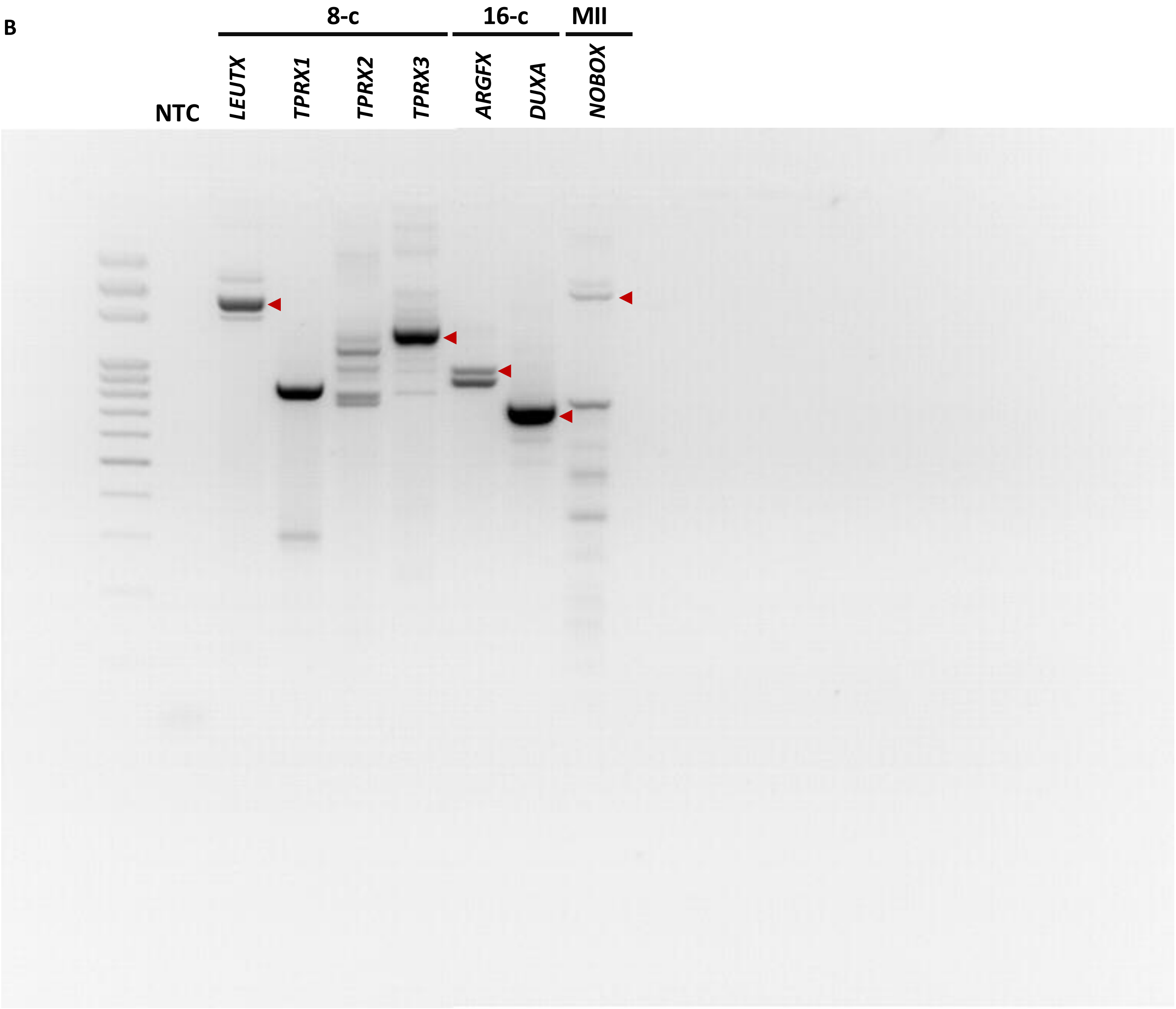

Supplementary Figure S15. Uncropped images of agarose gels from Figure 2A and B.
