# Supplementary material for: Molecular cloning of PRD-like homeobox genes expressed in bovine oocytes and early IVF embryos
Source: BMC Genomics. 2024 Nov 6;25:1048. doi: 10.1186/s12864-024-10969-w (PMC11542365; doi:10.1186/s12864-024-10969-w)
Supplement: Supplementary file 4 — Supplementary Material 4: Additional file 10: Figure S2. The prediction of ARGFX derived from Bos taurus isolate L1 Dominette 01449 registration number 42190680 breed Hereford chromosome 1, ARS-UCD2.0, whole genome shotgun sequence. Three possible ORFs for exons, but not introns, are depicted. Putative protein sequence is highlighted in yellow. Sequences from StringTie merge prediction and confirmed cDNA are drawn as lines below the corresponding sequences. Cloning primers are drawn as line arrows. Splice sites are underlined and codons split by two exons are coloured red. Hereford derived sequence was manually edited to contain the wildtype version (without the 13-bp deletion) of ARGFX, with a higher likelihood of being found in the Holstein breed. The 13-bp deletion found in reference bosTau9 genome is highlighted in purple. The homeodomain is highlighted in green. [file 12864_2024_10969_MOESM4_ESM.pdf]

Supplementary Figure S2. The prediction of *ARGFX* derived from *Bos taurus* isolate L1 Dominette 01449 registration number 42190680 breed Hereford chromosome 1, ARS-UCD2.0, whole genome shotgun sequence. Three possible ORFs for exons, but not introns, are depicted. Putative protein sequence is highlighted in yellow. Sequences from StringTie merge prediction and confirmed cDNA are drawn as lines below the corresponding sequences. Cloning primers are drawn as line arrows. Splice sites are underlined and codons split by two exons are coloured red. Hereford derived sequence was manually edited to contain the wildtype version (without the 13-bp deletion) of *ARGFX*, with a higher likelihood of being found in the Holstein breed. The 13-bp deletion found in reference bosTau9 genome is highlighted in purple. The homeodomain is highlighted in green.

```
      K  L  Y  L  N  T  G  Y  F  T  I  R  T  H  L  R  N  C  I  K  R  H  I  N  Q  L  E  N  G  H  S  K  G     F1
       S  Y  I  *  I  Q  G  I  S  P  *  G  H  T  *  G  T  A  *  R  G  T  *  I  S  L  K  M  G  T  P  R  E    F2
     Q  V  I  F  K  Y  R  V  F  H  H  K  D  T  L  K  E  L  H  K  E  A  H  K  S  A  *  K  W  A  L  Q  G  N   F3
5001 CAAGTTATATTTAAATACAGGGTATTTCACCATAAGGACACACTTAAGGAACTGCATAAAGAGGCACATAAATCAGCTTGAAAATGGGCACTCCAAGGGA 5100
     ----:----|----:----|----:----|----:----|----:----|----:----|----:----|----:----|----:----|----:----|
            Cloning primer ----------------------->
StringTie merge ---------------------------------------------------------------------------------------------
                ARGFX_16c_01 ---------------------------------------------------------------------------

     I  H  D                                                                                              F1
      F  M  T                                                                                             F2
       S  *  Q                                                                                            F3
5101 ATTCATGACAGGTGAGCATGGGAAGTGGTGGAGGGGAGGGCTCCAAAATGTGGGGTTTAAGTGGTCAAAAAGGATTTAAAGACCAAAGGAGCAAGATTA 5200
     ----:----|----:----|----:----|----:----|----:----|----:----|----:----|----:----|----:----|----:----|
     -----------
     -----------

5201 AGGATGCAAATCGGGAACTTCCCTGGTGGCACAGTGGATGGGAATGGCCTTCCAATGCAAGGAATGCGGGTTCAGCCTCACATACAGTGGAGCAGCAAAA 5300
     ----:----|----:----|----:----|----:----|----:----|----:----|----:----|----:----|----:----|----:----|

5301 CCCAAGCACCACAAGTGCTGAACCCTCGCTCTAGAGCCCAGGGAACCATCACTACTGAAGCCCACGTGCCTAAAGCCTGTGCCTAAAGCCACAGCAATGA 5400
     ----:----|----:----|----:----|----:----|----:----|----:----|----:----|----:----|----:----|----:----|

5401 GAAGCCCAAGCATTGGAGCTAGACAGTAGCTCCCACTCGCCGCAACTAGAGAAAGCCTGTGGGCAGCAATGAAGACCAGTGCAGCCAAAAACAAAAACAA 5500
     ----:----|----:----|----:----|----:----|----:----|----:----|----:----|----:----|----:----|----:----|

5501 AATTATTTTAAAAAAAAGAGGCAAATCACTCAGCTCCATCCCCACTGGAGGGGACTGATACAGCATTGTAAATTGAGGACAGAAGAAAGATCAGGTGATC 5600
     ----:----|----:----|----:----|----:----|----:----|----:----|----:----|----:----|----:----|----:----|

5601 TGATTGCTTTCTGAAGCTCAGGGAGTAAATGTGAGACTCCTTGTTATGAAGGTTGGTGTCTGCTTCACAGGGTTCACGTACTTCCCCAGTTTGTGCTAGC 5700
     ----:----|----:----|----:----|----:----|----:----|----:----|----:----|----:----|----:----|----:----|

5701 CTGGACTTTGAAAAGTCCTCGTTAGACTGTTCATCATCAGAAGGTCCATCATAGAAGCAGTTTTTGTATGTTTTGTTCACTGCAGAATCATCAGGCTGAT 5800
     ----:----|----:----|----:----|----:----|----:----|----:----|----:----|----:----|----:----|----:----|
```

```
5801 AAGAACAGGCTGTATAAGGCAGGCAATAAATATTTGCTGAGTGAGTGCTGTCCGTGGCATTTTCCAGGCTAGAATACTGGAGTATGTTGCCATTTTCTTC 5900
     ----:----|----:----|----:----|----:----|----:----|----:----|----:----|----:----|----:----|----:----|

5901 TCCAGGGGATCTTCCCGACCCAAGAATCGAACCCGTATCTCCCTCATTGGCAGGCAGGTTCTTTATTACTGAGCCACCAGGGGAAGCACTGATGAAAGCT 6000
     ----:----|----:----|----:----|----:----|----:----|----:----|----:----|----:----|----:----|----:----|

6001 ACGCTTCTTTGTTTAATATGCCAGCTTAAGCTGTAGACCCAGGCTTCCCATGTGGCACTAGTAGTAAAGAACCCGCTTGCCAATGCAGGAGACTCAAGAG 6100
     ----:----|----:----|----:----|----:----|----:----|----:----|----:----|----:----|----:----|----:----|

6101 ATGTGGGTTCGATCCCTGAATCGGGACGATCCCTTGGAGAAGGAAATGGCAACCCACCCCAGTATTTTTTCCTGGAGAATCCCATGGACAGAGGAGCCTG 6200
     ----:----|----:----|----:----|----:----|----:----|----:----|----:----|----:----|----:----|----:----|

6201 TCAGGCTATAGTCCATGGGGTTGCGAAGAGTCAGACATGACTGAGGCAACTTAGAATGTACAAGGTGTAGACTCTCAGGATACTCTTCCTGGGGAAGTGT 6300
     ----:----|----:----|----:----|----:----|----:----|----:----|----:----|----:----|----:----|----:----|

6301 GTGGGGATTTATATTCTGCACACAGGAGTTAATAAAACCTTCTTTTGCAGTTTGTCTAGCTGTACAGACTTTAAGGTCTTAATTTTCCTTCATTTATAAG 6400
     ----:----|----:----|----:----|----:----|----:----|----:----|----:----|----:----|----:----|----:----|

6401 AGGATACTAACAACTGTACTTCTCCCCATGTGTTTGTTGAGGTTTAAATGAATCACGTATATGAAGCATTTAGATGTCTGACATGGAAAACATCTCCAAA 6500
     ----:----|----:----|----:----|----:----|----:----|----:----|----:----|----:----|----:----|----:----|

6501 TATTATTATATTCATCAGATATTTACTATTCCTGTGGCTCTTCACTCCTAAAGTTCCAAGTTTCCCTTTAGCCTGAAGAACTTGCTTTAATATTTCTAAA 6600
     ----:----|----:----|----:----|----:----|----:----|----:----|----:----|----:----|----:----|----:----|

6601 GAACAGGACTAATGGCCACAGACCTGATAGTTTCTCTTAATCTGATAATGTCATCATTGCACCTTCATTTTTTTTTAAGATTTTTTTTTAATGTGGGCTA 6700
     ----:----|----:----|----:----|----:----|----:----|----:----|----:----|----:----|----:----|----:----|

6701 TTTATAAAGTCTTTATTGAATTTGTTACAGTATTGTTTATGTTTTACACTTTGGTTCTTTGGCCATGAGGCATGTGGAATCTTAGCAACCCAACCCCTGT 6800
     ----:----|----:----|----:----|----:----|----:----|----:----|----:----|----:----|----:----|----:----|

6801 GCATTGGAAGGCTAAGTCTTAACCACTAAACTACCAGGAAAGTCCTTCACTGAAAGATATTTTTCCAGATATACAATTATTAGTTGGCAATTCTTTTACC 6900
     ----:----|----:----|----:----|----:----|----:----|----:----|----:----|----:----|----:----|----:----|

6901 TCAGTACTTTAGAGATTTTGTTCTACTGTCTTCCTCAGTTTCTGATGAGAAATCTAAGGTCATTTGACCTAGTCTACTTGTCTAATTTGTCCATCTTTTC 7000
     ----:----|----:----|----:----|----:----|----:----|----:----|----:----|----:----|----:----|----:----|

7001 ATGTATGGACTGTTTCAACAATCTTACTGAAGAAAATATCCCCTTACTCTGTCACAAAGATGTTGACATACATTTTTTTAAGTTTATTTTTAATTGGAGG 7100
     ----:----|----:----|----:----|----:----|----:----|----:----|----:----|----:----|----:----|----:----|

7101 ATAATTGTTTTATAATATTGTGTTGGTTTCTGCCATATATCAACATGACTCAGCCATTGGTATATGTATGCCCCCTCCTTCTTAAATCTCCGTCCCACCT 7200
     ----:----|----:----|----:----|----:----|----:----|----:----|----:----|----:----|----:----|----:----|

7201 CCCACTCCATCCTACCCCTCCAGGATATCACAGAGCCCTGGATTTGAGCTTCCTGCCTCATACAATACATTTCCACTGGCTGTCTAATTTTACTCATGGT 7300
     ----:----|----:----|----:----|----:----|----:----|----:----|----:----|----:----|----:----|----:----|
```

```
7301 AATCTATATGTTTCAGCATTCATCACCCCCTCTCCTTCCCCCACCATGAGCCCACAAGTCTGTTCTCTGTGTCTGCATCTCCACTGCTGCCCTGCAGATA 7400
     ----:----|----:----|----:----|----:----|----:----|----:----|----:----|----:----|----:----|----:----|

7401 GGTTCATCAGTATCATCTTTTGAGATTTCATATATATGCATTAATACATGCTATTTGTCTTTTTCTGACTTACTTCACTCTATATAAATAGGCTCTTGGT 7500
     ----:----|----:----|----:----|----:----|----:----|----:----|----:----|----:----|----:----|----:----|

7501 TCATCCACCTCATTGAACTGACTCAAATGCAATTCCCTTTTACGGCTGAGTAATATTCCAGTGTGTATATGCACCACAACTTCTCTATTCATCTGTCGAT 7600
     ----:----|----:----|----:----|----:----|----:----|----:----|----:----|----:----|----:----|----:----|

7601 GGGCATCTAGGTTGCTTCCATGTCCTAGCTACTATAAATAGTGCTGCAATGAATGTTGGGGTGCATCTGTCTTTCTCAGTTATGGTTTCCTCAGGGTATA 7700
     ----:----|----:----|----:----|----:----|----:----|----:----|----:----|----:----|----:----|----:----|

7701 TGCCCAGTAGTGGGACTGTTGGGTCATATGGTACGTAGTTTTATTCCTATTTTTTTTAAGGACTCTCCATTCTGTTTTCTGTAGTGGTTGTATCAGTTTG 7800
     ----:----|----:----|----:----|----:----|----:----|----:----|----:----|----:----|----:----|----:----|

7801 CATTCCACCAACAGCGCAAGAAAGTTTCCTTTTCTGCACACCCTCTCCAGTATTTATTGTTTGATGATGGACCTTCTGACTGGTGTGAGGTGGTACCTCA 7900
     ----:----|----:----|----:----|----:----|----:----|----:----|----:----|----:----|----:----|----:----|

7901 TTATAGTTTTGATTTGAATTTCTCTAATAATGAGTGATGTTGAGAGTCTTTCCATGTGTTGATTAGCTATCTGTATGTCTTTAGAGAAATGTCTGTTTAG 8000
     ----:----|----:----|----:----|----:----|----:----|----:----|----:----|----:----|----:----|----:----|

8001 GTATTCTGCCCACTTTTTGATTGGGTTGTTCATTTTTCTGGTATTGAGTTGAATGAGCTACTTGTTTATTTTGGAGATTAATCCTTTGTCAGTTGTTTCA 8100
     ----:----|----:----|----:----|----:----|----:----|----:----|----:----|----:----|----:----|----:----|

8101 TTTACTATTATTTTCTCCCATTCTGAGGGTTTGGCACACATTTTCTTTTAATACTTCCAAAGTTTCATTTTTGATTAATACATCTGGAGTATTTTTCTAT 8200
     ----:----|----:----|----:----|----:----|----:----|----:----|----:----|----:----|----:----|----:----|

8201 GGTATAAAATGTGAATCATTTCTATCAGAGGTGGAGACTCCAGGGGTTAACCTTATTTGTCAGAGATTGCTACAGTAGACCTAGTCCTCTTGAAAACGTT 8300
     ----:----|----:----|----:----|----:----|----:----|----:----|----:----|----:----|----:----|----:----|

                                                      F  P  L  P  D  I  S  T  P  D  E  Q  K  S  P  R  E       F1
                                                       F  P  C  Q  T  S  A  P  L  M  N  K  R  V  Q  E  N      F2
                                                     I  S  L  A  R  H  Q  H  P  *  *  T  K  E  S  K  R  I     F3
8301 GCTTTCTAGTGAATGAATGATTTCATCAGTGTCTTCTTTCTCATCAAGATTTCCCTTGCCAGACATCAGCACCCCTGATGAACAAAAGAGTCCAAGAGAA 8400
     ----:----|----:----|----:----|----:----|----:----|----:----|----:----|----:----|----:----|----:----|

     S  P  A  R  P  F  C  L  H  G  *  F  *  H  E  L  P  G  *  F  K  D  T  S  W  C                             F1
      P  L  P  D  P  F  V  Y  M  D  D  S  S  T  N  F  Q  D  D  S  K  I  P  A  G  A                            F2
       P  C  Q  T  L  L  S  T  W  M  I  L  A  R  T  S  R  M  I  Q  R  Y  Q  L  V  P                           F3
8401 TCCCCTGCCAGACCCTTTTGTCTACATGGATGATTCTAGCACGAACTTCCAGGATGATTCAAAGATACCAGCTGGTGCCAGTGAGTGTAACTCTCCTACC 8500
     ----:----|----:----|----:----|----:----|----:----|----:----|----:----|----:----|----:----|----:----|
```

```
8501 CTGTTGCCCTCTTTACATGGGACCCTCTAATTGTATTTTGGATTTGTTTTAAAATAGTTATTTATTATATCTTTTCTTGTTTTTGTTTTTTATTTCAACT 8600
     ----:----|----:----|----:----|----:----|----:----|----:----|----:----|----:----|----:----|----:----|

8601 CTGTTTTGCATTGAGGTATAGCCAATTAACAATGTTACGATAGTTTCAGGTGAACAGCAAAGGGACTCAGCCATACATATACATACATGAATCTATAACA 8700
     ----:----|----:----|----:----|----:----|----:----|----:----|----:----|----:----|----:----|----:----|

8701 CTGTGTACCTGATCTTCCCAATAGGACTTCTCTGATAGCTCAGTTGGTAAAGAATCCACCCACAATGCCAAGAGATCCCAGGTCAATTCCTGGGTCAGGA 8800
     ----:----|----:----|----:----|----:----|----:----|----:----|----:----|----:----|----:----|----:----|

8801 AGATCTGCTGGAAAAGGGATAGGCTAGCCACTCCAGTATTCTTGGGCTTCCCTTGTGGCTCAGCTGGTAAAAAAAAAAACTGCAGTGCAGGAGATCTGGG 8900
     ----:----|----:----|----:----|----:----|----:----|----:----|----:----|----:----|----:----|----:----|

8901 TTTGATCCCTGGGTTGGGAAGATCCCCTGGGGAAGGGAACAGCTACCCACTCCAGTATTCTGGCCTGGAAAATTCCATGGACTGTATAGTCCATGGGGTC 9000
     ----:----|----:----|----:----|----:----|----:----|----:----|----:----|----:----|----:----|----:----|

9001 GCAGAGAGTAGGACACAACTTAGCAATTTTCACTTCTTTTTTTTCACGTGATCTTCCCAAGTGAAATGAAAGTAGGGCCCTAACTATTCCTTCCCCTCCC 9100
     ----:----|----:----|----:----|----:----|----:----|----:----|----:----|----:----|----:----|----:----|

9101 TGGCAACCATAAGTTTGTTTTCTAAGTCTGTGAGTCTCTTTCTGTTTTGTCAGTTCATTTGTATCATTTTTTTTTTTAGATTCCAAATATAAAAAATTTC 9200
     ----:----|----:----|----:----|----:----|----:----|----:----|----:----|----:----|----:----|----:----|

9201 ATATGATATTTCTCCTTAAGGTCTATGTTTCCTTATTGATTTTCTGTCTGATCTGTCCATTGATGAAAATGGGATATTAAAGTCCCCTGCTATTATTGTG 9300
     ----:----|----:----|----:----|----:----|----:----|----:----|----:----|----:----|----:----|----:----|

9301 TTACTGTCAATTTCTCCCTTTATGTTTGTTAATATTTGCCTTACATATTAAGGTGCCCCTGTGTTGGATATGTATATAATTGTCATATCTTCTTCTTGGA 9400
     ----:----|----:----|----:----|----:----|----:----|----:----|----:----|----:----|----:----|----:----|

9401 TTTAACCCTTCATGATTATGTAGTATCTTTGTTTCTTATAACAGTCTTCATTTTAAAGTCTATTTTGTCTCATATGAGTATTGCTATGCCAGCTTTCTTA 9500
     ----:----|----:----|----:----|----:----|----:----|----:----|----:----|----:----|----:----|----:----|

9501 TGATTTCCATTTGCATGAAATACCTTCCCCCATCCCCTTACTTTCAGTCTGTAAGTGTCTGTAGGTCTGAGGTGGGTCTCTTGTAGACAACACATATATG 9600
     ----:----|----:----|----:----|----:----|----:----|----:----|----:----|----:----|----:----|----:----|

9601 GATCTTGTTTTTGTATCCTCCCAGCCATTCTATGTCTTTTAATTAGTGCATTTAATCCATTTGAAAGAAAGTGAAAGTCATTCAGTCATGTCCAGCCCTT 9700
     ----:----|----:----|----:----|----:----|----:----|----:----|----:----|----:----|----:----|----:----|

9701 TGCGACCCCATAGACTCTATGGTCCATGGAATTCTCTAGGGCAGAATACTGGAGTGGGTAGCCGTTCCCTTCTCCAGGGGATCTTCTCAACCTAGGGATC 9800
     ----:----|----:----|----:----|----:----|----:----|----:----|----:----|----:----|----:----|----:----|

9801 AATCCCAGCTTTCCTGCACTGCAGGCAGATTCTTTACCAGCTGAGCCACCAGGGAAGCCCTTTAATCCATTTACATTTAAGGTAATTATCTATATGTGTG 9900
     ----:----|----:----|----:----|----:----|----:----|----:----|----:----|----:----|----:----|----:----|

9901 ATCCTATTATCATTTTGTTCATTGTTTGGGGTTTATTTTTTGTAGATCTTTCCCTCCTCTTGTGTTTCCTGCCTGGAGAAGTTCCTTTAGCATTTGTTGT 10000
```

```
      ----:----|----:----|----:----|----:----|----:----|----:----|----:----|----:----|----:----|----:----|

10001 AAAACTGGTTTAGTGGTGCTGAATTCTCTTAACTTTGTCTGGAAAGCTTTTGGTTTTGCCATTGGATTTGAAGGACAGTCTTGCTGGGTAGAGTACTCTT 10100
      ----:----|----:----|----:----|----:----|----:----|----:----|----:----|----:----|----:----|----:----|

10101 GGTTGTAGGGTCTTCCCTTTCATCACTTGGAATATATTGTGCCATTCCCTTCTGGTTTGTAGAGTTTCTGTTGAGAAATCAGTTGATAATATTATGGGAT 10200
      ----:----|----:----|----:----|----:----|----:----|----:----|----:----|----:----|----:----|----:----|

10201 TTCCCTTGTATGTTGTCTTTTTTCCCTTGTTGCTTTCAATATCTTATCTTTGTCTTTAGTTTTTGTCAGTTTGATTAGTATGTGTCTTGGGGTGTGTTCC 10300
      ----:----|----:----|----:----|----:----|----:----|----:----|----:----|----:----|----:----|----:----|

10301 TCCTTGGGTTTATCCTGCCTGGGTCCCTCTGAGCTTCCTGGACCTGGCTGACTATTTCCTTTCCCATGTTGGGGAAGTTTTCAGCTATTATCTCTTCAAA 10400
      ----:----|----:----|----:----|----:----|----:----|----:----|----:----|----:----|----:----|----:----|

10401 TATTTTCTCAGGGTTGTTCTCTCTCTCTTCTTCTGAGACACCTATAATGTGAATATTGGTGCATTTAATGTTGTTCCAGAGTCCTCTTAGGCTGTCTTTA 10500
      ----:----|----:----|----:----|----:----|----:----|----:----|----:----|----:----|----:----|----:----|

10501 TTTCTTTTCATTCTTCTATATTCTGTTTTACAACAATGATTTCCACCATTTCGCCTTCCAGGTCATTTATCCATTCCACTTATCTAGGATTTATCTTGTT 10600
      ----:----|----:----|----:----|----:----|----:----|----:----|----:----|----:----|----:----|----:----|

10601 CCTTCATCTGGGACACAATCCTATTTCTTATTTTGGTTAGCTTTCTGTGATTGTGGTACTAGGTCTGGAAGCTATGGGATTGTAGTTCTTGTGCCCTCTG 10700
      ----:----|----:----|----:----|----:----|----:----|----:----|----:----|----:----|----:----|----:----|

10701 GTGGATGAGGATAAGAGGCTTGTGCAAGCTTCCTGATGGGAGGGACCAGTTGTGGGGATAACTGGGTCTTGCTCTGGTGAGCATGCTCAGTAAAACTTTA 10800
      ----:----|----:----|----:----|----:----|----:----|----:----|----:----|----:----|----:----|----:----|

10801 ATCCAATTGTCTGCTGATGGGTGGGGTTGCACTCCCTCCCTGTTAGTTGTTTTGCCTGAGGTAACCCAGTCCTGGAGTGTGCAGGCTCTGTGGTATGGCT 10900
      ----:----|----:----|----:----|----:----|----:----|----:----|----:----|----:----|----:----|----:----|

10901 ATTGGCAACCTCCAAGAGGATTTACACCTGACTGCTGCTGCCAGTGCCCCGTCCCCATGGTGGGCAATTGCTGATCCACAGCTCTGCAGGAGATCCTCAG 11000
      ----:----|----:----|----:----|----:----|----:----|----:----|----:----|----:----|----:----|----:----|

11001 ACACTCACATGTAGGTCTGACTCACTGTTCTATGGGGTTAGTGTTCCTTTCCCCTGGGTCCTGGTGCACATAAGGTTTGTTTGTGCCCTCCAAGAGTGGA 11100
      ----:----|----:----|----:----|----:----|----:----|----:----|----:----|----:----|----:----|----:----|

11101 GTCTCTGTTTCCCCCAGTCCTATGGAGGTCCTGTAACCAAATTCCACTGGCCTTCAATGTCAGATTCCCTAGGAATTCTCAGCACTTTTGCTGATCCCCA 11200
      ----:----|----:----|----:----|----:----|----:----|----:----|----:----|----:----|----:----|----:----|

11201 AGCTGGGGAGCCTGATGTGGGGCTTAGAACCTTCACAACAGTGCGAGAACTTCTTTGGTATTATTGTTCTCCAGTTTGTCGGTCACCCGACAGGTATGCT 11300
      ----:----|----:----|----:----|----:----|----:----|----:----|----:----|----:----|----:----|----:----|

11301 GCTGCTGCTCCTGCTAAGTTGCTTCAGTTGTGTCCGCCCCACCAGCCTCCTCCATCCCTGGGATTCTCCAGGCAAGAATACTGGAGTGGGCACCCAACAG 11400
      ----:----|----:----|----:----|----:----|----:----|----:----|----:----|----:----|----:----|----:----|
```

```
11401 GTATAGGATTTGATTTTATCGTGATTGTGCCCCTTTTACCATCTTGTTGGGGTTTCTCCGTTGTCCTTGGACATGGGGTATCTTTTCTTGGTGGGTTCCA 11500
      ----:----|----:----|----:----|----:----|----:----|----:----|----:----|----:----|----:----|----:----|

11501 ATGTCTTTCTGTCTATGGATGTTCAACAACTAGTTGTGATTTTGGTGCTCTTGGTTTTGGGGTTTTGTTCATTTGTTTGTTTGGCCCTAGCCAGGAACCC 11600
      ----:----|----:----|----:----|----:----|----:----|----:----|----:----|----:----|----:----|----:----|

11601 TGCAGTGGAAGTGCAGAGTCCTAACCACTAGGAAGCCAGGGAATTCCCTGTTATATCTCTTATATATCTTTTCAAAAATATATTCAAAAACTATTCCCAT 11700
      ----:----|----:----|----:----|----:----|----:----|----:----|----:----|----:----|----:----|----:----|

11701 CATAAAGAGGGAAGTTATAAATTTCATTTCAAGTGCCTTTGGCCCATGTCTAATCAAATTATGATAAACTGAGAACACAAGATATACAGCAAGTGAGAAT 11800
      ----:----|----:----|----:----|----:----|----:----|----:----|----:----|----:----|----:----|----:----|

11801 GTTTTTCTAGAAGAGAAAGACTGAAGCAAAGAGAAAGAAAAATACAGAAAGCTTCTTTTCCAAGGCAGAATACAATCCTGACACTTCTGATAATGTGTTA 11900
      ----:----|----:----|----:----|----:----|----:----|----:----|----:----|----:----|----:----|----:----|

11901 TGCAGGTTTTTTTCCCTCATGTTTCTCCTGACACCAGCTAGGTATCCTACAATTCAACTCAACTCTGACACTATCTACCCAGAGAAAGTATTAGATTCCA 12000
      ----:----|----:----|----:----|----:----|----:----|----:----|----:----|----:----|----:----|----:----|

12001 TAGGTTAAGAGCTTAGTCCCAAAGACTGCCCTTACTTCAGTCACAAGACCAGGTTGTCTGTGCTTCTAATCAACTGGCTATAAATCAGAGGTTCCCATGA 12100
      ----:----|----:----|----:----|----:----|----:----|----:----|----:----|----:----|----:----|----:----|

12101 CACCCTCCTTGGGTTTCATTAATTTGCAAGTGGCTCAGAGATCTCAGGAAATCTATTTACTTAACCTGAGTACCATTTTATTACAAAAGATATTAAAGAA 12200
      ----:----|----:----|----:----|----:----|----:----|----:----|----:----|----:----|----:----|----:----|

12201 TAAGAATCAACAGCTGGATGAAGACATACATAGGGCTCAAACAAAGGAGCTTCTGTGCTCATGAGTTTAGATCTGGCTAGGCAGCCTGTGGATGTGATCT 12300
      ----:----|----:----|----:----|----:----|----:----|----:----|----:----|----:----|----:----|----:----|

12301 GCTTCATCAATGTAGAGGCTGTCTGAACCCTCCCATCCTTTTTGGTTTTTTAATGGAAGCTTCATCACATAGTCTTAATTGATTGAATCACTGCCCATTG 12400
      ----:----|----:----|----:----|----:----|----:----|----:----|----:----|----:----|----:----|----:----|

12401 GTGACTGATTCAAACTCCAGCCCCTCTTCCCTCCCTAAAAATCATGATGTAGGGTTGAAAGTTCCAGTCCTGTAATCAGGGTTGGTTCCCTGGGCAACTA 12500
      ----:----|----:----|----:----|----:----|----:----|----:----|----:----|----:----|----:----|----:----|

12501 GTCCCCCATCTTTAGAAGATTTCTAAAAGTCACCTCATTAACAAAGTCAGTTAAGGTCACTTCATTAACATGAAGTCAGTTGCGGTTGAAAGGAGCCTAA 12600
      ----:----|----:----|----:----|----:----|----:----|----:----|----:----|----:----|----:----|----:----|

12601 TGTGAATATCAAGACACTCATTTCACCTTGATGGCTCTGGAGAAATTTCAAAAACTGAGGAAAAAAGACAGAAGTATTATAACAAAAGATGCTCCCATTG 12700
      ----:----|----:----|----:----|----:----|----:----|----:----|----:----|----:----|----:----|----:----|

12701 CTCTAACGGCTTAGGAAATTCTAAGAATTTGGGGAGTTGTGAGCCAGGAGCCATGGACAAAGACTAAAATATATATGTGTTATACATCAAAATATCACAC 12800
      ----:----|----:----|----:----|----:----|----:----|----:----|----:----|----:----|----:----|----:----|

12801 AAGAATATTGTGGTCCAATATTGTGGGTAAACAGGAAAAATATCATTTTATATAGAGAGAGTAGTTGTGTATTAATACATACACAGATTTATTTTTATAA 12900
      ----:----|----:----|----:----|----:----|----:----|----:----|----:----|----:----|----:----|----:----|
```

```
12901 ATTGTATGTTAATAACAGAACTCTCATTTTATTCAGGCGGCAAAGTAAACAATTTTAAAGTTTTCCTAGTCAAAGTTGTATCTTGATGAATTCTGAAATA 13000
      ----:----|----:----|----:----|----:----|----:----|----:----|----:----|----:----|----:----|----:----|

13001 TGTTCAGACAGTCCCTGACTTATGATGGTTCTACTGATGATTTTTCAACTTTTATGTTTTATGATGGTGAAAACCAATATGCATTCAGTAGAAACCATAC 13100
      ----:----|----:----|----:----|----:----|----:----|----:----|----:----|----:----|----:----|----:----|

13101 TTCAGTTTTGAATCTTGATATTTTCCCGGGTTAGAGACTAAAGAGTACAGAAATACACAGACAGACATTCTGTGTGCTTGATTGGAAGACAAAATTGTTA 13200
      ----:----|----:----|----:----|----:----|----:----|----:----|----:----|----:----|----:----|----:----|

13201 AAGATGCCAATACTACTCAAAGTAGTCTATAACTGGTAGCACTGTGGTATTGGGCAACGTTTCTTCAGACTTGGCTCAGAGTTACAATGCATTTAGCATA 13300
      ----:----|----:----|----:----|----:----|----:----|----:----|----:----|----:----|----:----|----:----|

13301 TTAAAGCTGACAACATGGAGACTTTTTGCAGGCCTCCCCAGACAGTGGAGTTTTCCTGATAATTTGCCACATTAGGTTATAAAATATTTCATTAACAGTT 13400
      ----:----|----:----|----:----|----:----|----:----|----:----|----:----|----:----|----:----|----:----|

13401 ATTTTCTACTTTGCAGAGGAGTCTAAGAACGTGCAGTTGTTCCATTTTCTTGCTAGACTTCAACTTGTTCCTTTCTTACAACTCTGTCATCTTCCACGTC 13500
      ----:----|----:----|----:----|----:----|----:----|----:----|----:----|----:----|----:----|----:----|

13501 ACACTTACTACCAACCAGAATTTTGGAACACCATCAGTGTCTTTAACTTAAAGAATCTGTTCTCTCTGATCTTGTGTATCATTAAATGTGAACTGTGCTG 13600
      ----:----|----:----|----:----|----:----|----:----|----:----|----:----|----:----|----:----|----:----|

13601 TGTAGGAATAAAATAAAGCAAAGCCTTGTCCATGTTTCATGTACAAATCTATCATCACTGTAAATGGCCTATTTCTATATTATCCAAGATTTCAAGCGTA 13700
      ----:----|----:----|----:----|----:----|----:----|----:----|----:----|----:----|----:----|----:----|

13701 TACTGTTTTGCATCTACTTTAACTTGCTTTCTATAATAATCTTCTATGGTAGGATTATATTTTCAACAAAAATTCCTTGAATAAAGTGTACAGTGAGAGT 13800
      ----:----|----:----|----:----|----:----|----:----|----:----|----:----|----:----|----:----|----:----|

13801 CAATTTTCCAATGTCCCCTGAACCAAGAACTGCTAGCTTATACTCACACATAGTGCAAACTTGTAAGAGCCAGTACCTGTCACACCATCCCCGGGGCCCT 13900
      ----:----|----:----|----:----|----:----|----:----|----:----|----:----|----:----|----:----|----:----|

13901 GCACCCAAGAGCCTTGAGCTCCCTCTGTGTCGATGCTTCTGTCACCAAGTCTGTCCTGTTGCTGCAGCTGCCAGGGCTGGTTTGTTTAGAAGCCTGACTC 14000
      ----:----|----:----|----:----|----:----|----:----|----:----|----:----|----:----|----:----|----:----|

14001 TGGGCAAGTTTTGCCCTCTCTTCTCAATTTCAGGATCAGCTTGTCCAGCTCAGTAAAGATTTCTACTTAGATGTTGATTAAGATTAAAATGAAATTATGT 14100
      ----:----|----:----|----:----|----:----|----:----|----:----|----:----|----:----|----:----|----:----|

14101 GGTTATTTGGAAGGGGAAGTTGACATTTTTAATGTTTAGTCATTTAAGAATATATTTCTCTATTCCAATCACTTATGCTCTTTAAAACAATTTTATAGCT 14200
      ----:----|----:----|----:----|----:----|----:----|----:----|----:----|----:----|----:----|----:----|

                                                                                         T  F  F  I  T  T     F1
                                                                                          L  F  S  S  R  P    F2
                                                                                        D  F  F  H  H  D      F3
14201 TATTTCATCTAGTCCTTATACATTTCATTAAAATGAATTCTTAGGCATTTTATAATATGTATGTTGATATTATTGTACATAGGACTTTTTTCATCACGAC 14300
```

```
      ----:----|----:----|----:----|----:----|----:----|----:----|----:----|----:----|----:----|----:----|

       Y  D  V  W  N  L  N  S  P  T  R  D  Q  T  R  A  F  D  S  E                                 F1
        T  T  C  G  I  L  T  P  Q  P  G  I  K  P  G  P  S  T  V  K                                F2
      L  R  R  V  E  S  *  L  P  N  Q  G  S  N  P  G  L  R  Q  *  K                               F3
14301 CTACGACGTGTGGAATCTTAACTCCCCAACCAGGGATCAAACCCGGGCCTTCGACAGTGAAAGGTATAAATTTGTTAGTTTTAAAGAAAGTATACTTCCT 14400
      ----:----|----:----|----:----|----:----|----:----|----:----|----:----|----:----|----:----|----:----|

14401 GTTCATATTATATTGGGAAATAGGAGTGAGCAATATGGTTTTTTGATTTTGTTTTTAGTATTACTTAGTTTTTATTTCAGTAAATATGTGGGAATCCATC 14500
      ----:----|----:----|----:----|----:----|----:----|----:----|----:----|----:----|----:----|----:----|

14501 AAATATTTGTTTGAAGAAAGAAACTTTTGATATGCAACCCCTTTCCCTCAACATATTAGATTTTTTAAAATATTTTAATTTTATTAGAGTATAGTTGATT 14600
      ----:----|----:----|----:----|----:----|----:----|----:----|----:----|----:----|----:----|----:----|

14601 TACAATACTGTATTAGTTTCAGGTGTACAGCAAAATGATTCATTTATACATATATTCATTCTTTTTCAGAGTCTTTTCTCATATAGGTTATCACAGAATA 14700
      ----:----|----:----|----:----|----:----|----:----|----:----|----:----|----:----|----:----|----:----|

14701 TAGGATAGGGTTCCCTGGGCTGCACAGCAGGTCTCCATCAGTCATCTGTCTTATATATAGCAGTGTGTGTGTGTGTGTGTGTGTGTGTGTGTGTGTGTGC 14800
      ----:----|----:----|----:----|----:----|----:----|----:----|----:----|----:----|----:----|----:----|

14801 TCATCCCACATGCAATAAGTCTTATAGAAGAATTATTTTCTATAAAACCACTTAGCTAATTAACTGACAACACTTTCCTAATTTACACTTGATATGCTGA 14900
      ----:----|----:----|----:----|----:----|----:----|----:----|----:----|----:----|----:----|----:----|

14901 GTAGGTTAATTCTTATAAAAGATCAGTAGAGTCATGGGCAATGAACTAATTATCAGAAATGTGATGAAGTTACATAGTCCATGTTAAATGTAGTTTGTAA 15000
      ----:----|----:----|----:----|----:----|----:----|----:----|----:----|----:----|----:----|----:----|

15001 CTTCAAGCAAAGATTTCCGTTTTACTGACAAATATCCTACAATAACAAAATTAATAAAACATTAATGCAGTACATGTTATGTGGTATGTATTATTGTTTC 15100
      ----:----|----:----|----:----|----:----|----:----|----:----|----:----|----:----|----:----|----:----|

15101 ATGTGTAGTAATGACTTAAATTAATACATTCATGTAATACATTACAATTCTTATTATAAAGTAAACAGACATTGAGAGCATTCCCTTTGCTTGATGAAAC 15200
      ----:----|----:----|----:----|----:----|----:----|----:----|----:----|----:----|----:----|----:----|

15201 TAAGGTGAAGAGTTGAACTAATCTGTGATCACATTCCTAGAAAACTACATTTTGTAATAAAATTTCTACTATTAAAGTATTACACTCAATATAACAGCAA 15300
      ----:----|----:----|----:----|----:----|----:----|----:----|----:----|----:----|----:----|----:----|

15301 ATTTGGAAAACAGCAGTGGCCACAAACTGGAAAAGGTCCGTTTTCATTCCAATCCCAAAGAAGGGCAATGCCAAAGAATATTCAAATTGCCACACAGTTG 15400
      ----:----|----:----|----:----|----:----|----:----|----:----|----:----|----:----|----:----|----:----|

15401 CTCGCCTTTCACATGCCACCAAGGTAATGCTCAAAATCCTTCAAGCTAGGCTTCAACAGTATCTGAACCAAGAACTTACAGATGTAAAAGCTGGATTTAG 15500
      ----:----|----:----|----:----|----:----|----:----|----:----|----:----|----:----|----:----|----:----|
```

```
15501 AAAAGGCAGAAGAACCAGAGATAAAATTTCCAATATCTGTTGGATCATAGAAAAAGCAAGAGAGTTCCAGAAAAACATCTACTTCTGCTTTATTGACTAT 15600
      ----:----|----:----|----:----|----:----|----:----|----:----|----:----|----:----|----:----|----:----|

15601 GCTAAAGCCTTTGATTATGTAGATCACAACAAACTGGAAAATTCTTCAAGAGATGGGAAGACCAGACCACCTTCCCTGCCTCCTGAGAAACCTGTATGAA 15700
      ----:----|----:----|----:----|----:----|----:----|----:----|----:----|----:----|----:----|----:----|

15701 GGTCAAGAAGCAACAGTTAGAACCAGACATGGAACAATGGATTGGTTCCAAATTGGGAAAGGAGTACGTCAAAGTTGTATATTGTCACCATGCTTATTTA 15800
      ----:----|----:----|----:----|----:----|----:----|----:----|----:----|----:----|----:----|----:----|

15801 ACTTCTATACAGAGTACATCATGTGAAATTCAGGGCTGGGTGAAGCACAAGCTGGAATCAAGATTGCCAGAAATATCAACAACCTCAGATATGTAGATGA 15900
      ----:----|----:----|----:----|----:----|----:----|----:----|----:----|----:----|----:----|----:----|

15901 CACCACCCTTATGGCAGAAAGTGAAGAGGAACTAAAGAGCCTCTTGATGAAAGTGAGAGGAGAGTGTAAAAGCTGGCTTAAAACTTAACATTCAGAAAAC 16000
      ----:----|----:----|----:----|----:----|----:----|----:----|----:----|----:----|----:----|----:----|

16001 TAAGATCATGGCATTTGGTCCCATTACTTCATGGCAAATAGATGGGGAAACAGTGGAAACAGTGTCAGACTTTATTTTCTTGGGCTCCAAAATCAGTTTG 16100
      ----:----|----:----|----:----|----:----|----:----|----:----|----:----|----:----|----:----|----:----|

16101 GATGGTGATTGCAGCCATGAAATTAAAAGATGCTTGCTTCTTGGAAGAAAAGCTATGACAAACCTAGACAGCATATTAAAAAGCAGAGACATTACTTTGC 16200
      ----:----|----:----|----:----|----:----|----:----|----:----|----:----|----:----|----:----|----:----|

16201 TGACAAAGGTCTGTATAGTCAAAGCTATAGTTTTTCCAGTGGTAATGTATGGATGTGAGAGTTGGACCATAAAAAAGGCTGAGCACCAAAGAATCCATGC 16300
      ----:----|----:----|----:----|----:----|----:----|----:----|----:----|----:----|----:----|----:----|

16301 TTTCGAACTGTGGTGTGGAGAAGACTTTTGAAAGTCCCTTGAGCTGCGAGGAGATCAAGCCAATCACTCCTAAAGGAAATCAACCCTGAATATTCATGGA 16400
      ----:----|----:----|----:----|----:----|----:----|----:----|----:----|----:----|----:----|----:----|

16401 AGGATTGATGATGAAGCTGAAGCCCCAATACTTTGGCCACCTGATGCGAAGAGCCGACTCATTGGAGAAGACCCTGACACTGGGAAAAGATTGAGGGCAG 16500
      ----:----|----:----|----:----|----:----|----:----|----:----|----:----|----:----|----:----|----:----|

16501 GAGGAGAAGGGGATGACAGAGGATGAGATGGTTGGATGGCATCACCAAATCAATCGACATGAGTTTGAGCAAACTCCAGGAGATAGTGAAGGACAGACAG 16600
      ----:----|----:----|----:----|----:----|----:----|----:----|----:----|----:----|----:----|----:----|

16601 GGACTGGCATACTGCTGTGCATGGGGTCACAAAGAGCTGGACACAACCAAGTAAATGAACAACAAACTCCACTTCACTTGCAGCACTTACCTTAACAAAC 16700
      ----:----|----:----|----:----|----:----|----:----|----:----|----:----|----:----|----:----|----:----|

16701 ATAGTTCTTGCTCATAACAGTTCATCTCAGGATGAATCCATTTCCTTTTGGAAAAAATAATCGCTGAATATTACATTTTACTTGTTTATTTAACATTTTA 16800
      ----:----|----:----|----:----|----:----|----:----|----:----|----:----|----:----|----:----|----:----|

16801 CTATATAGAAAATTTTGACTATAAACAAAAACAGAGAGGCGTTCCCTGGTGGTCTAGGGGTTAGGTTTTGGTGCTTTCACTGCTGCAGCCTGGTTTCAAC 16900
      ----:----|----:----|----:----|----:----|----:----|----:----|----:----|----:----|----:----|----:----|

16901 CCCTGGTTGGGGAACCAAGAACCTACAAGTTGTGCAGTGTGGCCAAAAAATAAAATAAAAACAGGATAGTATAATTTCATGAGTCTATCATTCAGTCTCA 17000
```

```
      ----:----|----:----|----:----|----:----|----:----|----:----|----:----|----:----|----:----|----:----|

17001 GCAAATATATATCTCTTTAGCATAGTGATTTTGAGGTTCATTCAAATTGTAAAATGCATCAGTGATCCATTACTTTCATTGCTGAATGGCATTCCATTGG 17100
      ----:----|----:----|----:----|----:----|----:----|----:----|----:----|----:----|----:----|----:----|

17101 ATGAATACACAGTTTGTGTAGTGTATTCATTCAATAAGGAGATTTGGATTGTTACCAACACGGCAGTTATGAACAACACTACTATGAATAGTTGGGTATA 17200
      ----:----|----:----|----:----|----:----|----:----|----:----|----:----|----:----|----:----|----:----|

17201 CATCCTTGCATAGATAAATGTTCTTATTCTCTTGGGTAGATTCCTAGGAAGAAACTGCCAGACAGTTTTTCAAAGTGACTCTATCATTTCACAGTCCAAC 17300
      ----:----|----:----|----:----|----:----|----:----|----:----|----:----|----:----|----:----|----:----|

17301 CTGCAGTTTCCAGTTGCTGTATGAACATTCCAGTTTCTCTACAATCTTCCACAGGTCCATTTATACATCTTTGCTGAAAAGCTTATTCAAATCTATTAAC 17400
      ----:----|----:----|----:----|----:----|----:----|----:----|----:----|----:----|----:----|----:----|

17401 TAGAATAATAGGATTTTTAATCGGAATATTTGTTGTCTTATTTTGAGATGTTATATTACAGATACAAGTCCTTTCAGGTAGATGACATTTTCCTTCTGTC 17500
      ----:----|----:----|----:----|----:----|----:----|----:----|----:----|----:----|----:----|----:----|

17501 TGAAGGATGACCTGTAACAGATCGGCTGGTGATGGATTATTTCAACTTTTGTAGGTCTAAAAGCATCTCTTCACAGATGGCTTTTCTCAGAATGTTCTGA 17600
      ----:----|----:----|----:----|----:----|----:----|----:----|----:----|----:----|----:----|----:----|

17601 ACTTTACAGTGTATCCTTATTGATGTTTGGGGAAGGGGTGGTGGTTTCCTCCTTGTCTTGAGCCCTGATAATTACATGCTATCTTCCTTCTGAATCTTTG 17700
      ----:----|----:----|----:----|----:----|----:----|----:----|----:----|----:----|----:----|----:----|

17701 TGCACAGAAAATTGGGAAGCCACAATCAACAGCTCCAAGATCCATGTGATAAATATCAGAAATGAGTTTTCAGAAGAATTTTATGATTATTGGTGAATTT 17800
      ----:----|----:----|----:----|----:----|----:----|----:----|----:----|----:----|----:----|----:----|

17801 TATTATTACACCAAGATATAAGTATAAATCTTGGAGGCAAAAAAAATTTTATAACCTTATTAAAATGGATAACATTACAAAAAACACAGAGCAAGCTGAT 17900
      ----:----|----:----|----:----|----:----|----:----|----:----|----:----|----:----|----:----|----:----|

17901 AATAATTTAAGAGTATATAAAGAGTTAAACATATACACAAAGGTTAATCTTTTCCATTTTATCATCTATGAGGTTTTTATTTAATTTACTTTTATATTTG 18000
      ----:----|----:----|----:----|----:----|----:----|----:----|----:----|----:----|----:----|----:----|

18001 TGTTTAATATACAAAGAGATAATAAATTTAAAGCCTTTACTTTTATTTTTAAAACAATTAAAAATCTGATATTATATATATATAAGATTTCACATCTTAA 18100
      ----:----|----:----|----:----|----:----|----:----|----:----|----:----|----:----|----:----|----:----|

18101 CCCATAACATCTTTTTGCTAAACAGACAAAATGTAGTCCAATGAGATAGTTACATTCTTTTGTTCATATCAAAACTTTGAAATCCAGTGAATAATTTGTA 18200
      ----:----|----:----|----:----|----:----|----:----|----:----|----:----|----:----|----:----|----:----|

18201 CTTACTTGCATATCACAAGCTAAATGCTAAATTTTCATCAGAAATACTTACTCTGCATTTAGAATTCATAAAATTTACAGTTGAAAGAGTATATTCACAT 18300
      ----:----|----:----|----:----|----:----|----:----|----:----|----:----|----:----|----:----|----:----|

18301 ATGCAAGCTGTTCTAAGCATACTAAAAAGCTTCCCAATAACTGGATTAAATATCAGCATTTAAAGTTAAACTAAAATTCAATAAAATTAAAAATTCAGTA 18400
      ----:----|----:----|----:----|----:----|----:----|----:----|----:----|----:----|----:----|----:----|
```

```
18401 CCTTGGTGGAGCTAGGTATGTTTATGTGTTTGATAGCTGTGTTGAGTAGTGGCTACTCTAGTATACAGAGGAACTCTAGAGAGTAATTAAGTGGCTGGGG 18500
      ----:----|----:----|----:----|----:----|----:----|----:----|----:----|----:----|----:----|----:----|

18501 AACGAAGATGGCTTCAATCTTGTACTATTTCCATACACAACTTACCCAAATACATGAACAAAACATTTTCTTGTTTTAAAGAATATGGTAACTAGTAAGT 18600
      ----:----|----:----|----:----|----:----|----:----|----:----|----:----|----:----|----:----|----:----|

18601 TATAGAGATCAGATATACCCTAACAGGATGAATGCTGGGGCAAAACAGTTCTGTTAATATTATATAAGTTTCTTGTAACTTAAAACGAAATTTCTAGTAA 18700
      ----:----|----:----|----:----|----:----|----:----|----:----|----:----|----:----|----:----|----:----|

18701 TAGCATTTCACAAACACATTACAAAAGTGCTTCATCACAAAAAGATTTATTTCAAAGTAAAGATTATGTGCCTGGTAAAACTATACTATTTAATTAATAG 18800
      ----:----|----:----|----:----|----:----|----:----|----:----|----:----|----:----|----:----|----:----|

18801 GATCAGAGTGTCAATGAAGACATGGATATTTAAGATAAATGCAAACAGTTTGAATAGTTTTGTAAATTGTGAGTAGAAAAATAAATATTTATAAATTAAT 18900
      ----:----|----:----|----:----|----:----|----:----|----:----|----:----|----:----|----:----|----:----|

18901 ATTTTATATGCTGTGCTGTGCTCAGCCATGTTCGACTCTTTGCAGTTCCATGGACTGTGGACTGCCAGGCTCCTCTGTCTATGGAATTTTCCAGGCAAGA 19000
      ----:----|----:----|----:----|----:----|----:----|----:----|----:----|----:----|----:----|----:----|

19001 ATACTGGAGAGGGTTGCCATTTCCCACTCCAGATGATCTTTGCACCCCAGGGGCCAAACCCAGGTCTCCTGCATCTCCTGCATTGGAAGGTGGGCTCTTT 19100
      ----:----|----:----|----:----|----:----|----:----|----:----|----:----|----:----|----:----|----:----|

19101 ACAACTAGTGTCAACTAGGAAGCCCAATATTTTATATATTTGATTTAAAATACTTGTCTATGAACTTTAAAATTAAATTAGATACTATAAGTTTAGGCAT 19200
      ----:----|----:----|----:----|----:----|----:----|----:----|----:----|----:----|----:----|----:----|

19201 ATGTACATTTTTATATGTGATTGTGCTTCCCTGGTGGCTCAATGGAGAAAATCCACCCGCCAATTCAGGAGACTCAGCTTCCATCTCTGGGTCAGAAAGA 19300
      ----:----|----:----|----:----|----:----|----:----|----:----|----:----|----:----|----:----|----:----|

19301 TTGAAGATCCCTTGGGGAAGGAAATGGCAACCCCCTCCAGTATTCTTGCCTGGGAAATCCCATGGACAGAGGAGCCTGGTGGGCTACAATCCGTGGGGTC 19400
      ----:----|----:----|----:----|----:----|----:----|----:----|----:----|----:----|----:----|----:----|

19401 ACAGAAGAGTCAGACATGACTTAGTGACTCAACAACAACAAATATGTGATTATGTTAAATACATTTTATAAAAGGTATTTCCATGTATGGTCATAAGTGG 19500
      ----:----|----:----|----:----|----:----|----:----|----:----|----:----|----:----|----:----|----:----|

19501 TGTTAGTCTTATAGTTTTACTGATAGTGTTTTTTCTTGTCTGATTTTAGTATTGATGTTAGGAACTTCAAATACATAAACGAGGCCCAAGTGAAGATTTC 19600
      ----:----|----:----|----:----|----:----|----:----|----:----|----:----|----:----|----:----|----:----|

19601 CTCCTTGTACTGAAATGGGCATATAACAGGAGAATTAGCACAGCATTGGCTTGAATTCTATAACTTACCTATGTTTTGGGGAGGTTGTTATTAGTCTATT 19700
      ----:----|----:----|----:----|----:----|----:----|----:----|----:----|----:----|----:----|----:----|

19701 CAGTGTCTACAGCTCTTTTTCAGCAAATTTTCTTAATCATGTGTTCTTAGAAAATTTCCTTCTCTCCATTGCCATTTAAAAAAAATATTCCATAGTTTTT 19800
      ----:----|----:----|----:----|----:----|----:----|----:----|----:----|----:----|----:----|----:----|

19801 CAATTGTCTCCACATCTTTAGTTATATCTCATTTTTATGAACCTCTTCCCAGAGGAGTGAATAGAGCTTCATAAAGCCTGGAGAAGAAAAATCCCATTTT 19900
      ----:----|----:----|----:----|----:----|----:----|----:----|----:----|----:----|----:----|----:----|
```

```
19901 CCAGATCAGATAGACTAGGTTTCCAATAAACAAATAAAAAGAGGAAACCATTTTGAAGAATGTGTTATATATTCAGGTGTAGGGAACATGAGGTTCCTTC 20000
      ----:----|----:----|----:----|----:----|----:----|----:----|----:----|----:----|----:----|----:----|

20001 TCAGTTTCTAAAAGAAACAAAGTGTGGGAAAGAAGACGAGGGAGACCACTGTCCACTGCCCTGAGGGTAGGCAGGACAGACAGAACTGAGTACAGAGAAA 20100
      ----:----|----:----|----:----|----:----|----:----|----:----|----:----|----:----|----:----|----:----|

20101 ATTAAAGAGGTAACATTTCCCAACAGGAACAAACTAAATTTCACAGGACTCAGCCTCTTAGAGTCAAGACCTGGGAATTTACTTAATCTGGATTCCAAGC 20200
      ----:----|----:----|----:----|----:----|----:----|----:----|----:----|----:----|----:----|----:----|

20201 CTGGTTGAAGGGGAGAGAAGTCATGGGTAGTTCACAGCGGTGGTGTTCTGGGGGATTTGAGTGGGAAGGGAATTGCGTTATGTGACCACCTCCTCTCTAT 20300
      ----:----|----:----|----:----|----:----|----:----|----:----|----:----|----:----|----:----|----:----|

                      K  W  K  K  S  H  E  R  T  S  F  T  H  T  Q  Y  K  E  L  E  A  L  F  S  C  N  M  F      F1
                    Q  N  G  R  R  V  M  N  A  P  H  S  P  T  H  S  T  R  S  W  R  L  C  L  A  A  T  C  F     F2
                     K  M  E  E  E  S  *  T  H  L  I  H  P  H  T  V  Q  G  V  G  G  S  V  *  L  Q  H  V  S    F3
20301 CTCTTGCTCCACAGCAAAATGGAAGAAGAGTCATGAACGCACCTCATTCACCCACACACAGTACAAGGAGTTGGAGGCTCTGTTTAGCTGCAACATGTTT 20400
      ----:----|----:----|----:----|----:----|----:----|----:----|----:----|----:----|----:----|----:----|

      P  D  K  N  L  Q  R  E  L  A  L  K  L  N  L  P  E  S  T  V  K                                           F1
       Q  I  K  T  S  R  E  N  L  L  *  N  S  I  Y  Q  S  Q  Q  *  R                                          F2
        R  *  K  P  P  E  R  T  C  F  K  T  Q  S  T  R  V  N  S  K  G                                         F3
20401 CCAGATAAAAACCTCCAGAGAGAACTTGCTTTAAAACTCAATCTACCAGAGTCAACAGTAAAGGTCTGTCTGATCATCGGAGATGTGCTTTGAACCCTCT 20500
      ----:----|----:----|----:----|----:----|----:----|----:----|----:----|----:----|----:----|----:----|

20501 TTCAAATAACATTCTACTATTCAGCCTGGAAATTCCCTCCATAAAACAGTAACACTTCATTTATTGAGTCCCTGCTGTGTGCTAGTACTGAGCTGGGCAG 20600
      ----:----|----:----|----:----|----:----|----:----|----:----|----:----|----:----|----:----|----:----|

20601 CTGTATTAATACATGTTGGCTTTATTATCATCTAGTTTAAAAGATGAGAAAACTGAGGTTTGGGAGCAAAGTGACTGGGTTACAGAGATAATACATAGTA 20700
      ----:----|----:----|----:----|----:----|----:----|----:----|----:----|----:----|----:----|----:----|

20701 TCACTGGAATTGGAACTCCAGAGGCACGATAGAATTCACATTAGAGAAATGATATCATCCAGACAGACTCTCATTTAGGGTCTTCTCTAGCAAAACCCCG 20800
      ----:----|----:----|----:----|----:----|----:----|----:----|----:----|----:----|----:----|----:----|

20801 CAATCACTTATTTACAACGCTGCCAACAGACAACAACTCCAACCTTTGAGTCTGCTGAGCCCCCAAATTTTTTTTACAATATTGCTCACTGTTCAGTTCA 20900
      ----:----|----:----|----:----|----:----|----:----|----:----|----:----|----:----|----:----|----:----|

20901 GTTCAATCACTCAGTCGTGTCCAACTCTTTGTGACCCCATGGTCTGCAGCACGCCAGGCCTCCCTGTCCATCACCAACTCTTGGAGTCTACCCAAACCCA 21000
      ----:----|----:----|----:----|----:----|----:----|----:----|----:----|----:----|----:----|----:----|
```

```
21001 TGTCCGTTGAGTTGGTGATACCATCCAACCATCTCATCTTCTGTCGTCCCCTTCTACTCCTGCCTTCAATCCTTCCCAGCATCAGGGTCTTTTCCAATGA 21100
      ----:----|----:----|----:----|----:----|----:----|----:----|----:----|----:----|----:----|----:----|

21101 GTCAGCTCTTCACATCAGGTGGCCAAAGTATCGGAGTTTCAGCTTCAGCATCAGTCCTTCCAATGAACATCCAGGACTGATCTCCTTTAGGGTGGACTGG 21200
      ----:----|----:----|----:----|----:----|----:----|----:----|----:----|----:----|----:----|----:----|

21201 TTGGATCTCCTTGCAGTCCAAGGGACTCTCAAGAGTCTTCTCCAACACCACAGTTCAAAAGCATCAATTCTTCAGGCCTCAGCATTCTTTATAGTCCAAA 21300
      ----:----|----:----|----:----|----:----|----:----|----:----|----:----|----:----|----:----|----:----|

21301 TTTCACATCCATACATGACTACTGGAAAAACCATAGCCTTGACTAGACGGACCTTTGTTGGCAATGTAATGTCTCTGCTTTTTAATATGCTGTCTAGGTT 21400
      ----:----|----:----|----:----|----:----|----:----|----:----|----:----|----:----|----:----|----:----|

21401 GTTCATAACTTTTCTTGCAAGGAGCAAGCGTCTTTTAATTTCATGGCTGCAATCACCATCTGCAGTGATTTTGGAGCCCCCAAAAATAAATTCAGCCACT 21500
      ----:----|----:----|----:----|----:----|----:----|----:----|----:----|----:----|----:----|----:----|

21501 GTTCCCACTGTTTCCCCCTCTATTTGCCATGAAGTGCTGGGACCAGATGCCATGATCTTAGTTTTCTGAATGTTGAGCTTTAAGCCAAATTTTTCACTGT 21600
      ----:----|----:----|----:----|----:----|----:----|----:----|----:----|----:----|----:----|----:----|

21601 CCTCTTTCACTTTCATCAAGAGTCTCTTTAGTTCTTCTTCACTTTCTGCCATAAGGGTGGTATCATCCGCATATCTGAGGTTATTGATATTTTCCATAAT 21700
      ----:----|----:----|----:----|----:----|----:----|----:----|----:----|----:----|----:----|----:----|

                              F  G  S  G  T  G  G  S  K  *  R  S  S  S  G  S  K  S  S  N  H  *  S  H  Q  A     F1
                               L  V  Q  E  P  A  V  Q  N  E  E  A  A  A  G  A  R  A  A  I  T  K  A  T  K  P  F2
                             I  W  F  R  N  R  R  F  K  M  K  K  Q  Q  R  E  Q  E  Q  Q  S  L  K  P  P  S      F3
21701 TTCCTCTCTTCCCCCACTCCAGATTTGGTTCAGGAACCGGCGGTTCAAAATGAAGAAGCAGCAGCGGGAGCAAGAGCAGCAATCACTAAAGCCACCAAGC 21800
      ----:----|----:----|----:----|----:----|----:----|----:----|----:----|----:----|----:----|----:----|

       R  S  F  Q  P  R  M  C  P  Q  Y  Q  P  A  L  I  L  F  S  L  Q  F  Q  I  P  I  T  P  S  H  L  S  P     F1
        G  P  S  S  Q  G  C  A  H  S  I  N  Q  P  S  F  F  S  P  C  N  F  R  F  L  *  L  P  L  T  S  A  L   F2
      Q  V  L  P  A  K  D  V  P  T  V  S  T  S  P  H  S  F  L  L  A  I  S  D  S  Y  N  S  L  S  P  Q  P  L  F3
21801 CAGGTCCTTCCAGCCAAGGATGTGCCCACAGTATCAACCAGCCCTCATTCTTTTCTCCTTGCAATTTCAGATTCCTATAACTCCCTCTCACCTCAGCCCT 21900
      ----:----|----:----|----:----|----:----|----:----|----:----|----:----|----:----|----:----|----:----|

      *  T  L  S  P  G  Q  G  T  L  *  S  W  R  F  L  Q  V  M  S  K  R  K  I  L  N  W  R  G  *  W  P  Q  F  F1
       R  H  F  P  L  G  R  G  L  Y  D  H  G  D  S  Y  K  *  C  P  N  A  R  S  S  T  G  E  A  S  G  L  S    F2
        D  T  F  P  W  A  G  D  S  M  I  M  E  I  P  T  S  D  V  Q  T  Q  D  P  Q  L  E  R  L  V  A  S  V   F3
21901 TAGACACTTTCCCCTGGGCAGGGGACTCTATGATCATGGAGATTCCTACAAGTGATGTCCAAACGCAAGATCCTCAACTGGAGAGGCTAGTGGCCTCAGT 22000
      ----:----|----:----|----:----|----:----|----:----|----:----|----:----|----:----|----:----|----:----|

        L  L  C  T  L  M  H  L  T  S  P  K  S  W  N  C  T  V  F  L  M  R  M  T  S  P  T  L  P  S  I  L  Y   F1
      S  C  F  V  L  *  C  I  *  H  H  P  N  H  G  T  V  Q  C  S  *  *  G  *  H  H  Q  L  F  L  L  F  S  I  F2
```

```
      P  A  L  Y  S  D  A  F  D  I  T  Q  I  M  E  L  Y  S  V  P  D  E  D  D  I  T  N  S  S  F  Y  S  L      F3
22001 TCCTGCTTTGTACTCTGATGCATTTGACATCACCCAAATCATGGAACTGTACAGTGTTCCTGATGAGGATGACATCACCAACTCTTCCTTCTATTCTCTA 22100
      ----:----|----:----|----:----|----:----|----:----|----:----|----:----|----:----|----:----|----:----|

      I  S  I  S  H  R  Q  G  P  V  R  I  E  F  F  S  Y  S  L  C  *  S  S  C  R  L  I  S  W  A  D  L  L      F1
       S  V  S  L  T  D  K  A  Q  L  E  *  S  S  S  L  I  V  F  A  D  P  A  V  G  L  F  P  G  Q  T  S  F     F2
     Y  Q  Y  L  S  P  T  R  P  S  *  N  R  V  L  L  L  *  S  L  L  I  Q  L  *  A  Y  F  L  G  R  P  P  S    F3
22101 TATCAGTATCTCTCACCGACAAGGCCCAGTTAGAATAGAGTTCTTCTCTTATAGTCTTTGCTGATCCAGCTGTAGGCTTATTTCCTGGGCAGACCTCCTT 22200
      ----:----|----:----|----:----|----:----|----:----|----:----|----:----|----:----|----:----|----:----|

     Q  C  D  K  L  E  L  C  S  L  Q  S  T  G  Q  P  G  I  P  E  P  L  H  Y  S  A  L  W  I  S  L  I  Q  V    F1
      S  V  T  S  W  S  F  A  V  Y  S  P  Q  D  S  L  E  F  Q  N  P  S  I  T  V  H  F  G  F  L  *  S  K     F2
       V  *  Q  A  G  A  L  Q  S  T  V  H  R  T  A  W  N  S  R  T  P  P  L  Q  C  T  L  D  F  S  D  P  S    F3
22201 CAGTGTGACAAGCTGGAGCTTTGCAGTCTACAGTCCACAGGACAGCCTGGAATTCCAGAACCCCTCCATTACAGTGCACTTTGGATTTCTCTGATCCAAG 22300
      ----:----|----:----|----:----|----:----|----:----|----:----|----:----|----:----|----:----|----:----|
```

Cloning primer

```
       L  I  N  I  R  Q  Y  R  K  R  I                                                                     F1
     Y  *  *  I  S  D  S  I  E  K  G                                                                       F2
      T  N  K  Y  Q  T  V  *  K  K  D                                                                      F3
22301 TACTAATAAATATCAGACAGTATAGAAAAAGGA                                                                   22400
      ----:----|----:----|----:----|---
```
