# Supplementary material for: Molecular cloning of PRD-like homeobox genes expressed in bovine oocytes and early IVF embryos
Source: BMC Genomics. 2024 Nov 6;25:1048. doi: 10.1186/s12864-024-10969-w (PMC11542365; doi:10.1186/s12864-024-10969-w)
Supplement: Supplementary file 5 — Supplementary Material 5: Additional file 11: Figure S3. The prediction of DUXA derived from Bos taurus isolate L1 Dominette 01449 registration number 42190680 breed Hereford chromosome 18, ARS-UCD1.2, whole genome shotgun sequence. Three possible ORFs for exons, but not introns, are depicted. Putative protein sequence is highlighted in yellow. Sequences from StringTie merge prediction and confirmed cDNA are drawn as lines below the corresponding sequences. Cloning primers are drawn as line arrows. Splice sites are underlined and codons split by two exons are coloured red. The homeodomains are highlighted in green. [file 12864_2024_10969_MOESM5_ESM.pdf]

Supplementary Figure S3. The prediction of *DUXA* derived from *Bos taurus* isolate L1 Dominette 01449 registration number 42190680 breed Hereford chromosome 18, ARS-UCD1.2, whole genome shotgun sequence. Three possible ORFs for exons, but not introns, are depicted. Putative protein sequence is highlighted in yellow. Sequences from StringTie merge prediction and confirmed cDNA are drawn as lines below the corresponding sequences. Cloning primers are drawn as line arrows. Splice sites are underlined and codons split by two exons are coloured red. The homeodomains are highlighted in green.

```
         A  H  I  K  A  N  *  L  V  L  L  V  C  L  L  L  E  T  A  F  G  A  P  L  Q  T  G  Q  D  G  P  S  D    F1
       L  L  I  *  K  P  T  S  W  C  C  L  S  A  F  C  W  K  Q  P  L  E  L  L  C  R  R  V  K  T  V  L  Q  T   F2
        C  S  Y  K  S  Q  L  A  G  A  A  C  L  P  S  V  G  N  S  L  W  S  S  S  A  D  G  S  R  R  S  F  R     F3
10001 CTGCTCATATAAAAGCCAACTAGCTGGTGCTGCTTGTCTGCCTTCTGTTGGAAACAGCCTTTGGAGCTCCTCTGCAGACGGGTCAAGACGGTCCTTCAGA 10100
      ----:----|----:----|----:----|----:----|----:----|----:----|----:----|----:----|----:----|----:----|
                                                                               Cloning primer 1
StringTie merge
                                                                               DUXA_16c_01

        S  K  F                                                                                                F1
         V  S  S                                                                                               F2
       Q  *  V  P                                                                                              F3
10101 CAGTAAGTTCCTCACGCTTCAGACCGCTGTGCTCTTTTCCTTGGTTGGGGGGAGAGGGAGGGGAATTGGACTGAAAACATACTGAGCTTATGAGAAGGTA 10200
      ----:----|----:----|----:----|----:----|----:----|----:----|----:----|----:----|----:----|----:----|

10201 TGGGAGTCCCATTTTTCAAGCGTTTAAATAACTGTGCTAGGTAAGAGAAAACTGATCCCTCCTGTAATATGAAAGCATGCTGTTTTCTCTGCATCTGAGC 10300
      ----:----|----:----|----:----|----:----|----:----|----:----|----:----|----:----|----:----|----:----|

10301 ACTGGGAATTTCAAAAAGATGGAGGCACGCAAGAACCAGACAATACAGGAGGAGGGTGAAAACAACTGAACAATACAAAACGTTGGTTCAAGAAGGGAGA 10400
      ----:----|----:----|----:----|----:----|----:----|----:----|----:----|----:----|----:----|----:----|

10401 TGGAAGGGAAAAATTGATGGAAGAAGGAAGAGGAGCTATGTGCTGTGCTAAGTGGCTTCAGTCGTGTCCAACTATTTGCAGCCACCTGGGCTGTAGCTCG 10500
      ----:----|----:----|----:----|----:----|----:----|----:----|----:----|----:----|----:----|----:----|

10501 CCAGGCTCCTCTGTCCCTGGGATTCTCCAGGCAAGAACACTGGAGCGGGTTGCCGTTTTCTTCTCCAGGGGATCTCCCCGCCCAGGGATGGAACCCACAT 10600
      ----:----|----:----|----:----|----:----|----:----|----:----|----:----|----:----|----:----|----:----|

10601 CTCTTATCGGCAGCCAGGTTCTTTACCGTTAGTGCCACCGGGGAAACCCAAGGAAATGATGGGGGTCATCAAAAAGCCCTGAGACCAGTAAGCTTTTGGA 10700
      ----:----|----:----|----:----|----:----|----:----|----:----|----:----|----:----|----:----|----:----|

10701 ATCGAAGTAGCTTTCAGATTTCTCTCTCCAGACTGAACTTGTCTCCTGAACTTCAGCTTTGTGCATCTAACTGCCTGTAATAGGTGTCTCAAAATCAACG 10800
      ----:----|----:----|----:----|----:----|----:----|----:----|----:----|----:----|----:----|----:----|

10801 TGTCCAAAATGAAACTCCTTATGTTCAGACCCAAATTCTTCCTCCTGTTCTGCGTGCTCACCAAATATCAACTGTTTGTGCCCCAAACCTAAGCGTTCTT 10900
      ----:----|----:----|----:----|----:----|----:----|----:----|----:----|----:----|----:----|----:----|
```

```
10901 GGCCTTGTCTTTCTCTCATAGGGCTTCCCTGGTGGCTCAGATGGTAAGGAATTTGCCTGCAATGCAGGAGACCTGGGTTTGATCCCTGGGTAGGGAAGAT 11000
      ----:----|----:----|----:----|----:----|----:----|----:----|----:----|----:----|----:----|----:----|

11001 CCCCTGGAGGAAGGAATGGCTACCCACTCCAGTGTTCTTGACTGGGGAATTCATAAGCTACACCTAACCAGGCTGAGAATCCTGTTGGCTTTAACAAAAC 11100
      ----:----|----:----|----:----|----:----|----:----|----:----|----:----|----:----|----:----|----:----|

11101 ACTTAAACCATCATTGCTGGATTATGGCAAGAATTGATCTCTCTGCCCCCATCCTGGCCCTGATTCAATCTATTTTCAACTCTGTAGCCTGTGTGATCTT 11200
      ----:----|----:----|----:----|----:----|----:----|----:----|----:----|----:----|----:----|----:----|

11201 GTCAAACTGTCTGATCAACTCTTCTCACTATTGGCCAGCCAAGATTGCTCAGTCGTGTCCCACTCTGAGACCCCATGACTGTAGGCCTGCCAGCTCCTCT 11300
      ----:----|----:----|----:----|----:----|----:----|----:----|----:----|----:----|----:----|----:----|

11301 GTCCATGGGATTTCCCAGGCAAGAATACTGGAGTGGGTCGCCATTCCTTCTCCATGGGATCCTCCTGACCCAGGGATTGCACCTGGGTCTACCTGGGAAG 11400
      ----:----|----:----|----:----|----:----|----:----|----:----|----:----|----:----|----:----|----:----|

11401 CCCCACCATACTAGAGTCAAAGGCACATGGCCTCAAGAGGCCCCATAGAATCTGCCCACTTGGAGGTCTAAGACCTCATCTGCTTCTGGTTCATTTTCTC 11500
      ----:----|----:----|----:----|----:----|----:----|----:----|----:----|----:----|----:----|----:----|

11501 TTCTCCAACCCTCCTGCTCTACCGAGTTTATTTCTCTGTCATTTTTTCCCTCATTACTTTGTCTTGTGTGTAAAATTCCATTACGGGAATATACCAGGTT 11600
      ----:----|----:----|----:----|----:----|----:----|----:----|----:----|----:----|----:----|----:----|

11601 TTACTTATCCATTCTCCTAGCAATGGGTGTTCAGTTGCTTCAGCTTACTACAAAATACCATTCTGAAAACTGTCATGGAGATGTATTTGGGGTTAGAATT 11700
      ----:----|----:----|----:----|----:----|----:----|----:----|----:----|----:----|----:----|----:----|

11701 ACAAATTATTCTTTGTAATCTGTTACAAAGAAATGGAGTTGGGGTTAAAACTACCTACTGTTGTTGTTTAGCCACTCAATTGTGTCCGACTCTTTGCGAC 11800
      ----:----|----:----|----:----|----:----|----:----|----:----|----:----|----:----|----:----|----:----|

11801 CCTATGGACCACCAGGCTCCTCTGCCTGTGGAATTTTTCAGGCAAGAAATACTGGAGAGGGTTGCCATTTCCTTCTCCAGGGGATCTTCTTGACCCTGGG 11900
      ----:----|----:----|----:----|----:----|----:----|----:----|----:----|----:----|----:----|----:----|

11901 ATGGAGCCCTTGTCTCTTGCGTCTCCTGCATTGGCAGGCGGGTTCTTTACCACTAGCGCCACCTGGGAGAGCAGTGTGGAGGCTCCTTAAAAAAGAAAAA 12000
      ----:----|----:----|----:----|----:----|----:----|----:----|----:----|----:----|----:----|----:----|

12001 TAGAACCACGATACGATCCAGCGTGTTCTTTTCTGTGAGGTGTCAACCAAAGCAAATCCCTAAGTTTCCTCTTTGTATGAGTCCAGCTCCAAACGAACCT 12100
      ----:----|----:----|----:----|----:----|----:----|----:----|----:----|----:----|----:----|----:----|

12101 CTAGCATGCACAGTAACTTTTCTTTTCATCTTATTTAGTTTACCAAGCCTAATTCATTCATGTTCCAAAATGAACGCCCAGGTTTTCCAGATTTGGCTAT 12200
      ----:----|----:----|----:----|----:----|----:----|----:----|----:----|----:----|----:----|----:----|

12201 TTTAATGAGCTGTATCATATCTAAAATCATGCAACATGGCATGTATTCGTTATTTGAAGATTCTACTGAATGTACGAATTGTGTTCACCCCATGGTGTTT 12300
      ----:----|----:----|----:----|----:----|----:----|----:----|----:----|----:----|----:----|----:----|

12301 AAAAAAATATTTGTAGGGGGATTTCCCTGATGGTCCGGCAGGTAAGGATCGGTGCTTCCGTTGCAGGGGGCACAGGTTCAAGCACTCGTTGGGGAACTAA 12400
```

```
      ----:----|----:----|----:----|----:----|----:----|----:----|----:----|----:----|----:----|----:----|

12401 GATCCTGCAGGCTATGCAGTCAGGCAAAAAAAAATGGGAACTGATCCTATTTGTTGAGGGCAATGAACTTCAAAATGAGTCAATCTGTACTAGGAATTTA 12500
      ----:----|----:----|----:----|----:----|----:----|----:----|----:----|----:----|----:----|----:----|

12501 ACCAGAAAACCTGAAAGGAGCTCAACAATACCTGTAACAGGTGCTCTTTGTGAGTTCAAATGCCCATCATTGTCATGACATCACCTGCGTCATCCTGACT 12600
      ----:----|----:----|----:----|----:----|----:----|----:----|----:----|----:----|----:----|----:----|

12601 TGCATTGTTCTTATCAAGAACAATGTCCCATAGGACTGGGACCCATCAGTTTTTCATGTTTTTTTTTTTTTTTCCTTTTTTCTTTTTTTAAACAGAAGACA 12700
      ----:----|----:----|----:----|----:----|----:----|----:----|----:----|----:----|----:----|----:----|

12701 TATGTCCCTAGTCATGACCTTCAGGTCAACTAAGGAAGCTTCAAGAATCACCACCCTTGTGCACTTAAGGGCTTTATAATCGAGGGTTCTTTCCATCTGG 12800
      ----:----|----:----|----:----|----:----|----:----|----:----|----:----|----:----|----:----|----:----|

                                                                                                              F1
                                                                                                         F    F2
                                                                                                          L   F3
12801 GTTCTGGTGGATTCAGAATGAAAAGAGGAAATGAAAGACAAGCTCTAAGCTGAAGCGTGAATACATACGATGGCCCATTTAATGCTTCTCTCCTCCGATT 12900
      ----:----|----:----|----:----|----:----|----:----|----:----|----:----|----:----|----:----|----:----|
                                                                                   Cloning primer 2
                                                                                   DUXA_16c_02

       Y  W  S  L  S  P  Q  R  P  W  R  H  S  V  G  A  A  V  P  R  S  Q  K  S  S  *  R  S  S  S  R  H  S  A    F1
        I  G  L  S  L  P  R  D  H  G  D  T  V  *  A  Q  P  Y  L  V  H  R  R  A  V  E  D  P  R  P  G  I  Q     F2
         L  V  S  L  S  P  E  T  M  A  T  Q  C  R  R  S  R  T  S  F  T  E  E  Q  L  K  I  L  V  Q  A  F  S   F3
12901 TATTGGTCTCTCTCTCCCCAGAGACCATGGCGACACAGTGTAGGCGCAGCCGTACCTCGTTCACAGAAGAGCAGTTGAAGATCCTCGTCCAGGCATTCAG 13000
      ----:----|----:----|----:----|----:----|----:----|----:----|----:----|----:----|----:----|----:----|
Cloning primer 1

Cloning primer 2

        K  T  L  T  L  A  I  L  P  N  R  D  L  L  W  K  S  T  L  K  S  L  E  F                            F1
      P  K  P  L  P  W  L  Y  C  Q  T  E  T  C  C  G  N  Q  R  *  R  V  *  N  S                             F2
       Q  N  P  Y  P  G  Y  T  A  K  Q  R  L  A  V  E  I  N  A  E  E  S  R  I  Q                            F3
13001 CCAAAACCCTTACCCTGGCTATACTGCCAAACAGAGACTTGCTGTGGAAATCAACGCTGAAGAGTCTAGAATTCAGGTACATTCCAAGTTCCAGATCTCC 13100
      ----:----|----:----|----:----|----:----|----:----|----:----|----:----|----:----|----:----|----:----|

13101 AGGACGAGAAGCAGAAACTCAGGGTGGGAATGGGGGGGTGGGGACTTATCTGGTGGCCCAGTAGCTAAGACTCCGAGCTAGATGCAGCGAGACAGAGCTA 13200
      ----:----|----:----|----:----|----:----|----:----|----:----|----:----|----:----|----:----|----:----|

13201 CAGGAGTTTGTGTCGACTTAAAAAACAGTCACAACCGAAAAGCTGAGAGTTAGGTTTTTACTCAGTGGAAATTTTTAGGACTCCAAGCCCGGGAGGCAGC 13300
      ----:----|----:----|----:----|----:----|----:----|----:----|----:----|----:----|----:----|----:----|

13301 ATCTCAAGTAACCCTGAGAGAATTGCTCCAAAGAGGCTATAGGGGAAGAGGGAGGAGTCTGGTTATATTTGTGCTCAGTCCCGTCCGACTCTTTGTGAGA 13400
```

```
      ----:----|----:----|----:----|----:----|----:----|----:----|----:----|----:----|----:----|----:----|

13401 ATACTGGAGTGGGTTGCCATTTCCTTCTCCAGGGGCTCTTCCAGACTCAGGGATTGAACCCACAACTCCTTTGTCTCCTGCATGGGCCAGCTGATTCGTT 13500
      ----:----|----:----|----:----|----:----|----:----|----:----|----:----|----:----|----:----|----:----|

13501 ACCAGTAGCATCGCCTAGGAAGCCCCATGTTATAAAGAAGTTGCAACAAAGGGCGAGTAGTCTGAACATCCAAAGGATTTTTGTGATTTAAAAAAAAACA 13600
      ----:----|----:----|----:----|----:----|----:----|----:----|----:----|----:----|----:----|----:----|

13601 GATATCCCAAGTGAAGGAATTTGGCGTTTTTCTATGTATGAGAACGTGCAAGAGTCTGGGCTCACTGAAACCATTCCTTTCATACGCACCTCAGCTGTGT 13700
      ----:----|----:----|----:----|----:----|----:----|----:----|----:----|----:----|----:----|----:----|

13701 GACCAATATCCTGTGTTTTTTCACATCCTGAGCTCCCCTGGGGCTCATCCTAAGGAGTGGCTGGGATCTGATGGCTGTTAGATTCCAGGTATTTTCTCCT 13800
      ----:----|----:----|----:----|----:----|----:----|----:----|----:----|----:----|----:----|----:----|

13801 TTCTGAGCACCCTGGAGGGCTGGAATCTCTGATGAATGTGACATCCTTCTTTACTGATATGGCAGGAGATACTCCATTTGCATAATTTTTTGAGAATGCA 13900
      ----:----|----:----|----:----|----:----|----:----|----:----|----:----|----:----|----:----|----:----|

13901 CACCTCAAAATTCAAGGAGAGTGAAAATTTTTTTCCCTCAAAATTTTAGTAATCTCAAAAGTTATTAACTGAAATTGGTACAATGTTTCTGGAGGACAAT 14000
      ----:----|----:----|----:----|----:----|----:----|----:----|----:----|----:----|----:----|----:----|

14001 CTGTCAATATGCATTAAACCTGAATGTTCATGACATTAGACAGCATATCATTTTAAGGATTTTTTTTTTTTTTTTGCCAAGAAGATAACTTATGGTGTTT 14100
      ----:----|----:----|----:----|----:----|----:----|----:----|----:----|----:----|----:----|----:----|

14101 GCAATAATGTAATTGCGAGAATATTCACAGCATTTTGTGCTTCTTCTTTTTTAATTAAGTTTTTGGCCATGCTGCCTGGCATGCAGGATCTTGGTTCCCC 14200
      ----:----|----:----|----:----|----:----|----:----|----:----|----:----|----:----|----:----|----:----|

14201 AACCAGGGATCAAACCTGGGCTCCCTGCAGTGGAAGTGCAGAGTCTTAACCACTGGATCACCAGGGAAGTCTTAGCATTTTGTGACTCTAATAGAGAGCT 14300
      ----:----|----:----|----:----|----:----|----:----|----:----|----:----|----:----|----:----|----:----|

14301 GTTGGAGAGAAACCTCTATTCAAGAACAAGAGGAGGGACTTCCCTGGTGGTCCAGTGAATAGGAATCCATGTTCCCGATGCAGGGGCCTTGGGTTTGGCC 14400
      ----:----|----:----|----:----|----:----|----:----|----:----|----:----|----:----|----:----|----:----|

14401 CCCAGTCAGGGAACTAGACTCCACGTGCCACCACGAAAGATCCCAAGTGCCACAACCAAGACCTGGCACAGTCAAGGAAACCAAAAGTAAAATACGAAAA 14500
      ----:----|----:----|----:----|----:----|----:----|----:----|----:----|----:----|----:----|----:----|

14501 AAGAAAGGCGGTGGGGCCACTCGGTGAACACGTACCATGAGCCTGTGTGATAAGGGCGTTGAGTGCAGTGCTTTGCTTGACCCTCAGAATGAACTCCCAA 14600
      ----:----|----:----|----:----|----:----|----:----|----:----|----:----|----:----|----:----|----:----|

14601 GAATGTTGCTCATCACCTCCCTTCCAAATGTGGACACTGAGAGTGAAGGAGCTTCCAGGAGTGGGCAAGAAATGTGCAGATACACCTTCAGAAAAGAGGA 14700
      ----:----|----:----|----:----|----:----|----:----|----:----|----:----|----:----|----:----|----:----|

14701 ATGGTGTGGAAGACACACTTATTCCAGGGTGTAGAAGCCAATCAGGTCATCTAGGTGATGTGGTCACAGATAGCAGCAAGTGTCCGATGACGTGGTAAGA 14800
      ----:----|----:----|----:----|----:----|----:----|----:----|----:----|----:----|----:----|----:----|
```

```
14801 TGCCAGAGGAATGACGGATGGTGTGAGGGCTAACCTTCTCCTTTCAATGAATTCTGGCAGACCAGGGAATAGCAGACTGGTGTGTGTGCGCTCAGTTGTG 14900
      ----:----|----:----|----:----|----:----|----:----|----:----|----:----|----:----|----:----|----:----|

14901 TGACTCTTTGCAACCCCATGGACTGTAGCCCTCCAGGCTCCTCTCTCTATGGGATTTCTCAGGCAAGAATACTGGAGTGGGTTGTCATTCCCTTCTCTAG 15000
      ----:----|----:----|----:----|----:----|----:----|----:----|----:----|----:----|----:----|----:----|

15001 GGGATCTTGCCAACCTAGAGATGGAACCCACGTCTCCTGGATTGGCAGGTAGATTCTTCACCACTGTACCACCTGGGAAGCCCAGGTGTAATGTTGCAGA 15100
      ----:----|----:----|----:----|----:----|----:----|----:----|----:----|----:----|----:----|----:----|

15101 TTCAGGCTGGACGGAGATCTTACATGCAGAATAATTACAGCATCCATTTATTAACTTAAAGAGTATTACAGGGGACTTCCCTGCTGGTGCAGTGGGTAAG 15200
      ----:----|----:----|----:----|----:----|----:----|----:----|----:----|----:----|----:----|----:----|

15201 GCATTGCCTTCCAATGAAGGAATGCAGGTTTAACTGCTCACTGGGGAGCTAGGATCCCACGTGCCTCATGGCCAAAAACCAGAAGGGAAACAACAGATGC 15300
      ----:----|----:----|----:----|----:----|----:----|----:----|----:----|----:----|----:----|----:----|

15301 AATATTGTAACAAATTCAATGCAGACTTTAAAAATGGTCCATATAAAAAAATCTTTTAAAAAAGAGATTATTATAGCAAATTGTGTTAGGACCAGGTCAT 15400
      ----:----|----:----|----:----|----:----|----:----|----:----|----:----|----:----|----:----|----:----|

15401 GAAGATGCTGAATGCCAAGCCTTTATCTAAGGCTTAGTTGAATGTAACCAGGATCAGTGATAACCCTGAGTTTAGGGTTCATAAGAATCACTGCCCACCC 15500
      ----:----|----:----|----:----|----:----|----:----|----:----|----:----|----:----|----:----|----:----|

15501 CCTTTTGGGGGCCATGTCATGGCACGTGGGATTTCAGTTCCCTGACCAGGGATTGAGCTCATGCTACCTGCATGTACTAACCACTGGACCACCAGGGCAG 15600
      ----:----|----:----|----:----|----:----|----:----|----:----|----:----|----:----|----:----|----:----|

                                                                                                 I  W  F  Q     F1
                                                                                                  Y  G  F       F2
                                                                                                   M  V  S      F3
15601 TCCCCGAACCACTCCCTTTGGAGAAGAGCAGCAACGGAGCCGAAACGACATAAACATGTATAATTCTCTCGTTTTCTTTTCCTCTCTTAGATATGGTTTC 15700
      ----:----|----:----|----:----|----:----|----:----|----:----|----:----|----:----|----:----|----:----|

        N  R  R  A  R  H  G  F  L  K  K  P  K  K  N  V  D  P  S  K  D  H  A  S  P  I  E  K  I  Q        F1
      R  I  E  E  R  G  M  D  S  *  R  N  P  R  R  T  *  T  Q  A  K  T  T  P  P  P  *  R  R  F  K       F2
       E  S  K  S  E  A  W  I  P  E  E  T  Q  E  E  R  R  P  K  Q  R  P  R  L  P  H  R  E  D  S  R      F3
15701 AGAATCGAAGAGCGAGGCATGGATTCCTGAAGAAACCCAAGAAGAACGTAGACCCAAGCAAAGACCACGCCTCCCCCATAGAGAAGATTCAAGGTAAACA 15800
      ----:----|----:----|----:----|----:----|----:----|----:----|----:----|----:----|----:----|----:----|

15801 GCCTGCTCCAGCCCTGCTCCCCACCAGAGCCTCCTGGGATGCAAGTGCCTCTTGAGATTGGGGCCACCTAAAAGAAACCACCTCTTCCTTTCTCCGCCTT 15900
      ----:----|----:----|----:----|----:----|----:----|----:----|----:----|----:----|----:----|----:----|

15901 GTAAGAGGAGAGTGGGAAAGGAATGTTTCCTCCTCCGGGGTTTCCTGGTCAAGGCATCCCAGGAGCAAAGGATTCAATGAGGCCCCTGGAAAGTCTCCCC 16000
      ----:----|----:----|----:----|----:----|----:----|----:----|----:----|----:----|----:----|----:----|

16001 ACAAGGTCCAGATATTCTGAGTCTTAGCTTATTTGCGCTGGAGTCTAGCAATTTAGCCAAATACTACTGCTTCTCACAAGAGATGCGGGTTCAAGGCCTG 16100
      ----:----|----:----|----:----|----:----|----:----|----:----|----:----|----:----|----:----|----:----|
```

```
16101 GGTCAGGATGATCCCCTGGAGGAGGAAATGGCAACCCACCCCAGTAATCCCATGGACAGAAGAGCCTGGCGTCCTACAGTCCGTGGAGAGTAGGACGCGA 16200
      ----:----|----:----|----:----|----:----|----:----|----:----|----:----|----:----|----:----|----:----|

16201 CTGAGCACACCCTTCTAGAACCTTCCACCTTGAATCACCTCAAGGAGTGGCAACAGATTCCAAGTTAGATGGTGAGAACATGTTTGGGTTCCTCATCTGA 16300
      ----:----|----:----|----:----|----:----|----:----|----:----|----:----|----:----|----:----|----:----|

        M  *  A  D  P  K  C  F  S  T  F  N  P  T  P  T  P  D  G  T  D  R  R  C  R  T  S  F  T  S  S  Q  L    F1
      I  C  K  L  I  P  N  V  S  L  P  S  I  L  P  L  L  Q  M  E  Q  T  D  G  V  V  P  A  S  L  P  L  S  Y   F2
       Y  V  S  *  S  Q  M  F  L  Y  L  Q  S  Y  P  Y  S  R  W  N  R  Q  T  V  S  Y  Q  L  H  F  L  S  V     F3
16301 ATATGTAAGCTGATCCCAAATGTTTCTCTACCTTCAATCCTACCCCTACTCCAGATGGAACAGACAGACGGTGTCGTACCAGCTTCACTTCCTCTCAGTT 16400
      ----:----|----:----|----:----|----:----|----:----|----:----|----:----|----:----|----:----|----:----|

       Q  T  L  K  N  A  F  T  E  N  P  Y  P  G  I  D  S  R  E  Q  L  A  E  E  I  G  V  P  E  S  R  V  Q    F1
        K  L  L  R  M  H  S  Q  K  T  L  I  L  G  L  T  P  E  S  N  L  L  K  K  L  G  F  Q  S  Q  E  S  R   F2
      T  N  S  *  E  C  I  H  R  K  P  L  S  W  D  *  L  Q  R  A  T  C  *  R  N  W  G  S  R  V  K  S  P  G  F3
16401 ACAAACTCTTAAGAATGCATTCACAGAAAACCCTTATCCTGGGATTGACTCCAGAGAGCAACTTGCTGAAGAAATTGGGGTTCCAGAGTCAAGAGTCCAG 16500
      ----:----|----:----|----:----|----:----|----:----|----:----|----:----|----:----|----:----|----:----|

16501 GTAATCGAAAAGGGACTTGATATTGTGTGTTCATAATGAGACATCTTTAAATGATTTCTCCATCAGGGACTTATTCTCATAGACATCATAAGCAGGACAG 16600
      ----:----|----:----|----:----|----:----|----:----|----:----|----:----|----:----|----:----|----:----|

16601 ATGAAGACTCCCTGGTGGTTCAGTGGCTAAGACTTCATACTCCCAATACCTGGGGTGCAGTTCAATTCCTGGTCAAGGAACTAGATCCCACATGCCACAG 16700
      ----:----|----:----|----:----|----:----|----:----|----:----|----:----|----:----|----:----|----:----|

16701 GCATGCTACAACTATAAAAATCCCAAGCAAGTGCTGCAACTAAGATCTGGTGCAGCCAAATAAATAAATATTAAGAAAAACAAATAGCATAATATGTTAT 16800
      ----:----|----:----|----:----|----:----|----:----|----:----|----:----|----:----|----:----|----:----|

16801 AAATGAAAAAAAGATAAGCAAGACAGATGAAAAATGGGATTTCTGGCTTGAATAGTTCTTCCCCAAGGGCTCAGTAGGTGAAGAATGCAATGCAGGAGAC 16900
      ----:----|----:----|----:----|----:----|----:----|----:----|----:----|----:----|----:----|----:----|

16901 ACTGAAGACTCATGTTCAATCCCTGGGTGGGGAGGATCCCGTGGAGTAGAAAATGGCAACTCACTTCAGTATTCTTGCCTGGAAAATCCCATGCACAGAG 17000
      ----:----|----:----|----:----|----:----|----:----|----:----|----:----|----:----|----:----|----:----|

17001 AAGCCTGGTGGGCTACAGTCCATGGGGGTTCTGAAGAGTTGGATATGACTGAGTATGCATGCACCCATGGGATAACAATGTCTTCCTAGAAGACATTACC 17100
      ----:----|----:----|----:----|----:----|----:----|----:----|----:----|----:----|----:----|----:----|

17101 CTATTCTGGGTCATAGAGTAAAATCAGGCATTTTTGAGAGGAAGGAGAGAAGTGGGAAGAGAATTCCAGGCAGAGAGAAAAGCTGAAATACAGGTATGGC 17200
      ----:----|----:----|----:----|----:----|----:----|----:----|----:----|----:----|----:----|----:----|

17201 CCTTTAGGAAAGACTAATACTTACAATGAGGGAATTGCGGGGACTTTCAGGATATGAAACTTATCAGTAAATCATAAAGGTGTTTGTACAAGATTCAGAA 17300
      ----:----|----:----|----:----|----:----|----:----|----:----|----:----|----:----|----:----|----:----|

17301 TTTTGAGGGACATCTCTGGTGGCCCAGTAGTTAGGACTCCACGCTTCCACTTCAGGGACACGGGTTTGATCCCTGATTGGGGAACTAAGATCCCTGCAAG 17400
```

```
      ----:----|----:----|----:----|----:----|----:----|----:----|----:----|----:----|----:----|----:----|

17401 CCACAGACACAGCAACAACCCCCCCAAAAAAATTTCAGAATTTTGAACTTTATCTTGAAATGTGAGACTTTCCCAGATGTCATATGGAATGTCCGTGGTT 17500
      ----:----|----:----|----:----|----:----|----:----|----:----|----:----|----:----|----:----|----:----|

17501 TGAGAATTATGCCTAAAAGAAACCATCTCTTCCTCACCTTTGCCTTATAAGATGAGCGATGGTGAGACTTGCATGACATCAATTTTTTTTTCATTAAATC 17600
      ----:----|----:----|----:----|----:----|----:----|----:----|----:----|----:----|----:----|----:----|

       L  C  F  T  Y  L  D  L  V  P  K  P  K  S  Q  T  P  C  P  E  K  K  R  P  *  R  S  S  I  P  K  T  K     F1
        F  V  L  P  T  *  I  W  F  Q  N  R  R  A  R  L  R  V  Q  R  K  K  R  G  L  E  E  A  L  Y  Q  R  Q  N    F2
      S  L  F  Y  L  L  R  F  G  S  K  T  E  E  P  D  S  V  S  R  E  K  E  A  L  K  K  L  Y  T  K  D  K  T     F3
17601 TCTTTGTTTTACCTACTTAGATTTGGTTCCAAAACCGAAGAGCCAGACTCCGTGTCCAGAGAAAAAGAGGCCTTGAAGAAGCTCTATACCAAAGACAAAA 17700
      ----:----|----:----|----:----|----:----|----:----|----:----|----:----|----:----|----:----|----:----|

      P  G  T  G  S  L  I  W  E  S  *                                                                       F1
       Q  G  Q  D  L  *  Y  G  R  V  E                                                                      F2
        R  D  R  I  S  D  M  G  E  L  K                                                                     F3
17701 CCAGGGACAGGATCTCTGATATGGGAGAGTTGAAGGTACTAGCCTGCTGTTCCCAATGTGACTCACAATCAGAGTCTCCTGAGTGCCAGTTATTCCGCAC 17800
      ----:----|----:----|----:----|----:----|----:----|----:----|----:----|----:----|----:----|----:----|

17801 GGCGGACTCAAGTCCAGCTTCCTCTGGGTAGTGGATGATGCTTCCACTCAAGTGTGCCCCCTGGGGTTGGGGGTGGTCACAGTCCCCACGGCTCTCCCCA 17900
      ----:----|----:----|----:----|----:----|----:----|----:----|----:----|----:----|----:----|----:----|

17901 AGTCTGTGGTCCTCCCGAGGAATTCCACTGCCCGCTTTTCAAGCTCTGAACTGGAACCCAGAAACCCTGTTAGTATGTGAGCTTTGTTTATTAATATTTT 18000
      ----:----|----:----|----:----|----:----|----:----|----:----|----:----|----:----|----:----|----:----|

18001 CCCTAGTTCTGGCACATGGTAAACAATCAGATTTTGTGGAAGGTATGTCTGAGTTACTGGCAGGATGAATCCTCACGTGTGTTCTGCCAGGGGCTGGCTC 18100
      ----:----|----:----|----:----|----:----|----:----|----:----|----:----|----:----|----:----|----:----|

18101 ATGCATGGCTTTCCCAAGGGGTAGGAGGTTCCATATTTGGCTGGAACGTTGGAAAGAATCCACCTCCATTTTTGCATGTATAAACTGGTTTCTTCAATTT 18200
      ----:----|----:----|----:----|----:----|----:----|----:----|----:----|----:----|----:----|----:----|

                            V  Y  R  T  A  L  H  S  D  C  F  I  S  L  E  P  G  Q  S  E  K  G  D  R  F  G     F1
                             Y  T  E  R  H  S  I  V  T  V  L  F  L  W  S  Q  D  K  V  K  R  E  T  D  L  V    F2
                              I  Q  N  G  T  P  *  *  L  F  Y  F  S  G  A  R  T  K  *  K  G  R  Q  I  W  C   F3
18201 TTCTTTTCCCTTTTTGCTTTAGGTATACAGAACGGCACTCCATAGTGACTGTTTTATTTCTCTGGAGCCAGGACAAAGTGAAAAGGGAGACAGATTTGGT 18300
      ----:----|----:----|----:----|----:----|----:----|----:----|----:----|----:----|----:----|----:----|

      V  F  D  V  I  S  L  D  S  T  L  I  I  F  L  N  I  S  L  *  I  Y  L  Q  L  Q  L  L  E  M  V  K  R  T     F1
       Y  L  M  S  S  A  W  T  P  H  *  L  S  S  L  T  Y  P  S  K  Y  T  F  S  F  S  F  W  R  W  *  R  G       F2
        I  *  C  H  Q  P  G  L  H  I  N  Y  L  P  *  H  I  P  L  N  I  P  S  A  S  A  S  G  D  G  K  E  D      F3
18301 GTATTTGATGTCATCAGCCTGGACTCCACATTAATTATCTTCCTTAACATATCCCTCTAAATATACCTTCAGCTTCAGCTTCTGGAGATGGTAAAGAGGA 18400
      ----:----|----:----|----:----|----:----|----:----|----:----|----:----|----:----|----:----|----:----|
```

```
        L  G  G  R  D  R  *  Y  K  E  P  C  S  P  S  D  Q  I  Q  I  Q  S  S  T  D  L  E  D  N  K  I  I  I     F1
       L  *  E  A  E  T  D  D  T  K  N  H  A  A  L  V  T  K  S  R  F  R  V  Q  Q  I  L  R  T  I  K  *  L  *    F2
       F  R  R  Q  R  Q  M  I  Q  R  T  M  Q  P  *  *  P  N  P  D  S  E  F  N  R  S  *  G  Q  *  N  N  Y      F3
18401 CTTTAGGAGGCAGAGACAGATGATACAAAGAACCATGCAGCCCTAGTGACCAAATCCAGATTCAGAGTTCAACAGATCTTGAGGACAATAAAATAATTAT 18500
      ----:----|----:----|----:----|----:----|----:----|----:----|----:----|----:----|----:----|----:----|
```

Cloning primer 2 ←

Cloning primer 1 ←

```
       N  N  I  I  F  F  G                                                                                F1
        I  I  S  F  F  L  V                                                                               F2
      K  *  Y  H  F  F  W  S                                                                              F3
18501 AAATAATATCATTTTTTTTGGTCA
      ----:----|----:----|----
```
