# Supplementary material for: Molecular cloning of PRD-like homeobox genes expressed in bovine oocytes and early IVF embryos
Source: BMC Genomics. 2024 Nov 6;25:1048. doi: 10.1186/s12864-024-10969-w (PMC11542365; doi:10.1186/s12864-024-10969-w)
Supplement: Supplementary file 6 — Supplementary Material 6: Additional file 12: Figure S4. The prediction of LEUTX derived from Bos taurus isolate L1 Dominette 01449 registration number 42190680 breed Hereford chromosome 18, ARS-UCD1.2, whole genome shotgun sequence. Three possible ORFs for exons, but not introns, are depicted. Putative protein sequence is highlighted in yellow. Sequences from StringTie merge prediction and confirmed cDNA are drawn as lines below the corresponding sequences. Cloning primers are drawn as line arrows. Splice sites are underlined and codons split by two exons are coloured red. The homeodomain is highlighted in green. [file 12864_2024_10969_MOESM6_ESM.pdf]

Supplementary Figure S4. The prediction of *LEUTX* derived from *Bos taurus* isolate L1 Dominette 01449 registration number 42190680 breed Hereford chromosome 18, ARS-UCD1.2, whole genome shotgun sequence. Three possible ORFs for exons, but not introns, are depicted. Putative protein sequence is highlighted in yellow. Sequences from StringTie merge prediction and confirmed cDNA are drawn as lines below the corresponding sequences. Cloning primers are drawn as line arrows. Splice sites are underlined and codons split by two exons are coloured red. The homeodomain is highlighted in green.

```
           S  H  *  A  C  *  V  D  D  G  R  V  W  H  *  G  G  V  G  W  G  H  D  F  V  K  I  L  L  K  Y  *  A    F1
            L  I  K  P  V  E  W  M  M  G  G  C  G  I  R  E  G  *  G  G  G  M  I  L  L  K  F  C  L  N  I  R  P   F2
          F  S  L  S  L  L  S  G  *  W  E  G  V  A  L  G  R  G  R  V  G  A  *  F  C  *  N  F  A  *  I  L  G  L   F3
49330645 TTCTCATTAAGCCTGTTGAGTGGATGATGGGAGGGTGTGGCATTAGGGAGGGGTAGGGTGGGGGCATGATTTTGTTAAAATTTTGCTTAAATATTAGGCC 49330744
         :----|----:----|----:----|----:----|----:----|----:----|----:----|----:----|----:----|----:----|----
StringTie merge

          S  K  P  S  R  L  Q  F  I  V  R  Q  H  L  V  E  S  L  C  F  S  R  I  S  H  G  R  *  A  G  Q  R  Q  C   F1
           L  N  P  A  D  Y  S  S  *  S  G  N  T  W  S  N  H  C  A  S  Q  G  F  L  M  A  G  K  L  G  K  G  N    F2
            *  T  Q  Q  T  T  V  H  S  Q  A  T  P  G  R  I  T  V  L  L  K  D  F  S  W  Q  V  S  W  A  K  A  M    F3
49330745 TCTAAACCCAGCAGACTACAGTTCATAGTCAGGCAACACCTGGTCGAATCACTGTGCTTCTCAAGGATTTCTCATGGCAGGTAAGCTGGGCAAAGGCAAT 49330844
         :----|----:----|----:----|----:----|----:----|----:----|----:----|----:----|----:----|----:----|----
                    Cloning primer 1
LEUTX_8c_01

49330845 GCATGCTGAAGATGCTAGAGGTTGAGTAGTTGGTTGTGATAAACTGAGGTGAGAGGGGCGAGAGTTTGTGGTAGTCTCTGAATTTTGCCAACACTGGCTC 49330944
         :----|----:----|----:----|----:----|----:----|----:----|----:----|----:----|----:----|----:----|----

49330945 ATCCGTCTGGGGAACCGTTGGGTTATACCAGCAGGGAGCATTTGAAAACATAGTGCTTACTGCATGACAGGTCAATAAATCAACAGACGGTTTTCTTTTC 49331044
         :----|----:----|----:----|----:----|----:----|----:----|----:----|----:----|----:----|----:----|----

49331045 GTTGTTTTTGGCTGTGCTGGGTCTTTGTTGCTGCTCGAGGGCCTTCTCTAGTCGTGGCGAGTGGAGGCTGCTCTTTGTTGTGGTGTCCGGACTTCTCGTT 49331144
         :----|----:----|----:----|----:----|----:----|----:----|----:----|----:----|----:----|----:----|----

49331145 GCTTCTCTTGTTGAAGAGCACAGGCTCTAGGGGCTTTGAGGGATGTGGAATCTTCCCAGGCCAGGGATCGAACCCATATCCCCTGCATTGGCAGGCAGAT 49331244
         :----|----:----|----:----|----:----|----:----|----:----|----:----|----:----|----:----|----:----|----

49331245 TCCTATCCCCAGGGAAGTCCACAGACAAGTTGTTGGGACAGGGAATATCGACTTTATTCAGAAAGCCAGCAGACCAAGAGGATGGTGGACTTGAGTCCCA 49331344
         :----|----:----|----:----|----:----|----:----|----:----|----:----|----:----|----:----|----:----|----

49331345 AAGAACCTCTGTGTCTGAGTTACAATTCAGGCCTCTTTTATTCCAAAAGGGGAGGAAGTAATGTCAAGCACTTCCTGGTTCCCATCAACCTCCGGAGGGA 49331444
         :----|----:----|----:----|----:----|----:----|----:----|----:----|----:----|----:----|----:----|----

49331445 ATGTGTTAATAACTTTCCCGCAGTCATCCTCAGGTGAGCCTGGTTAAGATATTTTCTATATGGTAAACAAAGATATTTTAGCTTAATGATCATTACCTGG 49331544
         :----|----:----|----:----|----:----|----:----|----:----|----:----|----:----|----:----|----:----|----
```

```
49331545 GAGGGTTCCCAGAGATGGGCTGTTATGTATAATTTAAGCTTTGGGGAACATTCCTTTCGTGATCTAACATGTAGTAGAAAATTACATCACCAGTAATGTA 49331644
          :----|----:----|----:----|----:----|----:----|----:----|----:----|----:----|----:----|----:----|----

49331645 AGTGTTTGTTTCTCCACCTCCTTTTTAACAAGTGGTACTGTCATACTTTTAAATGTCAACCATTTGGTGGTGTTCCGAAAACAATATTCAACTGAGTAAA 49331744
          :----|----:----|----:----|----:----|----:----|----:----|----:----|----:----|----:----|----:----|----

49331745 TTTTAAAGATTGTATTGGCTTTATTCAGTGATTCATGAATCAGGCAGGATCCAGTCTAGCATATCAAAAGGAGCTCAGAGGAGCTGTGTAAAATGAATGA 49331844
          :----|----:----|----:----|----:----|----:----|----:----|----:----|----:----|----:----|----:----|----

49331845 TTTGTATTGTACAGGCAGAAGGGAATAGGAGCTAGGTTATACAAGGCAGAAAATCAGATTATTACAGGAACTAGGTTATACAAGGTGGGAAATCAGATTA 49331944
          :----|----:----|----:----|----:----|----:----|----:----|----:----|----:----|----:----|----:----|----

49331945 TTGCACGGTTACTTCCCTTTATGAGATAGCAGGGGTCTATCAGGCAGTTACCTAACTAGTACTGATTGAGCAATTCCTGATGTGCTTAAGATTCCATTTC 49332044
          :----|----:----|----:----|----:----|----:----|----:----|----:----|----:----|----:----|----:----|----

49332045 TGGGAGAGCTGAAACTGTAAATTGCCTGTTGGCAGTATGGAGCATACACGACTCCATTTTGGGCCTTTCATTTGTAATAGTGGGTACATGAAATTTGATC 49332144
          :----|----:----|----:----|----:----|----:----|----:----|----:----|----:----|----:----|----:----|----

49332145 CAGTTCCATTTCTTTATTAACCAATGAGTCTCTTCACTCTATTTTAAAGTCTATTTTGCTTTTCTGTTTTGCTAATTTCCTACTTCTTTCTTTTGACGTT 49332244
          :----|----:----|----:----|----:----|----:----|----:----|----:----|----:----|----:----|----:----|----

49332245 TTTCTAGTGTATTTCCCACCCCCCCCACCCCCGACCTTTGAGGCTTGGTAGAACATTTTTGTTGTATTCTGGACACCAATAATTTATTTCTTATATTGGT 49332344
          :----|----:----|----:----|----:----|----:----|----:----|----:----|----:----|----:----|----:----|----

49332345 TGCAGATGTACATGTACGTGTACTCAGTCATGTCTTGACTCTGAGATTCCATGGACTGTAGCCCACCAGGTTCCTCTGTCCACAGAATTTCCCAGGCAAG 49332444
          :----|----:----|----:----|----:----|----:----|----:----|----:----|----:----|----:----|----:----|----

49332445 AATACTGGAGTGGGTTGCCATTTCCTCCCCCCAGGAATCAGCTCTGCTTGCTTTCCAGGCCTGTTGTATAGGACTCAACCTGGAAGCTCCCCTTGTCACC 49332544
          :----|----:----|----:----|----:----|----:----|----:----|----:----|----:----|----:----|----:----|----

49332545 CTCTTGTGTGGATTCTCCTTTCTTCCATACAAATTCCCCTTTGTTTGTGACCACCACCCCCCAACCCCTGCACTGGGTGGCATGGGAGATCTTAGTTTTT 49332644
          :----|----:----|----:----|----:----|----:----|----:----|----:----|----:----|----:----|----:----|----

49332645 GACCAGGAATTGAACCCATATCTCCGTGCATGGGAAGCTCAGATTCTTAACCTCTGGACCACGAGGGAAGGCCCCTCCCTCTTGTTCTTTATTTTGCTGA 49332744
          :----|----:----|----:----|----:----|----:----|----:----|----:----|----:----|----:----|----:----|----

49332745 GTTGAACTATATCCTCAAGTAGCACACAAAATAAGTTGGTTGAACCTATGAGTGTCTGAATGTATCTTTATTCTCTTCTCACGTGGAAGGATAAATTTGA 49332844
          :----|----:----|----:----|----:----|----:----|----:----|----:----|----:----|----:----|----:----|----

49332845 CTGCATAGAGAATTTTATCTTGAAAGTCATTTCCTTAAGAATGTAATATTAAAGTCCCCAACTATTACAGTGTTGCTGTATATTTCTCCCTTTATTTCTT 49332944
          :----|----:----|----:----|----:----|----:----|----:----|----:----|----:----|----:----|----:----|----

49332945 TACTATTTACTTTCTATATTTATGTCCTCCTATGCTGGGGGCAGAGGTGTCTACAAATGTATCTTCTTGTTGGATTGACCCCTTTATCCTCCTGTAATGC 49333044
```

```
         :----|----:----|----:----|----:----|----:----|----:----|----:----|----:----|----:----|----:----|----

49333045 CCTTTTTGTCTTTTGTTACAGTCTTTTGTTTTAAAGTGTATTTCTTCTGATGCAAGTATTGCTGCCCCAGCATTCTTTTCCTTCCCACTGGCGCTGCTGT 49333144
         :----|----:----|----:----|----:----|----:----|----:----|----:----|----:----|----:----|----:----|----

49333145 TGTTGTTCGGTCACTTAGTCGTGTCCGACTCTTTGCAACCCTGTGGATTGCAGCTCACCAGGCCTCCCTGTCCTTCACCATCTCCTGGATCCTGCTCAAA 49333244
         :----|----:----|----:----|----:----|----:----|----:----|----:----|----:----|----:----|----:----|----

49333245 CTCATGTCCATTGAGTCAGTGATGTCATCCACCCATCTCGTCCTCTGTCATCCCCTTCTCCTCCTGCCTTCAGTCTTTTTCTCCATCAGGGTCTCTTGCA 49333344
         :----|----:----|----:----|----:----|----:----|----:----|----:----|----:----|----:----|----:----|----

49333345 GTGAGTCAGCTCTTCTCATTAGGTGGCCAAAGTACTGGAGCTTCAGCATCAGTCCTTCCAGTGAGTATTCAGGGTTGATTTTCTTTAGGATTGACCCCTT 49333444
         :----|----:----|----:----|----:----|----:----|----:----|----:----|----:----|----:----|----:----|----

49333445 GGCATGGTATATCTTTTTTGATCCTTTCACTTTCATCCTCTGTCTGCAGTTTTGAAGTGTATCTTTTATAGACAGCATATAAAAGGCATATATTAATAGA 49333544
         :----|----:----|----:----|----:----|----:----|----:----|----:----|----:----|----:----|----:----|----

49333545 AGCTTTAAAAAATCCATTTAGCCACCTAGGTTTTTTGATTGGAGAGTTTATTCCATTTACATTTAAAGTAATTATTGATGGATGTGTAATTGCCATTGCA 49333644
         :----|----:----|----:----|----:----|----:----|----:----|----:----|----:----|----:----|----:----|----

49333645 AAAATTGTTTTCTGGCTTTTTTTATAGTTCCTCCTTCTCTTTCTCTCTTACCTTGTGGTTTGATTGTTTTCTTTAGTGTTCTGTTTATATCTTTTCTCAT 49333744
         :----|----:----|----:----|----:----|----:----|----:----|----:----|----:----|----:----|----:----|----

49333745 TTTCTTTAGGTATCTAGTAAAAGTGTTTTCTTTGTTTTTACCATGAAGTTCACATCTATAATATCCTATATATTTTTAACCCCCCACACACATTTTATAT 49333844
         :----|----:----|----:----|----:----|----:----|----:----|----:----|----:----|----:----|----:----|----

49333845 TTGTATGACACGTTTTACATCTTTTTATTTCATGTATCTCTTGACAGGTTATTAATCTTGAATTGATTTTACTACTGTGTCTTTTACTCTTCCTGCTTGC 49333944
         :----|----:----|----:----|----:----|----:----|----:----|----:----|----:----|----:----|----:----|----

49333945 TTTGTATGTGACTTATCCACTTCTTTTATTATTGAATATATATATATATATATTTAAAAAATACTTATTTGGCTGCATTGGGTCTTACTTGAGGCATGCA 49334044
         :----|----:----|----:----|----:----|----:----|----:----|----:----|----:----|----:----|----:----|----

49334045 GGATCTTTTATTGCCATGCAAAGACCCTCTGGTTGTGGTGCATGGGCTCTCTAGTTGTGGTGTACCAGCTCCAGAGCATGTGGGCTGAGTAGTTAAATGG 49334144
         :----|----:----|----:----|----:----|----:----|----:----|----:----|----:----|----:----|----:----|----

49334145 ACTCATGGACTCAGCTGCTTTGCAGCACGTGGGGTCTTAGCTCCCTGACCAGGGCTCAAACCCATGTCCCCTGCATTGCAGGGCAGATTCTTAACCACTG 49334244
         :----|----:----|----:----|----:----|----:----|----:----|----:----|----:----|----:----|----:----|----

                                                                                           K  Q  N  S  T  R  R  F  R  T  R      F1
                                                                                         K  N  K  I  Q  L  G  D  S  A  H  A     F2
                                                                                          K  T  K  F  N  S  A  I  P  H  T  L    F3
 49334245 GACCAATGGGCAACTCCCTTGGAAGTATTTTTTAGGAAAGATTTTCATCTATTCTCTTCTAACAGAAAAACAAAATTCAACTCGGCGATTCCGCACACGC 49334344
          :----|----:----|----:----|----:----|----:----|----:----|----:----|----:----|----:----|----:----|----
```

```
          F N G E Q L G A L R D V F E R T R Y P H C F L I R T L A S T I H L D   F1
           L M E N S * E H * E M Y L K G P G T H I V S S * E H L L Q L F I L    F2
            * W R T A R S T K R C I * K D Q V P T L F P H K N T C F N Y S S *   F3
49334345 TTTAATGGAGAACAGCTAGGAGCACTAAGAGATGTATTTGAAAGGACCAGGTACCCACATTGTTTCCTCATAAGAACACTTGCTTCAACTATTCATCTTG 49334444
         :----|----:----|----:----|----:----|----:----|----:----|----:----|----:----|----:----|----:----|----

           A S V I K                                                                                           F1
          M R Q L *                                                                                            F2
           C V S Y K                                                                                           F3
49334445 ATGCGTCAGTTATAAAGGTATGTACCTGAGATCTGTCTCTATATAGCTAGATAGCACACCTAACCAGAGTTTCACACACATTTAGTCACCATGTAATTCT 49334544
         :----|----:----|----:----|----:----|----:----|----:----|----:----|----:----|----:----|----:----|----

49334545 CATTGCTTGAAAAATTGCTGTGTGCCAGGACAGATGTTAAGCATTTTATATATTTGTTCCACATTTGTTTTGAAGAACAAGAGTCACAGAGAGGAATCAT 49334644
         :----|----:----|----:----|----:----|----:----|----:----|----:----|----:----|----:----|----:----|----

49334645 CTTCCAAGAATTATAAACAGTAGAGCTAGAATTTGAACCCAGGCCTTTCTGACTCCAGACCCACGGTGCTAGATGAGATCCTTTGGTGATGGATTTGGGT 49334744
         :----|----:----|----:----|----:----|----:----|----:----|----:----|----:----|----:----|----:----|----

49334745 CTGTTCCTGTTTATTTCTAGATTCTCCTTAATCACAACATTCAGTTATCTATATACCTTGTGGCTAAATTCCCTTAGAGTACTCGGCATAAGAACTGCTG 49334844
         :----|----:----|----:----|----:----|----:----|----:----|----:----|----:----|----:----|----:----|----

49334845 GACGAATCCTAAAGGCACCCCAAGCTACACTTCCACTCCGTATTCTCAGGCTTAAGAATCCTCTCTGAGAACGCAGAGTATCCACCAAGACAAAACCCAC 49334944
         :----|----:----|----:----|----:----|----:----|----:----|----:----|----:----|----:----|----:----|----

49334945 TTTCTAAATACACGTCCTGTTCTTGGCTGAAAGCAGTCTGTCCTGTCATGCCCGTTGCCAGCGCAATCCTATGCCCCTTTGCCTCCTGGATTCCAGGACC 49335044
         :----|----:----|----:----|----:----|----:----|----:----|----:----|----:----|----:----|----:----|----

49335045 AGGAGAGGATAGAATTTTCCTCACTCAAATCAAACGTGAAGACATGTGAGAGTAACTTTCATATACCAGAACCAACCAAATTTCTGGAGTATATGAGGAT 49335144
         :----|----:----|----:----|----:----|----:----|----:----|----:----|----:----|----:----|----:----|----

                                                                L G L K T N V S K G G G R R I          F1
                                                                 L V * K P T C Q K E E G G E S         F2
                                                                T W F K N Q R V K R R R E E N Q        F3
49335145 TCTCCTTTGGCTGCTTCCCCCTCTTATTAACCTGCTCATCTCTTCCCTCTCCAGACTTGGTTTAAAAACCAACGTGTCAAAAGGAGGAGGGAGGAGAATC 49335244
         :----|----:----|----:----|----:----|----:----|----:----|----:----|----:----|----:----|----:----|----

          R L S K I C H Q E T R A G L S Q * R R K R C P Y R A L P E A L I P R   F1
           D S A K S V T R R P A P G C L S E G G R D A L T G H F R K H S S H    F2
            T Q Q N L S P G D P R R V V S V K E E E M P L P G T S G S T H P T   F3
49335245 AGACTCAGCAAAATCTGTCACCAGGAGACCCGCGCCGGGTTGTCTCAGTGAAGGAGGAAGAGATGCCCTTACCGGGCACTTCCGGAAGCACTCATCCCAC 49335344
         :----|----:----|----:----|----:----|----:----|----:----|----:----|----:----|----:----|----:----|----
```

```
          V  S  A  L  Q  V  I  L  I  T  S  Y  L  S  F  L  V  L  S  S  V  K  G  L  L  P  L  H  A  L  H  P  A    F1
         E  S  Q  P  C  R  *  F  S  S  R  A  T  *  A  F  L  C  *  A  V  *  R  G  C  C  H  S  M  P  F  I  L  Q   F2
          S  L  S  L  A  G  D  S  H  H  E  L  P  E  L  S  C  A  E  Q  C  E  G  A  A  A  T  P  C  P  S  S  C    F3
49335345 GAGTCTCAGCCTTGCAGGTGATTCTCATCACGAGCTACCTGAGCTTTCTTGTGCTGAGCAGTGTGAAGGGGCTGCTGCCACTCCATGCCCTTCATCCTGC 49335444
         :----|----:----|----:----|----:----|----:----|----:----|----:----|----:----|----:----|----:----|----

          I  S  *  L  L  *  V  S  E  T  L  I  F  L  G  P  P  V  L  M  T  W  I  N  L  Y  S  Y  T  T  Y  L  G    F1
           F  P  D  C  S  K  S  Q  R  H  *  S  S  L  G  L  Q  S  L  *  H  G  S  T  Y  T  V  I  R  L  T  W  G   F2
         N  F  L  T  A  L  S  L  R  D  T  D  L  P  W  A  S  S  P  Y  D  M  D  Q  L  I  Q  L  Y  D  L  P  G  D  F3
49335445 AATTTCCTGACTGCTCTAAGTCTCAGAGACACTGATCTTCCTTGGGCCTCCAGTCCTTATGACATGGATCAACTTATACAGTTATACGACTTACCTGGGG 49335544
         :----|----:----|----:----|----:----|----:----|----:----|----:----|----:----|----:----|----:----|----

         M  M  T  P  A  V  W  I  S  T  S  S  Q  S  A  P  A  G  G  L  W  P  A  L  I  T  I  M  R  T  N  T  A  T  F1
          *  *  P  Q  Q  S  G  S  V  P  L  P  R  V  L  Q  L  G  D  C  G  R  H  *  S  P  S  *  G  R  T  Q  L    F2
           D  D  P  S  S  L  D  Q  Y  L  F  P  E  C  S  S  W  G  T  V  A  G  T  D  H  H  H  E  D  E  H  S  Y   F3
49335545 ATGATGACCCCAGCAGTCTGGATCAGTACCTCTTCCCAGAGTGCTCCAGCTGGGGGACTGTGGCCGGCACTGATCACCATCATGAGGACGAACACAGCTA 49335644
         :----|----:----|----:----|----:----|----:----|----:----|----:----|----:----|----:----|----:----|----

          I  L  R  K  F  R  D  P  N  F  F  L  N  G  F  Y  L  C  L  A  V  L  G  L  C  C  C  V  G  F  S  P  V    F1
         Q  S  *  E  S  S  V  I  Q  I  F  F  *  M  V  F  I  Y  V  W  L  C  W  V  S  A  A  A  W  A  F  L  Q  L  F2
          N  P  E  K  V  P  *  S  K  F  F  F  K  W  F  L  F  M  F  G  C  A  G  S  L  L  L  R  G  L  F  S  S    F3
49335645 CAATCCTGAGAAAGTTCCGTGATCCAAATTTTTTTTAAATGGTTTTTATTTATGTTTGGCTGTGCTGGGTCTCTGCTGCTGCGTGGGCTTTTCTCCAGT 49335744
         :----|----:----|----:----|----:----|----:----|----:----|----:----|----:----|----:----|----:----|----

          A  G  D  G  S  R  P  L  V  A  V  L  G  L  L  L  L  W  S  T  G  S  R  C  A  G  P  V  A  A  P  F  C    F1
           Q  G  T  G  A  A  L  *  L  R  C  L  G  F  S  C  S  G  A  Q  A  L  G  V  Q  G  P  L  R  L  P  S  V   F2
         C  R  G  R  E  P  P  S  S  C  G  A  W  A  S  L  A  L  E  H  R  L  *  V  C  R  A  R  C  G  S  L  L  S  F3
49335745 TGCAGGGGACGGGAGCCGCCCTCTAGTTGCGGTGCTTGGGCTTCTCTTGCTCTGGAGCACAGGCTCTAGGTGTGCAGGGCCCGTTGCGGCTCCCTTCTGT 49335844
         :----|----:----|----:----|----:----|----:----|----:----|----:----|----:----|----:----|----:----|----

         P  L  S  A  S  S  T  E  F  Q  Y  K  G  Q  S  S  G  I  F  G  E  I  A  E  T  S  *  F  S  *  F  V  P  V  F1
          L  *  V  P  P  Q  P  N  F  S  I  K  G  S  H  Q  A  S  L  E  K  *  Q  K  L  A  S  S  P  S  L  S  Q    F2
           S  K  C  L  L  N  R  I  S  V  *  R  A  V  I  R  H  L  W  R  N  S  R  N  *  L  V  L  L  V  C  P  S   F3
49335845 CCTCTAAGTGCCTCCTCAACCGAATTTCAGTATAAAGGGCAGTCATCAGGCATCTTTGGAGAAATAGCAGAAACTAGCTAGTTCTCCTAGTTTGTCCCAG 49335944
         :----|----:----|----:----|----:----|----:----|----:----|----:----|----:----|----:----|----:----|----

           E  L  S  P  L  I  L  C  K  N  T  Y  F  S  *  A  S  L  S  K  K  K  N  K  T  T  I  R  D  L  C  S  D   F1
         L  S  Y  L  H  *  Y  Y  V  K  I  P  I  F  H  E  P  L  C  L  K  K  K  T  K  Q  Q  L  G  T  S  V  V  I  F2
          *  A  I  S  I  N  I  M  *  K  Y  L  F  F  M  S  L  F  V  *  K  K  K  Q  N  N  N  *  G  P  L  *  *    F3
49335945 TTGAGCTATCTCCATTAATATTATGTAAAAATACCTATTTTTCATGAGCCTCTTTGTCTAAAAAAAAAAACAAAACAACAATTAGGGACCTCTGTAGTGA 49336044
         :----|----:----|----:----|----:----|----:----|----:----|----:----|----:----|----:----|----:----|----

          P  R  V  R  T  P  H  F  Q  C  R  G  H  G  F  S  L  W  L  E  H  *  D  P  T  C  C  M  A  Q  L  K  K    F1
```

```
           R  G  L  G  L  H  T  S  N  A  G  D  M  G  S  V  S  G  W  S  T  K  I  P  H  A  A  W  H  S  *  K  N    F2
          S  E  G  *  D  S  T  L  P  M  Q  G  T  W  V  Q  S  L  V  G  A  L  R  S  H  M  L  H  G  T  A  E  K  I   F3
49336045 TCCGAGGGTTAGGACTCCACACTTCCAATGCAGGGGACATGGGTTCAGTCTCTGGTTGGAGCACTAAGATCCCACATGCTGCATGGCACAGCTGAAAAAA 49336144
         :----|----:----|----:----|----:----|----:----|----:----|----:----|----:----|----:----|----:----|----

         *  T  P  K  *  Q  P  P  P  K  N  A  F  K  S  V  T  L  A  I  D  W  M  R  R  S  Q  H  L  Y  L  T  K  K   F1
          K  H  P  N  N  N  P  P  Q  K  M  H  L  R  V  *  H  *  Q  *  I  G  *  G  G  P  S  T  C  T  *  Q  K    F2
           N  T  Q  I  T  T  P  P  K  K  C  I  *  E  C  D  I  S  N  R  L  D  E  E  V  P  A  L  V  P  D  K  K   F3
49336145 TAAACACCCAAATAACAACCCCCCCCAAAAAATGCATTTAAGAGTGTGACATTAGCAATAGATTGGATGAGGAGGTCCCAGCACTTGTACCTGACAAAAA 49336244
         :----|----:----|----:----|----:----|----:----|----:----|----:----|----:----|----:----|----:----|----

           N  N  D  L  I  I  I  H  *  *  E  *  L  W  E  S  S  G  V  Q  L  L  L  S  R  V  A  G  T  *  K  M  L   F1
         K  T  M  I  *  *  L  S  T  D  E  N  S  S  G  R  A  L  E  F  S  Y  Y  S  A  G  *  Q  A  H  E  R  C  S   F2
          K  Q  *  F  N  N  Y  P  L  M  R  I  A  L  G  E  L  W  S  S  V  T  T  Q  Q  G  S  R  H  M  K  D  A    F3
49336245 AAAACAATGATTTAATAATTATCCACTGATGAGAATAGCTCTGGGAGAGCTCTGGAGTTCAGTTACTACTCAGCAGGGTAGCAGGCACATGAAAGATGCT 49336344
         :----|----:----|----:----|----:----|----:----|----:----|----:----|----:----|----:----|----:----|----

          N  V  A  Y  H  L  G  N  A  N  R  N  C  S  E  V  S  P  H  P  Q  S  A  W  L  S  S  E  R  P  Q  E  M    F1
           T  W  L  I  I  W  G  M  Q  I  E  T  A  V  R  C  H  L  T  P  S  Q  H  G  C  H  L  K  D  H  K  K  C   F2
         Q  R  G  L  S  S  G  E  C  K  S  K  L  Q  *  G  V  T  S  P  P  V  S  M  A  V  I  *  K  T  T  R  N  V   F3
49336345 CAACGTGGCTTATCATCTGGGGAATGCAAATCGAAACTGCAGTGAGGTGTCACCTCACCCCCAGTCAGCATGGCTGTCATCTGAAAGACCACAAGAAATG 49336444
         :----|----:----|----:----|----:----|----:----|----:----|----:----|----:----|----:----|----:----|----

         L  A  R  T  G  R  K  G  E  P  S  D  T  N  K  S  T  S  P  W  E  M  E  A  F  Y  P  C  H  H  I  A  C  S   F1
          W  R  G  Q  G  E  K  G  S  L  Q  T  Q  T  R  A  H  P  H  G  K  W  K  P  F  T  H  V  T  T  *  L  A    F2
           G  E  D  R  E  K  R  G  A  F  R  H  K  Q  E  H  I  P  M  G  N  G  S  L  L  P  M  S  P  H  S  L  Q   F3
49336445 TTGGCGAGGACAGGGAGAAAAGGGGAGCCTTCAGACACAAACAAGAGCACATCCCCATGGGAAATGGAAGCCTTTTACCCATGTCACCACATAGCTTGCA 49336544
         :----|----:----|----:----|----:----|----:----|----:----|----:----|----:----|----:----|----:----|----

           Q  D  *  S  L  W  L  *  P  R  T  R  A  N  K  S  R  Q  A  L  S  T  F  T  K  E  Y  Q  G  A  L  G  L   F1
         A  K  T  D  P  C  G  C  D  L  A  L  E  Q  T  R  A  G  R  P  C  P  L  S  P  R  S  T  K  E  P  W  G  C   F2
          P  R  L  I  P  V  A  V  T  S  H  *  S  K  Q  E  Q  A  G  L  V  H  F  H  Q  G  V  P  R  S  P  G  A    F3
49336545 GCCAAGACTGATCCCTGTGGCTGTGACCTCGCACTAGAGCAAACAAGAGCAGGCAGGCCTTGTCCACTTTCACCAAGGAGTACCAAGGAGCCCTGGGGCT 49336644
         :----|----:----|----:----|----:----|----:----|----:----|----:----|----:----|----:----|----:----|----

          S  V  T  P  E  A  F  A  T  E  D  S  P  S  L  P  A  C  E  S  Q  L  T  E  L  *  G  I  R  A  *  A  F    F1
           Q  *  H  Q  K  L  L  P  L  R  T  P  Q  V  F  P  H  A  N  P  S  *  R  N  C  E  A  S  V  P  E  P  S   F2
         V  S  D  T  R  S  F  C  H  *  G  L  P  K  S  S  R  M  R  I  P  A  D  G  T  V  R  H  P  C  L  S  L  Q   F3
49336645 GTCAGTGACACCAGAAGCTTTTGCCACTGAGGACTCCCCAAGTCTTCCCGCATGCGAATCCCAGCTGACGGAACTGTGAGGCATCCGTGCCTGAGCCTTC 49336744
         :----|----:----|----:----|----:----|----:----|----:----|----:----|----:----|----:----|----:----|----

         K  S  R  S  *  C  C  S  F  I  E  H  W  H  S  R  M  V  R  K  P  M  S  L  I  R  G  C  D  C  L  L  T  C   F1
          R  A  G  A  D  A  A  A  S  S  N  T  G  T  H  V  W  C  E  N  P  C  R  *  S  E  D  V  T  A  C  *  P    F2
           E  Q  E  L  M  L  Q  L  H  R  T  L  A  L  T  Y  G  A  K  T  H  V  A  D  Q  R  M  *  L  P  V  D  L   F3
```

```
49336745 AAGAGCAGGAGCTGATGCTGCAGCTTCATCGAACACTGGCACTCACGTATGGTGCGAAAACCCATGTCGCTGATCAGAGGATGTGACTGCCTGTTGACCT 49336844
         :----|----:----|----:----|----:----|----:----|----:----|----:----|----:----|----:----|----:----|----
                                 Cloning primer 1 <-------------

           G  M  D  V  A  S  E  G  L  L  A  P  S  S  V  L  C  T  S  I  L  P  A  F  P  I  A  G  A  V  T  R  T    F1
         V  G  W  T  *  P  V  R  D  S  S  P  H  L  L  S  F  A  R  P  F  C  L  H  F  P  *  Q  E  L  S  Q  E  R   F2
          W  D  G  R  S  Q  *  G  T  P  R  P  I  F  C  P  L  H  V  H  S  A  C  I  S  H  S  R  S  C  H  K  N     F3
49336845 GTGGGATGGACGTAGCCAGTGAGGGACTCCTCGCCCCATCTTCTGTCCTTTGCACGTCCATTCTGCCTGCATTTCCCATAGCAGGAGCTGTCACAAGAAC 49336944
         :----|----:----|----:----|----:----|----:----|----:----|----:----|----:----|----:----|----:----|----

          Q  S  *  E  R  N  M  L  L  R  P  P  G  L  N  T  *  L  N  P  V  K  A  A  I  *  T  F  *  D  L  V  G     F1
           S  L  E  R  G  T  C  C  *  D  H  L  D  *  I  H  D  *  T  Q  L  R  P  P  Y  K  H  F  K  I  W  W  V    F2
         A  V  L  R  E  E  H  V  A  E  T  T  W  T  E  Y  M  T  E  P  S  *  G  R  H  I  N  I  L  R  F  G  G  W   F3
49336945 GCAGTCTTGAGAGAGGAACATGTTGCTGAGACCACCTGGACTGAATACATGACTGAACCCAGTTAAGGCCGCCATATAAACATTTTAAGATTTGGTGGGT 49337044
         :----|----:----|----:----|----:----|----:----|----:----|----:----|----:----|----:----|----:----|----

         G  C  G  D  L  L  V  L  C  P  P  R  M  S  F  V  I  K  F  L  C  S  F  K  L  P  P  T  N  L  L  *  S  A   F1
          D  V  E  T  Y  L  S  C  V  H  P  G  *  A  L  *  L  S  F  F  V  H  L  S  C  H  P  P  I  C  C  D  L     F2
           M  W  R  P  T  C  L  V  S  T  Q  D  E  L  C  N  *  V  S  L  F  I  *  A  A  T  H  Q  S  V  V  I  C    F3
49337045 GGATGTGGAGACCTACTTGTCTTGTGTCCACCCAGGATGAGCTTTGTAATTAAGTTTCTTTGTTCATTTAAGCTGCCACCCACCAATCTGTTGTGATCTG 49337144
         :----|----:----|----:----|----:----|----:----|----:----|----:----|----:----|----:----|----:----|----

           S  F  F  T  L  F  W  G  P  V  Y  E  S  L  G  Q  Y  *  E  M  E  *  M  K  W  N  S  I  K  T  K  V  V    F1
         P  L  S  S  L  S  S  G  D  Q  F  M  N  H  *  V  S  T  K  K  W  N  E  *  N  G  T  R  L  K  L  R  W  L   F2
          L  F  L  H  S  L  L  G  T  S  L  *  I  I  R  S  V  L  R  N  G  M  N  E  M  E  L  D  *  N  *  G  G     F3
49337145 CCTCTTTCTTCACTCTCTTCTGGGGACCAGTTTATGAATCATTAGGTCAGTACTAAGAAATGGAATGAATGAAATGGAACTCGATTAAAACTAAGGTGGT 49337244
         :----|----:----|----:----|----:----|----:----|----:----|----:----|----:----|----:----|----:----|----

          N  K  K  T  T  K  V  *  S  S  T  T  K  V  G  W  A  K  R  T  P  L  L  S  R  Q  L  C  F  L  *  F  R     F1
           T  K  K  Q  L  R  C  N  P  V  L  Q  R  L  G  G  P  K  G  L  L  Y  C  P  V  S  S  V  F  S  S  S  E    F2
         *  Q  K  N  N  *  G  V  I  Q  Y  Y  K  G  W  V  G  Q  K  D  S  S  T  V  P  S  A  L  F  S  L  V  Q  K   F3
49337245 TAACAAAAAAACAACTAAGGTGTAATCCAGTACTACAAAGGTTGGGTGGGCCAAAAGGACTCCTCTACTGTCCCGTCAGCTCTGTTTTCTCTAGTTCAGA 49337344
         :----|----:----|----:----|----:----|----:----|----:----|----:----|----:----|----:----|----:----|----

         K  N  L  K  V  K  D  D  N  E  K  S  D  L  P  S  F  *  A  *  V  R  Q  S  D  P  N  E  D  *  Q  R  V  R   F1
          K  I  *  K  S  K  M  I  M  R  N  L  T  C  H  H  F  E  H  R  L  G  R  V  I  Q  M  K  I  D  K  E  S     F2
           K  F  K  S  Q  R  *  *  *  E  I  *  P  A  I  I  L  S  I  G  *  A  E  *  S  K  *  R  L  T  K  S  Q    F3
49337345 AAAAATTTAAAAGTCAAAGATGATAATGAGAAATCTGACCTGCCATCATTTTGAGCATAGGTTAGGCAGAGTGATCCAAATGAAGATTGACAAAGAGTCA 49337444
         :----|----:----|----:----|----:----|----:----|----:----|----:----|----:----|----:----|----:----|----

           V  S  L  L  R  T  Q  S  L  F  T  Q  Q  *  V  K  R  V  S  G  G  K  R  S  F  Q  V  S  V  M  C  E  P    F1
         G  S  L  C  *  G  P  K  A  F  L  H  N  S  E  *  K  G  S  V  V  E  R  E  V  S  R  S  L  *  C  V  S  P   F2
          G  L  F  A  E  D  P  K  P  F  Y  T  T  V  S  K  K  G  Q  W  W  K  E  K  F  P  G  L  C  D  V  *  A     F3
```

```
49337445 GGGTCTCTTTGCTGAGGACCCAAAGCCTTTTTACACAACAGTGAGTAAAAAGGGTCAGTGGTGGAAAGAGAAGTTTCCAGGTCTCTGTGATGTGTGAGCC 49337544
         :----|----:----|----:----|----:----|----:----|----:----|----:----|----:----|----:----|----:----|----

          H  Q  L  G  Y  Q  N  P  V  V  K  S  *  H  E  L  *  L  D  *  E  H  K  R  E  G  L  M                  F1
           T  S  W  D  T  R  T  Q  W  *  N  P  D  M  N  C  N  *  T  E  N  I  N  V  K  G  *  W                 F2
         P  P  A  G  I  P  E  P  S  G  E  I  L  T  *  T  V  I  R  L  R  T  *  T  *  R  A  D                    F3
49337545 CCACCAGCTGGGATACCAGAACCCAGTGGTGAAATCCTGACATGAACTGTAATTAGACTGAGAACATAAACGTGAAGGGCTGATGG              49337644
         :----|----:----|----:----|----:----|----:----|----:----|----:----|----:----|----:----|
```
