# Supplementary material for: Molecular cloning of PRD-like homeobox genes expressed in bovine oocytes and early IVF embryos
Source: BMC Genomics. 2024 Nov 6;25:1048. doi: 10.1186/s12864-024-10969-w (PMC11542365; doi:10.1186/s12864-024-10969-w)
Supplement: Supplementary file 7 — Supplementary Material 7: Additional file 13: Figure S5. The prediction of NOBOX derived from Bos taurus isolate L1 Dominette 01449 registration number 42190680 breed Hereford chromosome 4, ARS-UCD1.2, whole genome shotgun sequence. Three possible ORFs for exons, but not introns, are depicted. Putative protein sequence is highlighted in yellow. Sequences from StringTie merge prediction and confirmed cDNA are drawn as lines below the corresponding sequences. Cloning primers are drawn as line arrows. Splice sites are underlined and codons split by two exons are coloured red. The homeodomain is highlighted in green. [file 12864_2024_10969_MOESM7_ESM.pdf]

Supplementary Figure S5. The prediction of *NOBOX* derived from *Bos taurus* isolate L1 Dominette 01449 registration number 42190680 breed Hereford chromosome 4, ARS-UCD1.2, whole genome shotgun sequence. Three possible ORFs for exons, but not introns, are depicted. Putative protein sequence is highlighted in yellow. Sequences from StringTie merge prediction and confirmed cDNA are drawn as lines below the corresponding sequences. Cloning primers are drawn as line arrows. Splice sites are underlined and codons split by two exons are coloured red. The homeodomain is highlighted in green.

```
      G  F  F  R  R  R  A  G  G  R  P  R  A  G  V  Q  A  M  E  P  T  R  E  R  G  P  E  L  R        F1
       A  S  L  G  G  G  Q  A  G  G  P  V  R  E  S  R  P  W  S  P  P  G  S  A  A  R  S  C          F2
     R  L  L  *  A  A  G  R  R  A  A  P  C  G  S  P  G  H  G  A  H  P  G  A  R  P  G  V  A         F3
5001 AGGCTTCTTTAGGCGGCGGGCAGGCGGGCGGCCCCGTGCGGGAGTCCAGGCCATGGAGCCCACCCGGGAGCGCGGCCCGGAGTTGCGGGGTGAGTGCGCG 5100
     ----:----|----:----|----:----|----:----|----:----|----:----|----:----|----:----|----:----|----:----|
StringTie merge
Cloning primer
NOBOX_MII_01
NOBOX_MII_02

5101 GGAGCCCGGGGGCCCAGCAGCCAGGCCTCTAGAGCTCGGGAGGGTCGGGGCTGGGAGCTGAGGGGGACTCGAGGGGGTGTCGCGGGCCAGGGAGCCCTCC 5200
     ----:----|----:----|----:----|----:----|----:----|----:----|----:----|----:----|----:----|----:----|

5201 CCACAGCACCCTTCCGTTTTGGGGTTCTGATCTGTCTACACGCAGAGAAGGAAAGCAGGGTTGAGGCTCTGCTGTTCTAGGTTCCAAGACCCATCACCTT 5300
     ----:----|----:----|----:----|----:----|----:----|----:----|----:----|----:----|----:----|----:----|

5301 TTTTACCAGGGTCTATCACCCTGATTTGTTGTTCTTCAGTCGCTCAGTCGTGTCCGACTCTTTGCGACCCCATGAACTGTAGTCCACCAGGCTGTTCTGT 5400
     ----:----|----:----|----:----|----:----|----:----|----:----|----:----|----:----|----:----|----:----|

5401 CCATGGAATTCTCCAGGGAAGGACACTCTAGGCAAAAGAGTGCCATGCCCTTCTCCAGTGCGTCTTCCTGACTGAGGGTTGGAACGGGGCTCTTGCATTG 5500
     ----:----|----:----|----:----|----:----|----:----|----:----|----:----|----:----|----:----|----:----|

5501 GCAGGCGGATTCTTTACCGCTGAGCCACTTTAGGAAAGCTATCAAGGTGATGCATTGCCTTTAGTGACCATCTACTGTGTACCAGATTTGCTAGGCTCCA 5600
     ----:----|----:----|----:----|----:----|----:----|----:----|----:----|----:----|----:----|----:----|

5601 GTGCTCCAGAAATACTGTAAAACCAGTGTGTCCCTTCTTCCGCATTTCCCCATCCGGCCACTCGGCTTAGTTATGGAGTAGAAGCCTGCAAACCCTGGTC 5700
     ----:----|----:----|----:----|----:----|----:----|----:----|----:----|----:----|----:----|----:----|

5701 ACCAGCTTGTACGCTAGGGAACAATCTGGCAAAGCACCTTAGCCTGTAACCTACATCTTTCTTTACATGTTGTTAGAAAGCGGCTCCTTAAGCTAGTCAG 5800
     ----:----|----:----|----:----|----:----|----:----|----:----|----:----|----:----|----:----|----:----|

5801 TCTCATGCAACTTTCATTAAAAAAAAAAAAAAACCAGAAAAACTCGCTCTTGGAAACTTCCCTGATGGTCTAGAGGTTCAGATTTGGCACTTCCAATGCAG 5900
     ----:----|----:----|----:----|----:----|----:----|----:----|----:----|----:----|----:----|----:----|

5901 GGGGCCTGGGTAGACCCCTAGTGGAGGAACCAAGATCCCACATGCTGTGGGGGCCAAAACAAAACACCAGTGAGCATCCACTGAAAAATATTAAAAAAAA 6000
```

```
     ----:----|----:----|----:----|----:----|----:----|----:----|----:----|----:----|----:----|----:----|

6001 ATTTTTTTTAATAAAAATATTCTAGTTTTGTAAGAAATAACACAACAGCTCCTTGACCTGAGAGAGTTCTGGAGGTTATGAATAAGCTGGGAGGGAAATG 6100
     ----:----|----:----|----:----|----:----|----:----|----:----|----:----|----:----|----:----|----:----|

6101 AGGGGCAGGTTGGTGACCCTCGGAAGGGCTCCGTCTAGTGAACAAGAGCCCTGCACTGTGAGTTTAGGCAAGTCACTGAACTCTGCCATCTGCATTTCTT 6200
     ----:----|----:----|----:----|----:----|----:----|----:----|----:----|----:----|----:----|----:----|

6201 CATCTGTTAGAGGGAGAGATGCCTATGCCTTCCTCACAGGGATTAAATGCAGACCGGACTGATCTCTGAAAGGTGCTTAGCACCATGCCCAGGACCTGGT 6300
     ----:----|----:----|----:----|----:----|----:----|----:----|----:----|----:----|----:----|----:----|

6301 AAACTCCTGATGAATCATAGCTCAGAAAAGGACAGACAAGCTGAAGGCTGAGGGATGGGAATGTACAGTGCTCGTCAGGTCACCCTTCCATACCTGTAAA 6400
     ----:----|----:----|----:----|----:----|----:----|----:----|----:----|----:----|----:----|----:----|

6401 AGGGGGGCTGGCCTGGGCTCCAGGCCCTTGGCTGAGGTCCCGCTTTGCAGGGAGGGGATGAGGGTGTGTATAGGGGTGAGGAGAGAGCCAGTTCTGTGGG 6500
     ----:----|----:----|----:----|----:----|----:----|----:----|----:----|----:----|----:----|----:----|

6501 AGATGAGGGTAGACATTCCTGTTAGCTCCCCTTTGATGTTCTTGTCTGGGCCCCTGGACACCACTCAGTCCTGCCGTGTTCCTGCTCAGCCGTGATGCTG 6600
     ----:----|----:----|----:----|----:----|----:----|----:----|----:----|----:----|----:----|----:----|

6601 TGTCCGGAGCAGGACCGGGATCCAGTCTGATCAGCTGCAGAGTCTCAATCTCAAACTCACAATCCAGGTGTCTAGGAGAAAACTGGGAACTCCCAGGGAG 6700
     ----:----|----:----|----:----|----:----|----:----|----:----|----:----|----:----|----:----|----:----|

6701 TAAGTTCCCCCCAGATTAGGATTCCTCCCCCACCATCTAGGTTCACGAGCTCAGACCCCTCATGGTGGCATCTGCGTGCTTTTGCTTTTCCAAGCTACGT 6800
     ----:----|----:----|----:----|----:----|----:----|----:----|----:----|----:----|----:----|----:----|

6801 ATGTACGTATGTGTTAGCTCAGTCGTGTCCGACTCTTTGTTACCCCGTGGACTGTAGCCTGCCAGGCTTCTCTGTCCATGGAATTCTCCAGGCAAGAAGG 6900
     ----:----|----:----|----:----|----:----|----:----|----:----|----:----|----:----|----:----|----:----|

6901 ATTCCCTCCTGTGATCCTCACGGTAGCCGAGTTAGGAAGGTACGCCTGGAACTGTTACCGCGTTTGTTAAGTAGACAAGTGAAAGCTTAAGGTCAAACAG 7000
     ----:----|----:----|----:----|----:----|----:----|----:----|----:----|----:----|----:----|----:----|

                                                                                          A  R  G  V    F1
                                                                                           P  G  G  C   F2
                                                                                            Q  G  G     F3
7001 GACACCAGTTGCAGACCAGGAAGCAGCGCATCCTCCTGGGCCAGGCTCTTCCTTCTCCTGTGCTGAGTCGGGGTGCTGGCTCTCCACAGGCCAGGGGGGT 7100
     ----:----|----:----|----:----|----:----|----:----|----:----|----:----|----:----|----:----|----:----|

      S  L  L  P  G  W  R  R  R  R  K  P  C  R  G  Q  P  P  T  L  R  M  P  Q  A  R  T  C  L  F  P  A  P    F1
       P  C  C  Q  A  G  G  G  G  G  S  P  A  E  V  S  P  P  H  S  G  C  P  R  Q  G  R  A  Y  F  L  H  H   F2
     V  P  A  A  R  L  E  E  E  E  E  A  L  Q  R  S  A  P  H  T  Q  D  A  P  G  K  D  V  P  I  S  C  T  I F3
7101 GTCCCTGCTGCCAGGCTGGAGGAGGAGGAGGAAGCCCTGCAGAGGTCAGCCCCCCACACTCAGGATGCCCCAGGCAAGGACGTGCCTATTTCCTGCACCA 7200
     ----:----|----:----|----:----|----:----|----:----|----:----|----:----|----:----|----:----|----:----|
```

```
      S  T  G  R  S  G  R  Q  R  P  L  G  K  G  L  G  L  *  R  P  A  S  A  P  A  Q  E  L  S  T  K  T  G  A    F1
       L  R  G  E  A  A  V  R  G  P  W  G  R  D  W  A  Y  E  G  L  P  A  P  Q  P  R  S  S  P  R  R  Q  E     F2
        Y  G  E  K  R  P  S  E  A  P  G  E  G  T  G  P  M  K  A  C  Q  R  P  S  P  G  A  L  H  E  D  R  S    F3
7201 TCTACGGGGAGAAGCGGCCGTCAGAGGCCCCTGGGGAAGGGACTGGGCCTATGAAGGCCTGCCAGCGCCCCAGCCCAGGAGCTCTCCACGAAGACAGGAG 7300
     ----:----|----:----|----:----|----:----|----:----|----:----|----:----|----:----|----:----|----:----|

        W  P  R  L  D  P  S  L  K  G  K  G  L  P  S  R  G  Q  R  A  G  W  G  R  G  P  L  L  Q  P  P  A  S    F1
      P  G  L  A  *  T  P  A  S  R  A  R  A  S  L  P  G  D  R  G  Q  A  G  E  E  A  L  F  S  S  H  R  Q  A  F2
       L  A  S  P  R  P  Q  P  Q  G  Q  G  P  P  F  P  G  T  E  G  R  L  G  K  R  P  S  S  P  A  T  G  K     F3
7301 CCTGGCCTCGCCTAGACCCCAGCCTCAAGGGCAAGGGCCTCCCTTCCCGGGGACAGAGGGCAGGCTGGGGAAGAGGCCCTCTTCTCCAGCCACCGGCAAG 7400
     ----:----|----:----|----:----|----:----|----:----|----:----|----:----|----:----|----:----|----:----|

       R  R  S  L  G  M  W  V  R  P  R  R  H  L  P  A  A  L  T  R  L  G  P  R  T  T  Q  G  L  V  G  *  A     F1
        E  E  A  *  G  C  G  S  G  L  D  G  I  S  Q  Q  R  *  P  G  S  G  H  V  Q  P  R  A  L  W  V  R  P    F2
      Q  K  K  P  R  D  V  G  P  A  S  T  A  S  P  S  S  V  N  P  A  R  A  T  Y  N  P  G  P  C  G  L  G  R  F3
7401 CAGAAGAAGCCTAGGGATGTGGGTCCGGCCTCGACGGCATCTCCCAGCAGCGTTAACCCGGCTCGGGCCACGTACAACCCAGGGCCTTGTGGGTTAGGCC 7500
     ----:----|----:----|----:----|----:----|----:----|----:----|----:----|----:----|----:----|----:----|

      G  G  P  A  M  W  P  T  S  S  T  H  W  P  R  R  T  K  T  W  T  R  R  R  G  P  R  K  *  P  A  R  S  G  F1
       G  V  L  P  C  G  Q  P  P  Q  H  T  G  P  E  E  P  K  P  G  P  G  E  E  V  P  G  S  D  L  P  G  P    F2
        G  S  C  H  V  A  N  L  L  N  T  L  A  Q  K  N  Q  N  L  D  Q  E  K  R  S  P  E  V  T  C  Q  V  R   F3
7501 GGGGGTCCTGCCATGTGGCCAACCTCCTCAACACACTGGCCCAGAAGAACCAAAACCTGGACCAGGAGAAGAGGTCCCCGGAAGTGACCTGCCAGGTCCG 7600
     ----:----|----:----|----:----|----:----|----:----|----:----|----:----|----:----|----:----|----:----|

        R  R  L  A  P  C  T  A  R                                                                         F1
      E  E  D  S  H  P  V  P  L                                                                           F2
       K  K  T  R  T  L  Y  R  S                                                                          F3
7601 GAAGAAGACTCGCACCCTGTACCGCTCGGGTAAGTCCCTGAGGTGAGAGGAAACCGGAGGGACCTGGTGTCAATCTCTGGGAACGTGGCCCCAGGAGACG 7700
     ----:----|----:----|----:----|----:----|----:----|----:----|----:----|----:----|----:----|----:----|

7701 GGAGTGCAGTTAGCTTATGCATCACCTTGGCTGGAACGTCTCTTGACCTCTGCAAGGCCATGACTTCTGACCCTAAGAGGGGAAGTTTGCGTTTTCTTAT 7800
     ----:----|----:----|----:----|----:----|----:----|----:----|----:----|----:----|----:----|----:----|

7801 CAGAACATGGAAGAAACAGGAAAGTAACCCACCACGCAGCTCCCTGCCACAAAGCCCAAGCCAGAGGAGGCCCCAGCCCACTGGGGGAACTTAGCCACCA 7900
     ----:----|----:----|----:----|----:----|----:----|----:----|----:----|----:----|----:----|----:----|

7901 GGCTGTGGGAGGCCCTTGGGTTGGGGACAGTGGTCCCGCCTGAAAGGCGTGTGTTTCTCCTCTGCTTCCTCATTACTGGTGTTCATCCTCAAAGCCAGTG 8000
     ----:----|----:----|----:----|----:----|----:----|----:----|----:----|----:----|----:----|----:----|

                       Q  L  E  E  L  E  R  L  F  Q  D  D  H  Y  P  D  S  D  K  R  R  E  I  A  Q  T  V  G    F1
                      T  S  W  R  S  *  K  G  S  S  K  M  T  T  I  Q  T  A  I  S  A  V  R  L  P  R  Q  W  G   F2
                       P  A  G  G  A  R  K  A  L  P  R  *  P  L  S  R  Q  R  *  A  P  *  D  C  P  D  S  G  G  F3
8001 TTTTCTTCTTCTAGACCAGCTGGAGGAGCTAGAAAGGCTCTTCCAAGATGACCACTATCCAGACAGCGATAAGCGCCGTGAGATTGCCCAGACAGTGGGG 8100
     ----:----|----:----|----:----|----:----|----:----|----:----|----:----|----:----|----:----|----:----|
```

```
     V  T  P  Q  R  I  M                                                                                   F1
      S  P  P  S  A  S                                                                                     F2
       H  P  P  A  H  H                                                                                    F3
8101 GTCACCCCCCAGCGCATCATGGTAAAGGGTGCTGACTCCCCCGGTCTAGGAGTGGAGGGGAGCCCACGTGCCGGGACTCTGGGGGAGCTTAGCAAGTACT 8200
     ----:----|----:----|----:----|----:----|----:----|----:----|----:----|----:----|----:----|----:----|

       A  A  P  Q  Q  G  L  E  *  G  L  S  Q  G  C  V  L  A  C  F  H  P  A  L  A  L  D  D  A  G  R  *  L   F1
     Q  Q  L  P  N  R  G  W  S  E  A  S  A  R  A  V  F  L  P  V  S  T  Q  P  W  P  *  M  T  Q  G  G  D  S  F2
      S  S  S  P  T  G  A  G  V  R  P  Q  P  G  L  C  S  C  L  F  P  P  S  L  G  L  R  *  R  R  E  V  T    F3
8201 CAGCAGCTCCCCAACAGGGGCTGGAGTGAGGCCTCAGCCAGGGCTGTGTTCTTGCCTGTTTCCACCCAGCCTTGGCCTTAGATGACGCAGGGAGGTGACT 8300
     ----:----|----:----|----:----|----:----|----:----|----:----|----:----|----:----|----:----|----:----|

      R  P  H  L  D  E  P  F  I  P  V  *  V  V  Q  G  P  P  Q  S  Q  P  S  W  A  S  R  P  E  P  Q  A  L    F1
       D  P  I  *  M  N  P  S  Y  Q  S  E  W  C  R  D  L  L  S  H  S  H  H  G  P  A  G  P  S  L  R  L  C   F2
     Q  T  P  S  R  *  T  L  H  T  S  L  S  G  A  G  T  S  S  V  T  A  I  M  G  Q  Q  A  R  A  S  G  S  A  F3
8301 CAGACCCCATCTAGATGAACCCTTCATACCAGTCTGAGTGGTGCAGGGACCTCCTCAGTCACAGCCATCATGGGCCAGCAGGCCCGAGCCTCAGGCTCTG 8400
     ----:----|----:----|----:----|----:----|----:----|----:----|----:----|----:----|----:----|----:----|

     P  C  S  S  S  S  S  G  V  S  S  P  T  G  V  V  P  E  S  P  G  Q  V  A  K  N  E  W  E  G  E  *  G  R  F1
      P  V  P  Q  A  L  Q  V  S  L  P  L  Q  V  W  F  Q  N  R  R  A  K  W  R  K  M  N  G  K  E  S  K  D    F2
       L  F  L  K  L  F  R  C  L  F  P  Y  R  C  G  S  R  I  A  G  P  S  G  E  K  *  M  G  R  R  V  R  T   F3
8401 CCCTGTTCCTCAAGCTCTTCAGGTGTCTCTTCCCCTACAGGTGTGGTTCCAGAATCGCCGGGCCAAGTGGCGAAAAATGAATGGGAAGGAGAGTAAGGAC 8500
     ----:----|----:----|----:----|----:----|----:----|----:----|----:----|----:----|----:----|----:----|

       T  C  R  S  R  P  Y  P  S  P  R  Q  Q  P  M  Q                                                      F1
     A  P  A  G  P  A  L  T  P  A  P  A  S  N  Q  C                                                        F2
      H  L  P  V  P  P  L  P  Q  P  P  P  A  T  N  A                                                       F3
8501 GCACCTGCCGGTCCCGCCCTTACCCCAGCCCCCGCCAGCAACCAATGCAGGTAAGACTTTCTCCTCTTCCTGGCCCCTCCCCATGGGCAGGACATGGGGT 8600
     ----:----|----:----|----:----|----:----|----:----|----:----|----:----|----:----|----:----|----:----|

8601 CTAAGCTGGGATCCTGGGGTCTAGAAGGGGGAAGGTGCTGGAAGGCTGGGCACATGATGGGGCATGTGTGTGTGAGTTGCTTAGCCAAGTTCACCTCTCT 8700
     ----:----|----:----|----:----|----:----|----:----|----:----|----:----|----:----|----:----|----:----|

8701 GCAACCCCAAGGACTGTAACCTACCGGGCTCCTCTGTCCGTGGGGTGTCCCAGGCAAGAATACTGGAACGGGAGGCCATTCCCTTCTCATGGGCTCTTCC 8800
     ----:----|----:----|----:----|----:----|----:----|----:----|----:----|----:----|----:----|----:----|

8801 TGACCCAGGGATCAAACCTGGGTCTCCCACGTCTCAGGTGGCTTCTTTACGGTCTGAGCCAGAGTTGTTGGCTAATATTTAGAGGGATTTGGACCAGGTG 8900
     ----:----|----:----|----:----|----:----|----:----|----:----|----:----|----:----|----:----|----:----|

8901 GGATTGGGACTTGCCAGTGGAGAAGGCAATGGCACCCCACTCCAGTAACTCTTGCCTGGAAAACCCCACAGATGGAGGAGCCTGGTGGGTGTCTGGGTGT 9000
     ----:----|----:----|----:----|----:----|----:----|----:----|----:----|----:----|----:----|----:----|

9001 CTGGTGGGAGCCTGGTGCAGTCCATCGGGTCGCTAAGAGTCGGACACGACTGTGCAACTTCACTTTCACTTTTCACTTTCACACATTGGAGAAGGAAATG 9100
```

```
     ----:----|----:----|----:----|----:----|----:----|----:----|----:----|----:----|----:----|----:----|

9101 GCAACCCACTGTTCTTGCCTTGAGAATCCCAGGGACGGGGGAGCCTGGTGGGCGGCCACCTATGGGGTCGCACAGAGTCGGACACGACTGAAGCGACTTA 9200
     ----:----|----:----|----:----|----:----|----:----|----:----|----:----|----:----|----:----|----:----|

9201 GCAGCAGCAGCAGTGCCTTGTAGGGAGACAGAGCTTGAACGTGTGGGTCCCTTTCCCTACATGGAAGCCCCAGTCATAGACCAGGGACCCCTTGACCTGC 9300
     ----:----|----:----|----:----|----:----|----:----|----:----|----:----|----:----|----:----|----:----|

                              L  C  G  R  A  A  T  Y  R  V  H  D  A  G  A  W  D  P  P  S  G  F  P  S  R     F1
                               S  V  A  E  L  P  P  T  E  S  T  M  L  E  P  G  T  L  P  Q  D  S  L  P  G    F2
                                L  W  P  S  C  H  L  P  S  P  R  C  W  S  L  G  P  S  L  R  I  P  F  Q      F3
9301 AGTCTCTGATCTGTCTGCCACCAGCTCTGTGGCCGAGCTGCCACCTACCGAGTCCACGATGCTGGAGCCTGGGACCCTCCCTCAGGATTCCCTTCCAGGT 9400
     ----:----|----:----|----:----|----:----|----:----|----:----|----:----|----:----|----:----|----:----|

       A  D  P  L  L  R  V  V  W  G  R  R  Q  S  Q  R  A  L  S  W  K  T  W  E  W  P  E  L  S  M  V  L  F    F1
     E  P  T  P  F  *  G  L  S  G  E  D  G  K  V  K  G  L  F  L  G  R  P  G  S  G  Q  S  S  P  W  F  C  S   F2
      S  R  P  P  S  E  G  C  L  G  K  T  A  K  S  K  G  S  F  L  E  D  L  G  V  A  R  A  L  H  G  F  V     F3
9401 GAGCCGACCCCCTTCTGAGGGTTGTCTGGGGAAGACGGCAAAGTCAAAGGGCTCTTTCTTGGAAGACCTGGGAGTGGCCAGAGCTCTCCATGGTTTTGTT 9500
     ----:----|----:----|----:----|----:----|----:----|----:----|----:----|----:----|----:----|----:----|

      S  G  A  T  S  G  S  P  S  L  L  H  S  N  P  L  Q  H  R  Q  G  C  I  P  S  P  L  S  Y  S  H  K  P     F1
       V  E  P  L  R  G  A  Q  A  S  C  T  L  T  P  S  S  T  A  K  A  A  S  P  P  R  C  L  T  L  T  N  P    F2
     Q  W  S  H  F  G  E  P  K  P  L  A  L  *  P  P  P  A  P  P  R  L  H  P  L  P  V  V  L  L  S  Q  T  P   F3
9501 CAGTGGAGCCACTTCGGGGAGCCCAAGCCTCTTGCACTCTAACCCCCTCCAGCACCGCCAAGGCTGCATCCCCTCCCCGTTGTCTTACTCTCACAAACCC 9600
     ----:----|----:----|----:----|----:----|----:----|----:----|----:----|----:----|----:----|----:----|

                             S  R  P  C  C  *  H  L  T  R  L  W  A  Q  T  C  R  M  R  A  P  R  E  G  L  *  P   F1
                              A  V  H  A  A  D  I  *  P  D  S  G  P  K  P  A  E  *  G  P  P  E  K  A  C  D    F2
                               P  S  M  L  L  T  S  D  Q  T  L  G  P  N  L  Q  N  E  G  P  Q  R  R  P  V  T   F3
9601 CCCGTGGACTTCTGTTCCCAGAGCCGTCCATGCTGCTGACATCTGACCAGACTCTGGGCCCAAACCTGCAGAATGAGGGCCCCCAGAGAAGGCCTGTGAC 9700
     ----:----|----:----|----:----|----:----|----:----|----:----|----:----|----:----|----:----|----:----|

       P  H  S  S  A  P  H  L  S  E  E  P  T  F  P  F  P  S  A  L  C  M  P  P  S  *  C  H  C  C  W  T  P    F1
     P  P  T  L  Q  P  P  T  C  P  K  S  Q  P  S  L  S  P  R  P  C  A  C  P  P  A  D  A  T  A  A  G  H  P   F2
      P  P  L  F  S  P  P  P  V  R  R  A  N  L  P  F  P  L  G  P  V  H  A  P  Q  L  M  P  L  L  L  D  T     F3
9701 CCCCCCACTCTTCAGCCCCCCACCTGTCCGAAGAGCCAACCTTCCCTTTCCCCTCGGCCCTGTGCATGCCCCCCAGCTGATGCCACTGCTGCTGGACACC 9800
     ----:----|----:----|----:----|----:----|----:----|----:----|----:----|----:----|----:----|----:----|

      W  A  V  T  A  A  T  R  M  A  L  V  G  C  G  E  Q                                                     F1
       G  Q  *  Q  Q  P  Q  G  W  P  L  W  V  V  G  N  K                                                    F2
     L  G  S  D  S  S  H  K  D  G  P  C  G  L  W  G  T  R                                                   F3
9801 CTGGGCAGTGACAGCAGCCACAAGGATGGCCCTTGTGGGTTGTGGGGAACAAGGTACTTGTCTGCCACTTCACGGGGGGCTGTATTGAGCTATCCTTGGG 9900
     ----:----|----:----|----:----|----:----|----:----|----:----|----:----|----:----|----:----|----:----|
```

```
 9901 GAGGCACAGACGGAGGAAAGATGGATGGAGCTTGGGAGGTGAGAGGGTAAGTGAGAGATGGGGTTTCCCCACCCCCCCAGACAAGAATACTAGAGTGGGT 10000
      ----:----|----:----|----:----|----:----|----:----|----:----|----:----|----:----|----:----|----:----|

10001 TGCAGTTCCATCCTCCAGGGGATCTTCCTGACCCAGGGATCGAACCTGGGTCTCCTGAATTGCAGACAGAATCTACCTTCTGAGCCACCAGGGAAGCCCT 10100
      ----:----|----:----|----:----|----:----|----:----|----:----|----:----|----:----|----:----|----:----|

10101 GAGAGAAGCAAGGAGTTAGTAAATGTAGTGAGGGTTAAAGGGTGAGGGGATGGGGAGGGAAAGGATCAGGACATCACAGCTGTAGAGGAGAAGAGGGTGA 10200
      ----:----|----:----|----:----|----:----|----:----|----:----|----:----|----:----|----:----|----:----|

10201 GAAGCTGGAGCAGGAAGAGGAGGAAGCAGCAGGCCTGGGGTTTGTGGAGAGGCAGGTGGTGGGGGAGGGCGTGGTAGGAATGAAACCATTGGAGCCTCAG 10300
      ----:----|----:----|----:----|----:----|----:----|----:----|----:----|----:----|----:----|----:----|

                             H  H  S  T  P  C  L  L  I  L  R  G  S  G  T  P  G  V  P  T  Q  Q  Q  P  A  R      F1
                              I  T  P  P  P  A  C  S  Y  L  E  D  L  E  L  Q  E  Y  Q  P  S  S  S  Q  P  G    F2
                               S  L  H  P  L  P  A  H  T  *  R  I  W  N  S  R  S  T  N  P  A  A  A  S  Q      F3
10301 GAAGTTCTTCTCTGTCCCCACAGCATCACTCCACCCCCTGCCTGCTCATACTTAGAGGATCTGGAACTCCAGGAGTACCAACCCAGCAGCAGCCAGCCAG 10400
      ----:----|----:----|----:----|----:----|----:----|----:----|----:----|----:----|----:----|----:----|

       I  V  P  L  L  P  G  A  T  D  P  V  L  P  T  P  T  A  P  V  S  I  P  A  P  L  P  F  A  Q  L  A  D      F1
        S  F  P  F  S  Q  G  P  Q  T  Q  F  F  Q  H  P  Q  P  Q  F  P  Y  L  H  P  F  P  L  P  S  S  L  T     F2
      D  R  S  P  S  P  R  G  H  R  P  S  S  S  N  T  H  S  P  S  F  H  T  C  T  P  S  L  C  P  A  R  *  L    F3
10401 GATCGTTCCCCTTCTCCCAGGGGCCACAGACCCAGTTCTTCCAACACCCACAGCCCCAGTTTCCATACCTGCACCCCTTCCCTTTGCCCAGCTCGCTGAC 10500
      ----:----|----:----|----:----|----:----|----:----|----:----|----:----|----:----|----:----|----:----|

      S  T  T  A  R  R  P  S  L  C  L  V  L  W  P  R  R  G  L  I  P  G  L  F  P  R  P  S  V  G  A  R  P  A    F1
       P  P  L  P  E  D  P  L  F  A  L  S  C  G  P  G  G  G  S  S  Q  G  Y  F  P  G  P  P  S  G  P  V  L      F2
        H  H  C  P  K  T  L  S  L  P  C  P  V  A  P  A  G  A  H  P  R  A  I  S  Q  A  L  R  R  G  P  S  C     F3
10501 TCCACCACTGCCCGAAGACCCTCTCTTTGCCTTGTCCTGTGGCCCCGGCGGGGGCTCATCCCAGGGCTATTTCCCAGGCCCTCCGTCGGGGCCCGTCCTG 10600
      ----:----|----:----|----:----|----:----|----:----|----:----|----:----|----:----|----:----|----:----|

        A  A  A  G  R  E  C                                                                                  F1
      L  Q  P  P  A  G  N  V                                                                                 F2
       C  S  R  R  P  G  M  W                                                                                F3
10601 CTGCAGCCGCCGGCCGGGAATGTGGGTGAGTCGGGGCTGGAAGAGAAGGCCCCCCCGACCCCAGTGTTTGAGGAGGGTGAGAGGGGAGAGATCCCCAAGA 10700
      ----:----|----:----|----:----|----:----|----:----|----:----|----:----|----:----|----:----|----:----|

10701 CGGGGGCTATCTTGGTGAAGCTCTTCGTCCTCCAGTTTCCTGGAGGTTTCAGAGCAGAGTAAGAGAGAGCTTTGGGTGTGTGTTAGTTGCTTAGTCGTGT 10800
      ----:----|----:----|----:----|----:----|----:----|----:----|----:----|----:----|----:----|----:----|

10801 CCGACTCTTTGCAACCCCAGAGACTGTAGCCCACCAGGCCCCTCCATCCATGGGATTCTCCAGGCAAGAACACTGGAGTGGGTTACCATTTCCGTCTCCA 10900
      ----:----|----:----|----:----|----:----|----:----|----:----|----:----|----:----|----:----|----:----|

10901 AAAGGAACTATAGAAAGAAAGAAAGTGAAGTCGCTCAGTCGTGTCCGACTCTTTGCGACCCCATAGACTGTAGCCTACCAGGTTCCTCCATCCATGGAGT 11000
      ----:----|----:----|----:----|----:----|----:----|----:----|----:----|----:----|----:----|----:----|
```

```
11001 TGTCCAGGCAAGAGTACTGGAGTGGGTTGCCATTTCCTTCTCCGAGAGAGAGAGCTTTGGAGGGAGGCAAAGCCAAGCATGGAGTCCAGCTCAGTCATCA 11100
      ----:----|----:----|----:----|----:----|----:----|----:----|----:----|----:----|----:----|----:----|

11101 CTAGTTCTGAGCTTCCTGGATTCTGAAGTGGGAATAGTATTGCCATCTCTTTGAGGGCTTGGTGAGGACTAGGAATGACGGGCGTGAAACACTCGGTGCA 11200
      ----:----|----:----|----:----|----:----|----:----|----:----|----:----|----:----|----:----|----:----|

11201 GAGTAGATGGCAGCTAAAGGGAGTGTCCAAGGGGCCACCGGGACCCCGGATATGCTGGAGTTATCGCCCTAGGAGACTCAGGAGAAGAAGAGGGAGAAAG 11300
      ----:----|----:----|----:----|----:----|----:----|----:----|----:----|----:----|----:----|----:----|

11301 ACTCTGGTTCCCGGGCTCCTTCCACATGTGTGAGGGGGCCCCAGCCCGACCTGCAGTGTGCTCTGGGTCGCTGTGCCCCAACCCCAGGCCCTTGAGTCAT 11400
      ----:----|----:----|----:----|----:----|----:----|----:----|----:----|----:----|----:----|----:----|

11401 GGGGGGCGGGTGCCTGGTCCTGAGGGGAGACCCACTCTGGGGTCCTGACCCGTGTCTCTGTGGAGCAGGGAGGGTCTCTGTCGCGGAGACTGCCCTGGGG 11500
      ----:----|----:----|----:----|----:----|----:----|----:----|----:----|----:----|----:----|----:----|

                                            P  T  G  T  V  P  W  A  D  P  C  L  P  D  L  P  F  P  S  A  F     F1
                                          A  P  Q  V  R  C  P  G  P  T  P  A  C  Q  T  C  H  S  P  V  P  S    F2
                                           P  H  R  Y  G  A  L  G  R  P  L  P  A  R  L  A  I  P  Q  C  L      F3
11501 CCCCTTCATGCCGTGCGCTCTGTGCTGTGTGCTTTGGCCCCACAGGTACGGTGCCCTGGGCCGACCCCTGCCTGCCAGACTTGCCATTCCCCAGTGCCTT 11600
      ----:----|----:----|----:----|----:----|----:----|----:----|----:----|----:----|----:----|----:----|

       C  P  Q  S  L  G  G  P  P  G  G  D  G  C  F  L  D  L  F  A  A  P  Y  A  Q  A  S  G  R  L  P  S  P      F1
        A  R  S  L  W  G  A  P  R  E  G  T  A  A  S  W  T  C  L  L  P  P  T  H  R  L  Q  V  G  C  L  P  R     F2
      L  P  A  V  S  G  G  P  P  G  R  G  R  L  L  P  G  P  V  C  C  P  L  R  T  G  F  R  *  A  A  F  P  G    F3
11601 CTGCCCGCAGTCTCTGGGGGGCCCCCCGGGAGGGGACGGCTGCTTCCTGGACCTGTTTGCTGCCCCCTACGCACAGGCTTCAGGTAGGCTGCCTTCCCCG 11700
      ----:----|----:----|----:----|----:----|----:----|----:----|----:----|----:----|----:----|----:----|

      G  L  T  Q  M  P  E  S  T  P  P  A  A  G  K  A  P  L  S  Q  A  Q  E  E  P  P  A  A  P  G  E  R  P  P    F1
       A  S  P  R  C  P  R  A  P  H  L  Q  Q  E  K  P  R  S  A  R  P  R  R  N  H  R  L  P  P  G  S  G  P      F2
        P  H  P  D  A  R  E  H  P  T  C  S  R  K  S  P  A  Q  P  G  P  G  G  T  T  G  C  P  R  G  A  A  P     F3
11701 GGCCTCACCCAGATGCCCGAGAGCACCCCACCTGCAGCAGGAAAAGCCCCGCTCAGCCAGGCCCAGGAGGAACCACCGGCTGCCCCCGGGGAGCGGCCCC 11800
      ----:----|----:----|----:----|----:----|----:----|----:----|----:----|----:----|----:----|----:----|

        A  P  E  E  E  D  K  S  G  H  G  P  *  S  W  S  R  R  T  K  R  P  S  C  R  R  W  L  E  H  P  E  E     F1
      R  P  P  R  K  K  T  R  V  A  M  A  P  S  H  G  A  E  E  P  R  G  R  P  A  G  D  G  W  S  T  Q  R  R    F2
       G  P  R  G  R  R  Q  E  W  P  W  P  L  V  M  E  P  K  N  Q  E  A  V  L  P  E  M  A  G  A  P  R  G      F3
11801 CGGCCCCCGAGGAAGAAGACAAGAGTGGCCATGGCCCCTAGTCATGGAGCCGAAGAACCAAGAGGCCGTCCTGCCGGAGATGGCTGGAGCACCCAGAGGA 11900
      ----:----|----:----|----:----|----:----|----:----|----:----|----:----|----:----|----:----|----:----|

       T  G  L  E  V  E  G  K  T  G  L  Q  G  S  F  C  Q  M  L  T  *  G  G  G  G  L  D  H  P  S  F  P  H      F1
        Q  G  S  K  L  K  E  R  L  D  S  R  A  H  F  A  K  C  *  L  E  E  E  E  A  W  T  T  R  P  S  P  T     F2
      D  R  A  R  S  *  R  K  D  W  T  P  G  L  I  L  P  N  A  D  L  R  R  R  R  P  G  P  P  V  L  P  P  Q    F3
11901 GACAGGGCTCGAAGTTGAAGGAAAGACTGGACTCCAGGGCTCATTTTGCCAAATGCTGACTTGAGGAGGAGGAGGCCTGGACCACCCGTCCTTCCCCCAC 12000
```

```
      ----:----|----:----|----:----|----:----|----:----|----:----|----:----|----:----|----:----|----:----|
      S  S  E  W  W  S  W  R  N  G  V  G  P  C  R  L  *  R  L  L  P  F  M  L  F  A  L  L  A  P  Q  P  L  Q   F1
       A  P  N  G  G  P  G  E  M  E  W  G  R  V  A  S  D  G  C  F  R  L  C  Y  L  L  S  L  L  H  S  R  S    F2
        L  R  M  V  V  L  E  K  W  S  G  A  V  S  P  L  T  V  A  S  V  Y  V  I  C  S  P  C  S  T  A  A  P   F3
12001 AGCTCCGAATGGTGGTCCTGGAGAAATGGAGTGGGGCCGTGTCGCCTCTGACGGTTGCTTCCGTTTATGTTATTTGCTCTCCTTGCTCCACAGCCGCTCC 12100
      ----:----|----:----|----:----|----:----|----:----|----:----|----:----|----:----|----:----|----:----|
                                                                                   Cloning primer <---------

        W  P  P  *  A  V  N  C  L  L  L  G  F  S  L  C  S  D  V  S  P  F  S  R  *  *  R  P  P  L  P  F  I   F1
      S  G  H  H  K  Q  *  T  A  S  S  W  V  S  L  S  V  V  M  C  L  P  S  H  A  S  E  D  P  P  C  P  S  L  F2
       V  A  T  I  S  S  K  L  P  P  L  G  F  L  S  L  *  *  C  V  S  L  L  T  L  V  K  T  P  P  A  L  H    F3
12101 AGTGGCCACCATAAGCAGTAAACTGCCTCCTCTTGGGTTTCTCTCTCTGTAGTGATGTGTCTCCCTTCTCACGCTAGTGAAGACCCCCCCTGCCCTTCAT 12200
      ----:----|----:----|----:----|----:----|----:----|----:----|----:----|----:----|----:----|----:----|

       A  T  H  S  A  G  Q  L  C  T  E  R  L  R  R  D  *  P  S  G  A  G  S  C  E  P  G  I  L  L  G  P  I    F1
        P  P  T  Q  L  G  S  C  V  L  R  G  S  G  G  T  D  P  L  G  Q  D  R  V  S  L  A  S  F  W  A  P  S   F2
      C  H  P  L  S  W  A  A  V  Y  *  E  A  Q  E  G  L  T  L  W  G  R  I  V  *  A  W  H  P  S  G  P  H  L  F3
12201 TGCCACCCACTCAGCTGGGCAGCTGTGTACTGAGAGGCTCAGGAGGGACTGACCCTCTGGGGCAGGATCGTGTGAGCCTGGCATCCTTCTGGGCCCCATC 12300
      ----:----|----:----|----:----|----:----|----:----|----:----|----:----|----:----|----:----|----:----|

      S  P  A  G  Q  G  M  G  P  P  G  P  C  L  *  S  Y  L  *  T  M  F  S  S  Q  T  Q  K  *  A  *  T  N  L  F1
       L  L  L  G  K  E  W  G  L  L  G  L  V  F  S  P  I  F  K  P  C  S  R  L  K  P  R  S  E  P  E  Q  T    F2
        S  C  W  A  R  N  G  A  S  W  A  L  S  L  V  L  S  L  N  H  V  L  V  S  N  P  E  V  S  L  N  K  P   F3
12301 TCTCCTGCTGGGCAAGGAATGGGGCCTCCTGGGCCTTGTCTTTAGTCCTATCTTTAAACCATGTTCTCGTCTCAAACCCAGAAGTGAGCCTGAACAAACC 12400
      ----:----|----:----|----:----|----:----|----:----|----:----|----:----|----:----|----:----|----:----|

        S  L  G  L  F  L  Q  F  G  P  C  R  D  G  S  S  Q  P  V  K  N  L  P  A  R  Q  E  T  G  V  *  S  L   F1
      *  A  W  D  F  F  C  N  L  G  P  A  G  M  E  A  L  S  P  *  R  I  C  L  Q  G  R  R  P  G  F  D  P  W  F2
       E  P  G  T  F  S  A  I  W  A  L  Q  G  W  K  L  S  A  R  K  E  S  A  C  K  A  G  D  R  G  L  I  P    F3
12401 TGAGCCTGGGACTTTTTCTGCAATTTGGGCCCTGCAGGGATGGAAGCTCTCAGCCCGTAAAGAATCTGCCTGCAAGGCAGGAGACCGGGGTTTGATCCCT 12500
      ----:----|----:----|----:----|----:----|----:----|----:----|----:----|----:----|----:----|----:----|

       G  R  E  D  F  P  Q  R  R  E  W  Q  P  T  P  V  F  L  P  G  E  S  H  G  Q  R  S  L  V  G  Y  S  P    F1
        V  G  K  I  S  P  R  E  G  N  G  N  P  L  Q  Y  S  C  L  E  N  P  M  D  R  G  A  W  W  A  T  V  Q   F2
      G  *  G  R  F  P  P  E  K  G  M  A  T  H  S  S  I  L  A  W  R  I  P  W  T  E  E  P  G  G  L  Q  S  R  F3
12501 GGGTAGGGAAGATTTCCCCCAGAGAAGGGAATGGCAACCCACTCCAGTATTCTTGCCTGGAGAATCCCATGGACAGAGGAGCCTGGTGGGCTACAGTCCA 12600
      ----:----|----:----|----:----|----:----|----:----|----:----|----:----|----:----|----:----|----:----|

      G  I  C  K  V  L  D  M  T  D  A  T  N  S  *  L  G  I  G  *  *  E  K  L  K  A  V  *  A  K  S  L  E  L  F1
       E  F  A  K  C  W  T  *  L  M  Q  L  T  L  N  *  G  *  D  D  E  R  S  S  R  L  F  R  P  S  L  *  N    F2
        N  L  Q  S  V  G  H  D  *  C  N  *  L  L  T  R  D  R  M  M  R  E  A  Q  G  C  L  G  Q  V  S  R  I   F3
12601 GGAATTTGCAAAGTGTTGGACATGACTGATGCAACTAACTCTTAACTAGGGATAGGATGATGAGAGAAGCTCAAGGCTGTTTAGGCCAAGTCTCTAGAAT 12700
```

```
      ----:----|----:----|----:----|----:----|----:----|----:----|----:----|----:----|----:----|----:----|

        *  T  M  I  Y  F  P  K  A  L  L  F  L  G  *  E  G  I  G  *  E  *  G  V  G  R  P  R  R  G  C  S  Y    F1
      C  E  P  *  F  I  F  P  K  L  F  F  F  W  V  E  K  G  L  G  R  N  E  G  W  E  D  Q  E  E  D  A  L  I   F2
       V  N  H  D  L  F  S  Q  S  S  S  F  S  G  L  R  R  D  W  V  G  M  R  G  G  K  T  K  K  R  M  L  L     F3
12701 TGTGAACCATGATTTATTTTCCCAAAGCTCTTCTTTTTCTGGGTTGAGAAGGGATTGGGTAGGAATGAGGGGTGGGAAGACCAAGAAGAGGATGCTCTTA 12800
      ----:----|----:----|----:----|----:----|----:----|----:----|----:----|----:----|----:----|----:----|

       L  F  I  Y  I  Y  I  Y  L  C  I  Y  I  *  Y  P  H  L  I  T  L  Q  A  S  L  V  A  Q  T  I  K  N  L     F1
        Y  L  Y  I  Y  I  Y  I  Y  V  Y  I  F  S  T  H  I  *  *  P  C  R  L  P  *  W  L  R  Q  *  R  I  C    F2
      F  I  Y  I  Y  I  Y  I  F  M  Y  I  Y  L  V  P  T  S  D  N  P  A  G  F  P  S  G  S  D  N  K  E  S  A   F3
12801 TTTATTTATATATATATATATATATTTATGTATATATATTTAGTACCCACATCTGATAACCCTGCAGGCTTCCCTAGTGGCTCAGACAATAAAGAATCTG 12900
      ----:----|----:----|----:----|----:----|----:----|----:----|----:----|----:----|----:----|----:----|

      P  E  M  Q  E  M  Q  V  R  S  L  G  Q  K  D  P  L  E  K  G  M  A  T  H  S  S  V  L  A  W  R  C  P  W   F1
       L  K  C  R  R  C  R  F  D  P  W  V  R  K  I  P  W  R  R  E  W  Q  P  I  L  V  F  L  P  G  D  V  H     F2
        *  N  A  G  D  A  G  S  I  P  G  S  E  R  S  P  G  E  G  N  G  N  P  F  *  C  S  C  L  E  M  S  M    F3
12901 CCTGAAATGCAGGAGATGCAGGTTCGATCCCTGGGTCAGAAAGATCCCCTGGAGAAGGGAATGGCAACCCATTCTAGTGTTCTTGCCTGGAGATGTCCAT 13000
      ----:----|----:----|----:----|----:----|----:----|----:----|----:----|----:----|----:----|----:----|

        T  E  E  P  G  R  L  Q  S  M  E  S  Q  R  V  G  H  N  *  A  T  S  T  H  T  S  D  D  P  T  V  S  K    F1
      G  Q  R  S  L  A  G  Y  S  L  W  S  R  K  E  L  D  T  T  E  Q  R  A  H  T  L  L  M  T  L  Q  C  P  N   F2
       D  R  G  A  W  Q  A  T  V  Y  G  V  A  K  S  W  T  Q  L  S  N  E  H  T  H  F  *  *  P  Y  S  V  Q     F3
13001 GGACAGAGGAGCCTGGCAGGCTACAGTCTATGGAGTCGCAAAGAGTTGGACACAACTGAGCAACGAGCACACACACTTCTGATGACCCTACAGTGTCCAA 13100
      ----:----|----:----|----:----|----:----|----:----|----:----|----:----|----:----|----:----|----:----|

       *  L  E  F  Q  G  *  *  L  K  P  P  C  T  *  V  Y  M  G  C  H  S  G  P  P  M  L  *  L  T  Y  I  H     F1
        N  *  N  F  R  A  N  D  S  N  H  H  A  L  E  Y  T  W  G  V  I  L  D  L  Q  C  Y  D  *  L  I  S  I    F2
      I  T  R  I  S  G  L  M  T  Q  T  T  M  H  L  S  I  H  G  V  S  F  W  T  S  N  A  M  I  N  L  Y  P  S   F3
13101 ATAACTAGAATTTCAGGGCTAATGACTCAAACCACCATGCACTTGAGTATACATGGGGTGTCATTCTGGACCTCCAATGCTATGATTAACTTATATCCAT 13200
      ----:----|----:----|----:----|----:----|----:----|----:----|----:----|----:----|----:----|----:----|

      H  T  R  S  R  V  M  A  H  K  N  *  K  G  K  V  L  R  N  G  E  E  S  P  *  L  H  K  H  M  S  G  Y  L   F1
       I  L  D  L  G  S  W  P  I  R  T  E  K  G  K  Y  *  E  M  E  R  N  H  L  S  Y  T  N  T  C  L  G  I     F2
        Y  *  I  *  G  H  G  P  *  E  L  K  R  E  S  I  E  K  W  R  G  I  T  L  A  T  Q  T  H  V  W  V  S    F3
13201 CATACTAGATCTAGGGTCATGGCCCATAAGAACTGAAAAGGGAAAGTATTGAGAAATGGAGAGGAATCACCTTAGCTACACAAACACATGTCTGGGTATC 13300
      ----:----|----:----|----:----|----:----|----:----|----:----|----:----|----:----|----:----|----:----|

        C  R  V  L  K  K  F  H  G  Q  R  T  L  V  A  I  V  H  V  V  T  R  E  S  D  T  T  W  Q  L  N  N  C    F1
      C  V  G  F  *  R  N  S  M  D  R  G  P  W  W  L  *  S  T  W  L  Q  E  S  Q  T  R  L  G  N  *  T  I  V   F2
       V  *  G  F  K  E  I  P  W  T  E  D  P  G  G  Y  S  P  R  G  Y  K  R  V  R  H  D  L  A  T  K  Q  L     F3
13301 TGTGTAGGGTTTTAAAGAAATTCCATGGACAGAGGACCCTGGTGGCTATAGTCCACGTGGTTACAAGAGAGTCAGACACGACTTGGCAACTAAACAATTG 13400
      ----:----|----:----|----:----|----:----|----:----|----:----|----:----|----:----|----:----|----:----|
```

```
       D  V  T  G  S  F  K  I  Y  L  S  F  A  F  T  F  V  S  *  H  P  *  N  D  Q  A  K  N  S  V  R  G  K    F1
        M  *  L  G  L  L  K  Y  T  S  L  L  L  S  H  L  S  H  D  T  H  K  M  T  R  L  K  T  V  *  E  E  K  F2
      *  C  N  W  V  F  *  N  I  P  L  F  C  F  H  I  C  L  M  T  P  I  K  *  P  G  *  K  Q  C  K  R  K  K F3
13401 TGATGTAACTGGGTCTTTTAAAATATACCTCTCTTTTGCTTTCACATTTGTCTCATGACACCCATAAAATGACCAGGCTAAAAACAGTGTAAGAGGAAAA 13500
      ----:----|----:----|----:----|----:----|----:----|----:----|----:----|----:----|----:----|----:----|

      N  Y  S  T  *  G  V  E  D  S  N  C  S  I  P  C  C  F  S  S  L  A  I  S  Y  H  K  I  L  L  L  S  K  P F1
       I  I  L  H  K  G  *  K  T  V  T  V  Q  F  H  A  V  F  P  L  L  P  S  V  T  T  R  Y  C  C  F  Q  S   F2
        L  F  Y  I  R  G  R  R  Q  *  L  F  N  S  M  L  F  F  L  S  C  H  Q  L  P  Q  D  T  A  A  F  K  A  F3
13501 AATTATTCTACATAAGGGGTAGAAGACAGTAACTGTTCAATTCCATGCTGTTTTTCCTCTCTTGCCATCAGTTACCACAAGATACTGCTGCTTTCAAAGC 13600
      ----:----|----:----|----:----|----:----|----:----|----:----|----:----|----:----|----:----|----:----|

        A  K  R  L  D  I  F  S  S  R  S  L  R  I  *  A  P  V  A  Q  M  R  K  H  F  P  G  G  S  V  V  K  N  F1
      L  Q  R  G  W  T  F  S  R  P  D  L  Y  G  F  R  L  L  S  L  R  *  G  S  T  S  L  V  A  Q  W  *  R  T F2
       C  K  E  A  G  H  F  L  V  Q  I  F  T  D  L  G  S  C  R  S  D  E  E  A  L  P  W  W  L  S  G  K  E   F3
13601 CTGCAAAGAGGCTGGACATTTTCTCGTCCAGATCTTTACGGATTTAGGCTCCTGTCGCTCAGATGAGGAAGCACTTCCCTGGTGGCTCAGTGGTAAAGAA 13700
      ----:----|----:----|----:----|----:----|----:----|----:----|----:----|----:----|----:----|----:----|

       L  H  G  N  A  G  D  I  G  S  L  P  G  S  G  R  S  P  E  E  G  N  G  N  L  L  Q  Y  S  C  L  G  N   F1
        C  M  A  M  Q  E  I  *  V  P  F  L  G  L  E  D  P  L  K  K  E  M  A  T  Y  S  S  I  L  A  W  E  I  F2
      P  A  W  Q  C  R  R  Y  R  F  P  S  W  V  W  K  I  P  *  R  R  K  W  Q  P  T  P  V  F  L  P  G  K  S F3
13701 CCTGCATGGCAATGCAGGAGATATAGGTTCCCTTCCTGGGTCTGGAAGATCCCCTGAAGAAGGAAATGGCAACCTACTCCAGTATTCTTGCCTGGGAAAT 13800
      ----:----|----:----|----:----|----:----|----:----|----:----|----:----|----:----|----:----|----:----|

      P  M  D  R  G  T  P  W  T  M  V  H  S  S  *  K  S  W  A  Q  L  S  D  *  M  M  T  T  T  *  I  S  N  A F1
       P  W  T  E  E  P  R  G  L  W  S  T  A  H  E  R  V  G  H  N  L  A  T  K  *  *  Q  Q  L  R  *  V  M   F2
        H  G  Q  R  N  P  V  D  Y  G  P  Q  L  M  K  E  L  G  T  T  *  R  L  N  D  D  N  N  L  D  K  *  C  F3
13801 CCCATGGACAGAGGAACCCCGTGGACTATGGTCCACAGCTCATGAAAGAGTTGGGCACAACTTAGCGACTAAATGATGACAACAACTTAGATAAGTAATG 13900
      ----:----|----:----|----:----|----:----|----:----|----:----|----:----|----:----|----:----|----:----|

        L  Y  F  *  Q  T  V  D  I  *  S  Q  E  L  E  G  D  G  L  *  D  L  C  L  Y  T  K  S  G  N  L  I  V  F1
      P  C  T  F  S  K  Q  *  I  S  K  A  K  N  W  R  V  M  G  F  K  I  C  A  Y  T  Q  S  Q  A  I  *  *  Y F2
       P  V  L  L  A  N  S  R  Y  L  K  P  R  T  G  G  *  W  A  L  R  S  V  L  I  H  K  V  R  Q  S  N  S   F3
13901 CCCTGTACTTTTAGCAAACAGTAGATATCTAAAGCCAAGAACTGGAGGGTGATGGGCTTTAAGATCTGTGCTTATACACAAAGTCAGGCAATCTAATAGT 14000
      ----:----|----:----|----:----|----:----|----:----|----:----|----:----|----:----|----:----|----:----|

       Y  *  K  Q  S  V  *  I  E  N  E  *  K  I  K  P  R  E  N  G  E  N  I  L  E  A  Q  N  P  G  L  T  R   F1
        T  E  N  K  V  C  E  L  R  M  N  E  R  S  N  Q  G  K  M  V  K  T  F  *  K  H  R  I  L  G  *  H  V  F2
      I  L  K  T  K  C  V  N  *  E  *  M  K  D  Q  T  K  G  K  W  *  K  H  F  R  S  T  E  S  W  A  D  T  S F3
14001 ATACTGAAAACAAAGTGTGTGAATTGAGAATGAATGAAAGATCAAACCAAGGGAAAATGGTGAAAACATTTTAGAAGCACAGAATCCTGGGCTGACACGT 14100
      ----:----|----:----|----:----|----:----|----:----|----:----|----:----|----:----|----:----|----:----|

      P  T  S  W  Q  M  Q  E  *  A  L  H  H  P  *  W  S  I  I  H  S  *  L  S  S  G  A  A  G  L  R  G  S  * F1
       Q  P  A  G  R  C  R  N  E  L  Y  T  T  P  D  G  A  S  F  I  L  N  F  P  V  G  L  Q  G  *  G  G  A   F2
```

```
        N  Q  L  A  D  A  G  M  S  S  T  P  P  L  M  E  H  H  S  F  L  T  F  Q  W  G  C  R  V  E  G  E  L    F3
14101 CCAACCAGCTGGCAGATGCAGGAATGAGCTCTACACCACCCCTGATGGAGCATCATTCATTCTTAACTTTCCAGTGGGGCTGCAGGGTTGAGGGGGAGCT 14200
      ----:----|----:----|----:----|----:----|----:----|----:----|----:----|----:----|----:----|----:----|

        L  L  Y  I  R  A  Y  R  M  L  K  A  L  V  V  M  K  F  F  F  *  E  E  V  N  I  V  *  L  N  L  S  F    F1
      N  S  F  T  *  E  P  I  E  C  *  K  L  *  L  *  *  S  S  S  S  R  R  K  S  T  L  F  N  *  I  *  V  L   F2
       T  P  L  H  K  S  L  *  N  V  E  S  F  S  C  N  E  V  L  L  L  G  G  S  Q  H  C  L  T  E  F  E  F     F3
14201 AACTCCTTTACATAAGAGCCTATAGAATGTTGAAAGCTTTAGTTGTAATGAAGTTCTTCTTCTAGGAGGAAGTCAACATTGTTTAACTGAATTTGAGTTT 14300
      ----:----|----:----|----:----|----:----|----:----|----:----|----:----|----:----|----:----|----:----|

       A  L  H  C  A  T  K  N  K  P  S  F  T  R  S  E  D  C  V  A  F  L  R  A  L  A  S  R  G  L  V  S  L     F1
        L  C  I  V  L  R  R  I  N  L  V  S  Q  D  L  R  T  V  W  H  S  S  E  P  W  P  Q  G  A  W  S  H  S    F2
      C  F  A  L  C  Y  E  E  *  T  *  F  H  K  I  *  G  L  C  G  I  P  Q  S  P  G  L  K  G  P  G  L  T  L   F3
14301 TGCTTTGCATTGTGCTACGAAGAATAAACCTAGTTTCACA                                                             14400
      ----:----|----:----|----:----|----:----|
```
