# Supplementary material for: Molecular cloning of PRD-like homeobox genes expressed in bovine oocytes and early IVF embryos
Source: BMC Genomics. 2024 Nov 6;25:1048. doi: 10.1186/s12864-024-10969-w (PMC11542365; doi:10.1186/s12864-024-10969-w)
Supplement: Supplementary file 8 — Supplementary Material 8: Additional file 14: Figure S6. The prediction of TPRX1 derived from Bos taurus isolate L1 Dominette 01449 registration number 42190680 breed Hereford chromosome 18, ARS-UCD1.2, whole genome shotgun sequence. Three possible ORFs for exons, but not introns, are depicted. Putative protein sequence is highlighted in yellow. Sequences from StringTie merge prediction and confirmed cDNA are drawn as lines below the corresponding sequences. Cloning primers are drawn as line arrows. Splice sites are underlined and codons split by two exons are coloured red. The homeodomain is highlighted in green. [file 12864_2024_10969_MOESM8_ESM.pdf]

Supplementary Figure S6. The prediction of *TPRX1* derived from *Bos taurus* isolate L1 Dominette 01449 registration number 42190680 breed Hereford chromosome 18, ARS-UCD1.2, whole genome shotgun sequence. Three possible ORFs for exons, but not introns, are depicted. Putative protein sequence is highlighted in yellow. Sequences from StringTie merge prediction and confirmed cDNA are drawn as lines below the corresponding sequences. Cloning primers are drawn as line arrows. Splice sites are underlined and codons split by two exons are coloured red. The homeodomain is highlighted in green.

```
                                                                            A  G  G  C  K  N  P  S  P  S  F1
                                                                          Q  Q  E  D  A  R  T  R  V  R  H F2
                                                                           S  R  R  M  Q  E  P  E  S  V   F3
4901                                                                    AACAGCAGGAGGATGCAAGAACCCGAGTCCGTC 5000
                                                                          |----:----|----:----|----:----|
                                                    Cloning primer
                                                    TPRX1_16c_01

      *                                                                                                   F1
       E                                                                                                  F2
     M  S                                                                                                 F3
5001 ATGAGTGAGTCTGGCCTCCTGGGACTTAGTGGAGTTTGGGGGTGGAATCACATTACTAGGGGAGAGGAGCAGAGAGCCTGGTCCTCGGGGGTCCCCTGTC 5100
     ----:----|----:----|----:----|----:----|----:----|----:----|----:----|----:----|----:----|----:----|
StringTie merge

5101 GGGGTGGCTCCCCCCTTGGTTGGGAGATAGTGGGGGTTGGGGAGAGCACCAGGGGTCACCAAGGAGCCTGTCCTGCGTGCCTTCCCAGGGCAGGGCCCTG 5200
     ----:----|----:----|----:----|----:----|----:----|----:----|----:----|----:----|----:----|----:----|

5201 GAATAAATACTCCATGGGCTGAGATCAAGCCAATTTCCAAGCTGTGCTCACTGAGGCCCAGAGAGGTGCAGTGGTTAGGGCTGGAGGGCGGACAGCCAGG 5300
     ----:----|----:----|----:----|----:----|----:----|----:----|----:----|----:----|----:----|----:----|

5301 GTCTGACCAACAACCTCACTCCAGATGCCCCCAGCTTCTCCTCCCGCCCTCGGGCCTGAGTCCCACCGGCTCACCAGGGTCTGGTCCAGGGAGGGGGAGG 5400
     ----:----|----:----|----:----|----:----|----:----|----:----|----:----|----:----|----:----|----:----|
                                                                 P  H  V  E  V  S  A  P  K  E  K  A  A  P F1
                                                                  P  T  W  R  S  A  P  P  K  R  K  R  R   F2
                                                                A  P  R  G  G  Q  R  P  Q  R  E  S  G  A  F3
5401 GCGCCAGGCAGGCTGGGGGCTATTCCTGGGCTCATGGGCCTTGTTTCCCCCTTGGTCAGGCCCCACGTGGAGGTCAGCGCCCCCAAAGAGAAAGCGGCGC 5500
     ----:----|----:----|----:----|----:----|----:----|----:----|----:----|----:----|----:----|----:----|

       A  H  G  V  Q  Q  R  A  V  G  G  A  Q  R  S  L  P  E  E  R  V  P  E  L  P  G  P  P  A  P  G  G  Q  F1
     Q  R  T  V  Y  S  K  E  Q  L  E  A  L  K  E  A  F  L  K  N  E  Y  P  S  Y  Q  D  R  L  R  L  A  A  R F2
      S  A  R  C  T  A  K  S  S  W  R  R  S  K  K  P  S  *  R  T  S  T  R  V  T  R  T  A  C  A  W  R  P   F3
5501 CAGCGCACGGTGTACAGCAAAGAGCAGTTGGAGGCGCTCAAAGAAGCCTTCCTGAAGAACGAGTACCCGAGTTACCAGGACCGCCTGCGCCTGGCGGCCA 5600
     ----:----|----:----|----:----|----:----|----:----|----:----|----:----|----:----|----:----|----:----|
```

```
      A   P   P   G   R   T   Q   S   A                                                                  F1
       L   H   L   D   E   H   R   V   Q                                                                 F2
     G   S   T   W   T   N   T   E   C                                                                   F3
5601 GGCTCCACCTGGACGAACACAGAGTGCAGGTCTGAGCCCGCCGCCCCGGGGCCACCCCTCCGCCCTCCCAGAGCCACCTCTGGGGACCGCGAGGCCAGAG 5700
     ----:----|----:----|----:----|----:----|----:----|----:----|----:----|----:----|----:----|----:----|

5701 GGGCCAGGGCTGCCCCGGGTGCCGCAGCAAGCAAGGGGTCTCCCCGCTGTGGACCCAGGGCTGGACCCCTTCCGCCTCAACCCTGTGCTGTATCACCCCG 5800
     ----:----|----:----|----:----|----:----|----:----|----:----|----:----|----:----|----:----|----:----|

5801 CCAACCGCATGCGGCCTGCCTGACACCCCCCATCGCCCCGGGGTGTCCCGGTGCCCAGCCCTCAGGGCCTCTGACTCCGCGGACCCTCTCGGGCAGACCC 5900
     ----:----|----:----|----:----|----:----|----:----|----:----|----:----|----:----|----:----|----:----|

5901 CTTCCTGCCCCGTGGGCGCCCAAGCCCGACCCCCTGCCCTCTGCACCCCTTCCCGCTGCCTCCTGAACCCCAGGAGCCTCTAATTGGGTCTGGGGGGCGG 6000
     ----:----|----:----|----:----|----:----|----:----|----:----|----:----|----:----|----:----|----:----|

6001 AGGGAGAGGGGAGGCGGGATTCTCAGCTGGACAGCCTCACCCCCGGAGCAGAGGCGATAGCGCGGGGCCCCTCGCCTCTCACACCCGCTTCTCCCGGTCC 6100
     ----:----|----:----|----:----|----:----|----:----|----:----|----:----|----:----|----:----|----:----|

                    V   V   Q   E   P   P   G   P   A   L   P   S   G   A   T   T   G   P   G   R   I   P   D   G   R   R   C   A   F1
                   V   W   F   K   N   R   R   A   Q   R   S   R   L   E   R   R   Q   A   Q   G   G   Y   Q   M   A   G   D   A   P  F2
                     C   G   S   R   T   A   G   P   S   A   P   V   W   S   D   D   R   P   R   E   D   T   R   W   P   A   M   R   F3
6101 CCTCTGCCCACCCAGGTGTGGTTCAAGAACCGCCGGGCCCAGCGCTCCCGTCTGGAGCGACGACAGGCCCAGGGAGGATACCAGATGGCCGGCGATGCGC 6200
     ----:----|----:----|----:----|----:----|----:----|----:----|----:----|----:----|----:----|----:----|

      H   G   P   R   S   P   R   D   S   R   P   R   P   G   I   C   C   R   S   C   E   P   Q   F   P   R   Q   P   G   I   L   Q   P   F1
       T   D   P   G   A   P   G   T   P   A   P   A   P   E   S   A   A   A   A   A   N   P   N   F   P   D   S   P   G   F   Y   S   R  F2
     P   R   T   P   E   P   P   G   L   P   P   P   P   R   N   L   L   P   Q   L   R   T   P   I   S   Q   T   A   R   D   S   T   A   A F3
6201 CCACGGACCCCGGAGCCCCCGGGACTCCCGCCCCCGCCCCGGAATCTGCTGCCGCAGCTGCGAACCCCAATTTCCCAGACAGCCCGGGATTCTACAGCCG 6300
     ----:----|----:----|----:----|----:----|----:----|----:----|----:----|----:----|----:----|----:----|

     P   S   S   P   Q   P   R   G   G   A   P   S   A   R   A   W   H   L   Q   P   P   P   G   H   V   G   P   G   T   G   R   P   R   V F1
      P   P   P   P   S   P   A   G   V   L   P   A   P   E   P   G   I   S   S   H   H   P   A   T   W   G   P   A   Q   G   V   H   V  F2
       L   L   P   P   A   P   R   G   C   S   Q   R   P   S   L   A   S   P   A   T   T   R   P   R   G   A   R   H   R   A   S   T   C  F3
6301 CCCTCCTCCCCCCAGCCCCGCGGGGGTGCTCCCAGCGCCCGAGCCTGGCATCTCCAGCCACCACCCGGCCACGTGGGGCCCGGCACAGGGCGTCCACGTG 6400
     ----:----|----:----|----:----|----:----|----:----|----:----|----:----|----:----|----:----|----:----|

       W   S   D   C   F   N   A   G   P   G   P   G   P   G   P   G   L   A   S   G   P   R   R   P   E   L   L   P   R   S   S   S   G   F1
     Y   G   P   T   A   S   T   P   A   P   A   P   A   P   A   P   G   W   P   Q   D   P   D   A   P   N   Y   C   P   D   P   L   P   V F2
      M   V   R   L   L   Q   R   R   P   R   P   R   P   R   P   R   A   G   L   R   T   P   T   P   R   T   T   A   Q   I   L   F   R   F3
6401 TATGGTCCGACTGCTTCAACGCCGGCCCCGGCCCCGGCCCCGGCCCCGGGCTGGCCTCAGGACCCCGACGCCCCGAACTACTGCCCAGATCCTCTTCCGG 6500
     ----:----|----:----|----:----|----:----|----:----|----:----|----:----|----:----|----:----|----:----|
```

```
      S  A  S  *  L  C  P  D  V  L  I  P  G  A  L  F  Q  T  L  L  S  L  E  I  C  V  P  C  G  Q  G  *  F     F1
       L  L  P  D  C  A  R  M  F  S  F  Q  E  L  F  S  K  P  S  S  P  L  K  S  V  S  P  V  D  K  D  D  L    F2
     F  C  F  L  T  V  P  G  C  S  H  S  R  S  S  F  P  N  P  P  L  P  *  N  L  C  P  L  W  T  R  M  I  *   F3
6501 TTCTGCTTCCTGACTGTGCCCGGATGTTCTCATTCCAGGAGCTCTTTTCCAAACCCTCCTCTCCCTTGAAATCTGTGTCCCCTGTGGACAAGGATGATTT 6600
     ----:----|----:----|----:----|----:----|----:----|----:----|----:----|----:----|----:----|----:----|

     R  L  P  A  V  H  K  F  T  G  S  W  S  S  V  P  A  K  S  C  R  P  R  G  A  R  E  D  Q  S  A  V  S  L   F1
     G  S  Q  R  F  T  N  L  Q  G  R  G  H  P  S  P  Q  S  P  A  D  P  E  G  P  E  R  T  R  A  L  *  A     F2
      A  P  S  G  S  Q  I  Y  R  V  V  V  I  R  P  R  K  V  L  Q  T  P  R  G  Q  R  G  P  E  R  C  E  P    F3
6601 AGGCTCCCAGCGGTTCACAAATTTACAGGGTCGTGGTCATCCGTCCCCGCAAAGTCCTGCAGACCCCGAGGGGCCAGAGAGGACCAGAGCGCTGTGAGCC 6700
     ----:----|----:----|----:----|----:----|----:----|----:----|----:----|----:----|----:----|----:----|

      H  V  A  P  P  R  P  G  R  C  D  G  L  V  F  T  A  P  Y  C  V  H  L  P  *  S  S  L  G  R  G  R  F    F1
     C  T  W  R  P  R  V  R  E  G  V  M  A  W  S  S  Q  L  L  T  V  C  T  S  P  D  P  A  W  G  G  G  V  S   F2
      A  R  G  A  P  A  S  G  K  V  *  W  L  G  L  H  S  S  L  L  C  A  P  P  L  I  Q  P  G  E  G  A  F    F3
6701 TGCACGTGGCGCCCCCGCGTCCGGGAAGGTGTGATGGCTTGGTCTTCACAGCTCCTTACTGTGTGCACCTCCCCTGATCCAGCCTGGGGAGGGGGCGTTT 6800
     ----:----|----:----|----:----|----:----|----:----|----:----|----:----|----:----|----:----|----:----|

      C  V  T  V  R  P  P  R  V  A  K  P  *  R  S  L  P  G  N  L  G  C  T  W  E  L  L  S  V  E  S  L  L    F1
       V  S  L  *  G  H  R  G  S  Q  N  P  E  D  P  C  P  V  I  W  G  A  H  G  N  C  C  Q  W  N  R  Y  W   F2
     L  C  H  C  E  A  T  A  G  R  K  T  L  K  I  P  A  R  *  F  G  V  H  M  G  T  A  V  S  G  I  A  T  G   F3
6801 CTGTGTCACTGTGAGGCCACCGCGGGTCGCAAAACCCTGAAGATCCCTGCCCGGTAATTTGGGGTGCACATGGGAACTGCTGTCAGTGGAATCGCTACTG 6900
     ----:----|----:----|----:----|----:----|----:----|----:----|----:----|----:----|----:----|----:----|

     A  V  R  V  C  S  G  S  V  D  A  A  G  E  L  S  V  Q  A  K  P  S  F  A  Q  P  P  E  C  *  G  F  E  I   F1
      Q  F  V  C  V  P  A  P  W  M  L  Q  E  N  F  L  C  K  R  N  H  P  S  P  S  P  L  N  V  E  D  L  R    F2
      S  S  C  V  F  R  L  R  G  C  C  R  R  T  F  C  A  S  E  T  I  L  R  P  A  P  *  M  L  R  I  *  D    F3
6901 GCAGTTCGTGTGTGTTCCGGCTCCGTGGATGCTGCAGGAGAACTTTCTGTGCAAGCGAAACCATCCTTCGCCCAGCCCCCTGAATGTTGAGGATTTGAGA 7000
     ----:----|----:----|----:----|----:----|----:----|----:----|----:----|----:----|----:----|----:----|

      C  L  S  L  L  V  V  K  V  L  Q  T  F  F  P  C  V  F  S  I  S  R  I  F  L  I  L  M  L  C  I  S  V    F1
     Y  V  Y  L  F  W  W  L  K  C  C  K  H  F  S  H  V  Y  F  L  *  V  E  F  S  *  F  *  C  C  V  F  Q  C   F2
      M  S  I  S  F  G  G  *  S  V  A  N  I  F  P  M  C  I  F  Y  K  *  N  F  L  D  F  N  V  V  Y  F  S    F3
7001 TATGTCTATCTCTTTTGGTGGTTAAAGTGTTGCAAACATTTTTCCCATGTGTATTTTCTATAAGTAGAATTTTCTTGATTTTAATGTTGTGTATTTCAGT 7100
     ----:----|----:----|----:----|----:----|----:----|----:----|----:----|----:----|----:----|----:----|

      P  L  V  I  F  C  D  E  F  V  F  F  F  R  L  H  Q  W  H  T  D  D  T  L  K  K  N  R  I  K  K  F  *    F1
       L  L  L  S  F  V  M  N  S  C  F  S  F  V  Y  I  S  G  T  L  M  I  R  *  K  K  I  E  *  K  N  F  K   F2
     A  S  C  Y  L  L  *  *  I  R  V  F  L  S  F  T  S  V  A  H  *  *  Y  A  E  K  K  *  N  K  K  I  L  N   F3
7101 GCCTCTTGTTATCTTTTGTGATGAATTCGTGTTTTTCTTTCGTTTACATCAGTGGCACACTGATGATACGCTGAAAAAAAAATAGAATAAAAAAAATTTTAA 7200
     ----:----|----:----|----:----|----:----|----:----|----:----|----:----|----:----|----:----|----:----|
```

Cloning primer ←

```
      I  N  L  K  *  F  F  I  R  *  A  P  I  F  K  L  T  *  S  L  *  K  L  T  V  Q  G  F  S  G  G  P  V  V   F1
       *  I  *  N  D  F  L  *  D  R  P  P  F  S  S  *  H  K  V  Y  K  S  *  L  F  R  V  S  L  V  V  Q  W    F2
        K  F  K  M  I  F  Y  K  I  G  P  H  F  Q  V  D  I  K  F  I  K  A  D  C  S  G  F  L  W  W  S  S  G   F3
7201 ATAAATTTAAAATGATTTTTTATAAGATAGGCCCCCATTTTCAAGTTGACATAAAGTTTATAAAAGCTGACTGTTCAGGGTTTCTCTGGTGGTCCAGTGG 7300
     ----:----|----:----|----:----|----:----|----:----|----:----|----:----|----:----|----:----|----:----|

        K  N  P  P  C  N  A  R  D  T  S  S  I  P  G  P  G  R  C  H  T  P  Q  L  R  R  P  A  H  P  R  V  W   F1
      L  R  I  H  L  A  M  P  G  T  P  V  Q  Y  L  V  Q  E  D  A  T  R  H  S  Y  E  D  L  H  I  L  E  F  G  F2
       *  E  S  T  L  Q  C  Q  G  H  Q  F  N  T  W  S  R  K  M  P  H  A  T  A  T  K  T  C  T  S  *  S  L   F3
7301 TTAAGAATCCACCTTGCAATGCCAGGGACACCAGTTCAATACCTGGTCCAGGAAGATGCCACACGCCACAGCTACGAAGACCTGCACATCCTAGAGTTTG 7400
     ----:----|----:----|----:----|----:----|----:----|----:----|----:----|----:----|----:----|----:----|

       P  L  *  Q  E  K  P  P  *  *  T  R  E  S  L  C  S  S  E  D  P  A  Q  P  K  I  I  F  L  K  N  S  T    F1
        L  C  N  R  R  S  H  L  N  K  L  E  K  V  C  A  A  V  K  T  Q  H  S  P  K  *  F  F  *  K  T  R  H   F2
      A  F  V  T  G  E  A  T  L  I  N  *  R  K  F  V  Q  Q  *  R  P  S  T  A  Q  N  N  F  F  K  K  L  D  I  F3
7401 GCCTTTGTAACAGGAGAAGCCACCTTAATAAACTAGAGAAAGTTTGTGCAGCAGTGAAGACCCAGCACAGCCCAAAATAATTTTTTTAAAAAACTCGACA 7500
     ----:----|----:----|----:----|----:----|----:----|----:----|----:----|----:----|----:----|----:----|

      F  K  V  C  L  L  C  H  K  L  K  T  V  Y  R  *  Q  *  S  E  I  P  S  L  L  F  P  I  *  L  R  G  F  T  F1
       S  R  S  A  C  F  V  T  S  *  K  Q  C  T  D  N  S  S  R  R  F  P  V  F  C  F  Q  Y  S  S  G  V  S    F2
        Q  G  L  L  A  L  S  Q  A  E  N  S  V  Q  I  T  V  V  G  D  S  Q  S  S  V  S  N  I  A  Q  G  F  H   F3
7501 TTCAAGGTCTGCTTGCTTTGTCACAAGCTGAAAACAGTGTACAGATAACAGTAGTCGGAGATTCCCAGTCTTCTGTTTCCAATATAGCTCAGGGGTTTCA 7600
     ----:----|----:----|----:----|----:----|----:----|----:----|----:----|----:----|----:----|----:----|

        W  Q  S  I  P  V  F  L  S  W  R  I  P  G  T  E  E  S  D  R  L  Q  S  M  G  L  Q  T  V  G  H  *  A   F1
      H  G  N  P  F  Q  Y  S  C  L  G  E  S  Q  G  Q  R  S  L  T  G  Y  S  P  W  G  C  K  Q  W  D  T  E  R  F2
       M  A  I  H  S  S  I  L  V  L  E  N  P  R  D  R  G  V  *  Q  A  T  V  H  G  V  A  N  S  G  T  L  S    F3
7601 CATGGCAATCCATTCCAGTATTCTTGTCTTGGAGAATCCCAGGGACAGAGGAGTCTGACAGGCTACAGTCCATGGGGTTGCAAACAGTGGGACACTGAGC 7700
     ----:----|----:----|----:----|----:----|----:----|----:----|----:----|----:----|----:----|----:----|

       T  N  T  G  V  S  G  A  G  V  W  R  E  E  L  L  F  S  S  P  A  L  L  L  F  K  K  Y  L  F  G  C  I    F1
        L  T  R  G  F  L  G  Q  G  C  G  G  R  S  C  S  F  L  L  L  H  Y  C  F  L  K  S  I  Y  L  A  A  S   F2
      D  *  H  G  G  F  W  G  R  G  V  E  G  G  V  A  L  F  F  S  C  I  T  A  F  *  K  V  F  I  W  L  H  L  F3
7701 GACTAACACGGGGGTTTCTGGGGCAGGGGTGTGGAGGGAGGAGTTGCTCTTTTCTTCTCCTGCATTACTGCTTTTTAAAAAGTATTTATTTGGCTGCATC 7800
     ----:----|----:----|----:----|----:----|----:----|----:----|----:----|----:----|----:----|----:----|

      *  S  Q  L  G  H  V  G  S  L  L  F  A  C  S  H  C  S  M  R  A  Q  Y  L  W  H  A  D  L  I  A  L  Q  H  F1
       D  H  S  W  G  M  W  D  R  C  C  L  L  A  A  I  V  A  *  G  L  S  I  C  G  T  Q  T  *  L  L  C  S    F2
        I  T  V  G  A  C  G  I  V  A  V  C  L  Q  P  L  *  H  E  G  S  V  F  V  A  R  R  L  N  C  F  A  A   F3
7801 TGATCACAGTTGGGGCATGTGGGATCGTTGCTGTTTGCTTGCAGCCATTGTAGCATGAGGGCTCAGTATTTGTGGCACGCAGACTTAATTGCTTTGCAGC 7900
     ----:----|----:----|----:----|----:----|----:----|----:----|----:----|----:----|----:----|----:----|

        V  G  F  *  F  P  S  Q  E  W  N  P  S  P  L  H  V  K  E  D  S  *  P  L  D  H  Q  G  S  P  Y  C  S   F1
      M  W  G  F  N  F  P  A  K  N  G  T  Q  V  P  C  M  S  R  R  I  L  S  H  W  T  T  R  E  V  P  T  A  L  F2
```

```
       C  G  V  L  I  S  Q  P  R  M  E  P  K  S  P  A  C  Q  G  G  F  L  A  T  G  P  P  G  K  S  L  L  L    F3
7901 ATGTGGGGTTTTAATTTCCCAGCCAAGAATGGAACCCAAGTCCCCTGCATGTCAAGGAGGATTCTTAGCCACTGGACCACCAGGGAAGTCCCTACTGCTC 8000
     ----:----|----:----|----:----|----:----|----:----|----:----|----:----|----:----|----:----|----:----|

       S  L  S  S  C  L  R  Y  L  S  L  S  H  K  D  N  V  F  F  P  K  I  I  T  V  L  T  P  R  S  *  N  N    F1
        L  F  P  L  V  *  D  I  F  H  Y  H  I  K  I  M  S  S  S  L  K  S  *  Q  F  *  L  L  G  L  K  T  T   F2
      F  S  F  L  L  F  K  I  S  F  I  I  T  *  R  *  C  L  L  P  *  N  H  N  S  F  N  S  *  V  L  K  Q  L   F3
8001 TTCTCTTTCCTCTTGTTTAAGATATCTTTCATTATCACATAAAGATAATGTCTTCTTCCCTAAAATCATAACAGTTTTAACTCCTAGGTCTTAAAACAAC 8100
     ----:----|----:----|----:----|----:----|----:----|----:----|----:----|----:----|----:----|----:----|

      *  N  *  L  W  M  Y  G  V  R  *  G  F  L  F  V  F  F  S  L  M  D  F  H  P  R  G  L  S  G  K  E  A  V   F1
       R  T  D  C  G  C  M  V  *  G  K  G  F  F  L  C  F  F  P  S  W  I  S  I  P  G  A  S  V  V  K  K  L    F2
        E  L  I  V  D  V  W  C  E  V  R  V  S  F  C  V  F  F  P  H  G  F  P  S  Q  G  P  Q  W  *  R  S  C   F3
8101 TAGAACTGATTGTGGATGTATGGTGTGAGGTAAGGGTTTCTTTTTGTGTTTTTTTCCCTCATGGATTTCCATCCCAGGGGCCTCAGTGGTAAAGAAGCTG 8200
     ----:----|----:----|----:----|----:----|----:----|----:----|----:----|----:----|----:----|----:----|

        C  Q  C  R  R  R  R  F  N  P  W  G  R  K  I  P  R  R  R  K  W  Q  A  T  P  V  F  L  P  G  K  S  H   F1
      S  A  N  A  G  D  A  G  S  I  P  G  A  E  R  S  P  G  E  G  S  G  K  P  L  Q  Y  S  C  L  G  N  P  M   F2
       L  P  M  Q  E  T  Q  V  Q  S  L  G  Q  K  D  P  Q  E  K  E  V  A  S  H  S  S  I  L  A  W  E  I  P    F3
8201 TCTGCCAATGCAGGAGACGCAGGTTCAATCCCTGGGGCAGAAAGATCCCCAGGAGAAGGAAGTGGCAAGCCACTCCAGTATTCTTGCCTGGGAAATCCCA 8300
     ----:----|----:----|----:----|----:----|----:----|----:----|----:----|----:----|----:----|----:----|
```
