# Supplementary material for: Molecular cloning of PRD-like homeobox genes expressed in bovine oocytes and early IVF embryos
Source: BMC Genomics. 2024 Nov 6;25:1048. doi: 10.1186/s12864-024-10969-w (PMC11542365; doi:10.1186/s12864-024-10969-w)
Supplement: Supplementary file 9 — Supplementary Material 9: Additional file 15: Figure S7. The prediction of TPRX2 derived from Bos taurus isolate L1 Dominette 01449 registration number 42190680 breed Hereford chromosome 18, ARS-UCD1.2, whole genome shotgun sequence. Three possible ORFs for exons, but not introns, are depicted. Putative protein sequence is highlighted in yellow. Sequences from StringTie merge prediction and confirmed cDNA are drawn as lines below the corresponding sequences. Cloning primers are drawn as line arrows. Splice sites are underlined and codons split by two exons are coloured red. The homeodomain is highlighted in green. [file 12864_2024_10969_MOESM9_ESM.pdf]

Supplementary Figure S7. The prediction of *TPRX2* derived from *Bos taurus* isolate L1 Dominette 01449 registration number 42190680 breed Hereford chromosome 18, ARS-UCD1.2, whole genome shotgun sequence. Three possible ORFs for exons, but not introns, are depicted. Putative protein sequence is highlighted in yellow. Sequences from StringTie merge prediction and confirmed cDNA are drawn as lines below the corresponding sequences. Cloning primers are drawn as line arrows. Splice sites are underlined and codons split by two exons are coloured red. The homeodomain is highlighted in green.

```
                                                                       A  S  N  S  D  S  R  T  A  G  G  C         F1
                                                                        H  Q  T  P  T  A  E  Q  Q  E  D  A        F2
                                                                      S  I  K  L  R  Q  Q  N  S  R  R  M          F3
 9401                                                                 AGCATCAAACTCCGACAGCAGAACAGCAGGAGGATG 9500
                                                                      :----|----:----|----:----|----:----|
                                                         Cloning primer
                                                         TPRX2_16c_01

       K  N  P  S  P  S  *                                                                                        F1
        R  T  Q  V  R  H  E                                                                                       F2
      Q  E  P  K  S  V  M  S                                                                                      F3
 9501 CAAGAACCCAAGTCCGTCATGAGTGAGTCTGGCCTCCTGGGACTTAGTGGAGTTTGGGGGTGGAATCACATTACTAGGGGAGAGGAGCAGAGAGCCTGGT 9600
      ----:----|----:----|----:----|----:----|----:----|----:----|----:----|----:----|----:----|----:----|

 9601 CCTCGGGGGTCCCCTGTCAGGGTGGCTCCCCACTTGGTAGGGGGTGATGATAGGGGGTTGGGGAGACCACCAAGGGTCACCAAGGAGTTTGTCCTGCCTG 9700
      ----:----|----:----|----:----|----:----|----:----|----:----|----:----|----:----|----:----|----:----|

 9701 CCTTCCCAGGGCAGGGCCCTGCATAAATACTCCACCGGCTGAGATCAAGCCCATTTCCAAGCTGTGCTCACTGAGGCCTGGAGAGGTGTAGGGGTTAGGG 9800
      ----:----|----:----|----:----|----:----|----:----|----:----|----:----|----:----|----:----|----:----|

 9801 CTGGAGGGCAGACAGCCAGGGTCTAACCAACAACCTCACTCCGGATGCCGCTGGCTTCTCCTCCCGCCCTCGGGCCTGAGTCCCACCGGCTCACCAGGGT 9900
      ----:----|----:----|----:----|----:----|----:----|----:----|----:----|----:----|----:----|----:----|

                                                                                       P  H  G  E  I  S  A        F1
                                                                                        L  T  G  R  S  A          F2
                                                                                      A  S  R  G  D  Q  R         F3
 9901 CTGGTCCAGGGAGGGGGAGGGCGCCAGGCAGGCTGGGGGCTATTCCTGGGCTCATGGGACCCTGTTTCCCCTTGGTCAGGCCTCACGGGGAGATCAGCG 10000
      ----:----|----:----|----:----|----:----|----:----|----:----|----:----|----:----|----:----|----:----|

        P  K  Q  A  A  A  G  A  H  G  V  Q  Q  R  T  V  G  G  A  Q  R  S  L  P  E  E  R  V  P  E  L  P  G       F1
      P  P  N  R  Q  R  Q  E  R  T  V  Y  S  K  E  Q  L  E  A  L  K  E  A  F  L  K  N  E  Y  P  S  Y  Q  D      F2
       P  Q  T  G  S  G  R  S  A  R  C  T  A  K  N  S  W  R  R  S  K  K  P  S  *  R  T  S  T  R  V  T  R        F3
10001 CCCCCAAACAGGCAGCGGCAGGAGCGCACGGTGTACAGCAAAGAACAGTTGGAGGCGCTCAAAGAAGCCTTCCTGAAGAACGAGTACCCGAGTTACCAGG 10100
      ----:----|----:----|----:----|----:----|----:----|----:----|----:----|----:----|----:----|----:----|

       P  P  A  P  G  G  Q  A  P  P  G  R  T  Q  S  A                                                             F1
        R  L  R  L  A  A  R  L  H  L  D  E  H  R  V  Q                                                            F2
      T  A  C  A  W  R  P  G  S  T  W  T  N  T  E  C                                                              F3
```

```
10101 ACCGCCTGCGCCTGGCGGCCAGGCTCCACCTGGACGAACACAGAGTGCAGGTCTGAGCCCGCCGCCCCGGGGCCACCCCTCCGCCCTCCCAGAGCCACCG 10200
      ----:----|----:----|----:----|----:----|----:----|----:----|----:----|----:----|----:----|----:----|

10201 CTGGGGACCGCTAGGCCAGAGGGGCCAGGGCTGCCCCGGGTGCCGCAGCAAGCAAGGGGTCTCCCTGCTGTGGACCCAGGGCTGGACCCCTTCCGCCTCA 10300
      ----:----|----:----|----:----|----:----|----:----|----:----|----:----|----:----|----:----|----:----|

10301 ACCCTGTGCTGTATCACCCCGCCAACCGCATGCGGCCTGCCTGACACCCCCCATCGCCCCGGGGTGTCCCGGTGCCCAGCCCTCAGGGCCTCTGACCCCG 10400
      ----:----|----:----|----:----|----:----|----:----|----:----|----:----|----:----|----:----|----:----|

10401 CGGACCCTCTCGGGCAGACCCCTTCCTGCCCCGTGGGCGCCCAAGCCCTGCCCCCTGCCCTCTGCACCCCTTCCCGCTGCCTCCTGAACCCCAGGAGCCT 10500
      ----:----|----:----|----:----|----:----|----:----|----:----|----:----|----:----|----:----|----:----|

10501 CTAATTGGGTCTGGGGGGCGGAGGGAGAGGGGAGGCGGGATTCTCAGCTGGACAGCCTCACCCCCGGAGCAGAGGCGATAGCGCGGGGCCCCTCGCCTCT 10600
      ----:----|----:----|----:----|----:----|----:----|----:----|----:----|----:----|----:----|----:----|

                                         V  V  Q  E  P  P  G  P  A  L  P  S  G  A  T  T  G  P  G  R  M    F1
                                        V  W  F  K  N  R  R  A  Q  R  S  R  L  E  R  R  Q  A  Q  G  G  C  F2
                                         C  G  S  R  T  A  G  P  S  A  P  V  W  S  D  D  R  P  R  E  D    F3
10601 CACACCCGCTTCTCCCGGTCCCCTCTGCCCACCCAGGTGTGGTTCAAGAACCGCCGGGCCCAGCGCTCCCGTCTGGAGCGACGACAGGCCCAGGGAGGAT 10700
      ----:----|----:----|----:----|----:----|----:----|----:----|----:----|----:----|----:----|----:----|

        L  E  G  R  R  C  A  H  G  P  W  S  P  L  D  P  C  P  R  P  G  I  C  C  S  S  W  E  P  Q  L  L  R    F1
        W  R  A  G  D  A  P  T  D  P  G  V  P  W  T  P  A  P  A  P  E  S  A  A  A  A  G  S  P  S  Y  S  G    F2
      A  G  G  P  A  M  R  P  R  T  L  E  S  P  G  P  L  P  P  P  R  N  L  L  Q  Q  L  G  A  P  V  T  Q  A  F3
10701 GCTGGAGGGCCGGCGATGCGCCCACGGACCCTGGAGTCCCCTGGACCCCTGCCCCCGCCCCGGAATCTGCTGCAGCAGCTGGGAGCCCCAGTTACTCAGG 10800
      ----:----|----:----|----:----|----:----|----:----|----:----|----:----|----:----|----:----|----:----|
                StringTie merge

      L  P  G  I  L  Q  P  P  S  S  P  Q  P  R  G  C  A  P  S  S  R  A  R  R  L  Q  S  S  P  G  H  V  G  P  F1
       C  P  G  F  Y  S  R  P  P  P  P  S  P  A  G  V  L  P  A  P  E  P  G  V  S  S  H  H  P  A  T  W  G    F2
        A  R  D  S  T  A  A  L  L  P  P  A  P  R  V  C  S  Q  L  P  S  P  A  S  P  V  I  T  R  P  R  G  A  F3
10801 CTGCCCGGGATTCTACAGCCGCCCTCCTCCCCCCAGCCCCGCGGGTGTGCTCCCAGCTCCCGAGCCCGGCGTCTCCAGTCATCACCCGGCCACGTGGGGC 10900
      ----:----|----:----|----:----|----:----|----:----|----:----|----:----|----:----|----:----|----:----|

        G  T  G  R  P  R  V  W  P  G  C  S  N  P  G  P  G  P  G  L  A  S  G  P  L  R  A  N  R  P  P  R  S   F1
      P  A  Q  G  V  H  V  Y  G  P  A  A  P  T  P  A  P  A  P  G  W  P  Q  D  P  Y  V  P  I  D  R  P  D  P  F2
       R  H  R  A  S  T  C  M  A  R  L  L  Q  P  R  P  R  P  R  A  G  L  R  T  P  T  C  Q  S  T  A  Q  I   F3
10901 CCGGCACAGGGCGTCCACGTGTATGGCCCGGCTGCTCCAACCCCGGCCCCGGCCCCGGGCTGGCCTCAGGACCCCTACGTGCCAATCGACCGCCCAGATC 11000
      ----:----|----:----|----:----|----:----|----:----|----:----|----:----|----:----|----:----|----:----|

       S  S  A  S  *  L  C  S  D  V  F  I  P  R  A  L  L  H  D  V  W  V  P  N  E  R  *  L  *  G  *  E  *    F1
        L  P  L  P  D  C  A  L  M  F  S  S  Q  E  L  S  S  T  T  S  G  Y  Q  T  R  D  S  F  E  D  E  N  D   F2
      L  F  R  F  L  T  V  L  *  C  F  H  P  K  S  S  P  P  R  R  L  G  T  K  R  E  I  A  L  R  M  R  M  T  F3
```

```
11001 CTCTTCCGCTTCCTGACTGTGCTCTGATGTTTTCATCCCAAGAGCTCTCCTCCACGACGTCTGGGTACCAAACGAGAGATAGCTTTGAGGATGAGAATGA 11100
      ----:----|----:----|----:----|----:----|----:----|----:----|----:----|----:----|----:----|----:----|

      H  R  P  P  A  V  H  E  F  V  G  S  W  S  S  V  P  T  K  S  C  R  P  R  G  A  R  G  D  E  G  L  V  S   F1
       T  G  P  R  Q  F  T  N  L  *  G  R  G  H  L  S  P  Q  S  P  A  D  P  E  G  P  E  G  M  R  A  L  *    F2
        Q  A  P  G  S  S  R  I  C  R  V  V  V  I  C  P  H  K  V  L  Q  T  P  R  G  Q  R  G  *  G  P  C  E   F3
11101 CACAGGCCCCCGGCAGTTCACGAATTTGTAGGGTCGTGGTCATCTGTCCCCACAAAGTCCTGCAGACCCCGAGGGGCCAGAGGGGATGAGGGCCTTGTGA 11200
      ----:----|----:----|----:----|----:----|----:----|----:----|----:----|----:----|----:----|----:----|

       L  H  V  V  P  M  G  T  R  E  G  V  M  A  W  S  S  Q  L  V  Y  C  P  Q  R  D  C  T  S  L  D  P  A    F1
      V  C  T  W  C  P  W  G  R  G  K  V  *  W  L  G  L  H  S  W  F  T  V  R  K  G  T  A  P  P  S  I  Q  P   F2
       S  A  R  G  A  H  G  D  A  G  R  C  D  G  L  V  F  T  A  G  L  L  S  A  K  G  L  H  L  P  R  S  S    F3
11201 GTCTGCACGTGGTGCCCATGGGGACGCGGGAAGGTGTGATGGCTTGGTCTTCACAGCTGGTTTACTGTCCGCAAAGGGACTGCACCTCCCTCGATCCAGC 11300
      ----:----|----:----|----:----|----:----|----:----|----:----|----:----|----:----|----:----|----:----|

       W  R  G  G  V  F  A  C  V  T  M  R  P  S  Q  V  S  K  L  L  M  S  L  P  S  L  V  F  E  A  H  V  G    F1
        G  E  G  A  F  L  R  V  S  L  *  G  H  L  R  S  Q  S  S  *  C  P  C  P  R  *  Y  L  R  R  T  W  A   F2
      L  E  R  G  R  F  C  V  C  H  Y  E  A  I  S  G  L  K  A  L  N  V  P  A  L  V  S  I  *  G  A  R  G  H   F3
11301 CTGGAGAGGGGGCGTTTTTGCGTGTGTCACTATGAGGCCATCTCAGGTCTCAAAGCTCTTAATGTCCCTGCCCTCGTTAGTATTTGAGGCGCACGTGGGC 11400
      ----:----|----:----|----:----|----:----|----:----|----:----|----:----|----:----|----:----|----:----|

      I  S  V  N  F  Q  V  R  G  G  T  I  V  H  S  V  C  F  T  V  V  G  T  C  R  T  F  T  S  L  I  G  N  L   F1
       F  L  *  I  S  K  S  E  V  G  L  L  F  T  V  S  V  S  L  L  W  E  L  V  G  L  S  P  A  S  *  G  I    F2
        F  C  E  F  P  S  P  R  W  D  Y  C  S  Q  C  L  F  H  C  C  G  N  L  *  D  F  H  Q  P  H  R  E  F   F3
11401 ATTTCTGTGAATTTCCAAGTCCGAGGTGGGACTATTGTTCACAGTGTCTGTTTCACTGTTGTGGGAACTTGTAGGACTTTCACCAGCCTCATAGGGAATT 11500
      ----:----|----:----|----:----|----:----|----:----|----:----|----:----|----:----|----:----|----:----|

       I  K  I  A  L  N  L  Y  L  P  L  M  S  R  D  F  C  F  C  L  S  L  S  L  P  V  V  S  N  K  C  L  Q    F1
      *  L  K  L  P  *  I  F  T  F  P  *  C  L  E  I  F  V  F  A  C  L  Y  P  Y  L  *  F  Q  I  N  V  C  K   F2
       N  *  N  C  L  K  S  L  P  S  L  D  V  *  R  F  L  F  L  L  V  S  I  L  T  C  S  F  K  *  M  F  A    F3
11501 TAATTAAAATTGCCTTAAATCTTTACCTTCCCTTGATGTCTAGAGATTTTTGTTTTTGCTTGTCTCTATCCTTACCTGTAGTTTCAAATAAATGTTTGCA 11600
      ----:----|----:----|----:----|----:----|----:----|----:----|----:----|----:----|----:----|----:----|
                                                   Cloning primer <---------------

       N  *  K  R  E  H  *  I  H  K  *  F  W  S  *  L  A  S  L  Q  Y  *  A  F  Q  S  R  Y  I  T  Y  *  S    F1
        I  K  K  G  S  I  K  S  T  N  S  F  G  A  N  W  H  L  Y  N  T  E  P  S  N  L  G  I  L  L  I  N  H   F2
      K  L  K  K  G  A  L  N  P  Q  I  V  L  E  L  I  G  I  F  T  I  L  S  L  P  I  *  V  Y  Y  L  L  I  I   F3
11601 AAATTAAAAAAGGGAGCATTAAATCCACAAATAGTTTTGGAGCTAATTGGCATCTTTACAATACTGAGCCTTCCAATCTAGGTATATTACTTATTAATCA 11700
      ----:----|----:----|----:----|----:----|----:----|----:----|----:----|----:----|----:----|----:----|

      F  T  W  L  F  T  K  I  L  R  *  V  L  F  V  *  V  C  F  Q  C  F  V  L  *  L  P  D  I  L  D  E  L  K   F1
       S  L  G  C  L  Q  K  F  S  A  K  C  Y  L  C  R  F  V  S  S  A  L  S  C  D  Y  L  I  F  *  M  N  *    F2
        H  L  V  V  Y  K  N  S  P  L  S  V  I  C  V  G  L  F  P  V  L  C  L  V  I  T  *  Y  F  R  *  I  K   F3
```

```
11701 TTCACTTGGTTGTTTACAAAAATTCTCCGCTAAGTGTTATTTGTGTAGGTTTGTTTCCAGTGCTTTGTCTTGTGATTACCTGATATTTTAGATGAATTAA 11800
      ----:----|----:----|----:----|----:----|----:----|----:----|----:----|----:----|----:----|----:----|

        F  L  F  *  N  L  C  S  A  *  M  S  I  G  *  R  M  D  K  K  A  V  V  H  I  H  N  G  M  N  I  T  Q    F1
      N  F  Y  F  K  I  C  V  L  P  R  C  P  L  A  D  E  W  I  R  K  L  W  Y  I  Y  T  M  E  *  I  L  L  S   F2
       I  F  I  L  K  S  V  F  C  L  D  V  H  W  L  T  N  G  *  E  S  C  G  T  Y  T  Q  W  N  E  Y  Y  S     F3
11801 AATTTTTATTTTAAAATCTGTGTTCTGCCTAGATGTCCATTGGCTGACGAATGGATAAGAAAGCTGTGGTACATATACACAATGGAATGAATATTACTCA 11900
      ----:----|----:----|----:----|----:----|----:----|----:----|----:----|----:----|----:----|----:----|

       L  L  K  R  M  H  L  N  P  F  K  *  G  G                                                              F1
        Y  *  K  E  C  I  *  I  R  S  N  E  V  D                                                             F2
      A  I  K  K  N  A  F  E  S  V  Q  M  R  W  M                                                            F3
11901 GCTATTAAAAAGAATGCATTTGAATCCGTTCAAATGAGGTGGATG                                                       12000
      ----:----|----:----|----:----|----:----|----:
```
