# Supplementary material for: Molecular cloning of PRD-like homeobox genes expressed in bovine oocytes and early IVF embryos
Source: BMC Genomics. 2024 Nov 6;25:1048. doi: 10.1186/s12864-024-10969-w (PMC11542365; doi:10.1186/s12864-024-10969-w)
Supplement: Supplementary file 10 — Supplementary Material 10: S10 Additional file 16: Figure S8. The prediction of TPRX3 derived from Bos taurus isolate L1 Dominette 01449 registration number 42190680 breed Hereford chromosome 18, ARS-UCD1.2, whole genome shotgun sequence. Three possible ORFs for exons, but not introns, are depicted. Putative protein sequence and homeodomain are highlighted in yellow and green, respectively. Sequences from StringTie merge prediction and confirmed cDNA are drawn as lines below the corresponding sequences. Cloning primers are drawn as line arrows. Splice sites are underlined and codons split by two exons are coloured red. The homeodomain is highlighted in green. [file 12864_2024_10969_MOESM10_ESM.pdf]

Supplementary Figure S8. The prediction of *TPRX3* derived from *Bos taurus* isolate L1 Dominette 01449 registration number 42190680 breed Hereford chromosome 18, ARS-UCD1.2, whole genome shotgun sequence. Three possible ORFs for exons, but not introns, are depicted. Putative protein sequence and homeodomain are highlighted in yellow and green, respectively. Sequences from StringTie merge prediction and confirmed cDNA are drawn as lines below the corresponding sequences. Cloning primers are drawn as line arrows. Splice sites are underlined and codons split by two exons are coloured red. The homeodomain is highlighted in green.

```
        P  Q  T  F  L  Q  S  P  K  G  G  E  Q  S  S  S  E  E  P  R  T  S  V  Y  H  G          F1
         L  R  P  S  C  R  A  P  K  V  E  S  R  A  A  V  R  N  Q  E  P  Q  S  T  M  G         F2
       A  S  D  L  P  A  E  P  Q  R  W  R  A  E  Q  Q  *  G  T  K  N  L  S  L  P  W           F3
5001 GCCTCAGACCTTCCTGCAGAGCCCCAAAGGTGGAGAGCAGAGCAGCAGTGAGGAACCAAGAACCTCAGTCTACCATGGGTGAGTACGGGCCAGGGCGAAA 5100
     ----:----|----:----|----:----|----:----|----:----|----:----|----:----|----:----|----:----|----:----|
                              Cloning primer --------------------->
StringTie merge ------------------------------------------------------------------------------------
                              TPRX3_8c_01 ----------------------------------------------------------

5101 GGCCTGGGCTAGAGGCTGGGGGTCATGGCCGAGGCCGGAGGTCTCCTGGGTACCCAGAGGAAGGCCCAGCTGTCCGAGAGGGCTTCCTGTGGTGGGGAGG 5200
     ----:----|----:----|----:----|----:----|----:----|----:----|----:----|----:----|----:----|----:----|

5201 ACGCTGCAGAGTCTGGGGCATGGAATTACGTTATCTGGACAAGATGAACAGAGAGCCAGCCCACCGCGGCCCCCTGCAAACATCAGGGTCACTTCAGCCC 5300
     ----:----|----:----|----:----|----:----|----:----|----:----|----:----|----:----|----:----|----:----|

5301 TTGGATAGTGAGGGGACCTGGCGCCCAGGAGAGCCCTGGGGCTGAGATCAGGCCCATCACAAGGATGTGGACACTGAGGCCCTGCTTTCGCAGGCTGGAG 5400
     ----:----|----:----|----:----|----:----|----:----|----:----|----:----|----:----|----:----|----:----|

5401 GCCCTGGGGCTGAGATCAGGTCCATCACAAGGATGTGGACACTGAGGCCCAGAGATGTGTGAGGGCTGGGGTGGGAGGAGAGCCAGGCTGGCACCAGGGG 5500
     ----:----|----:----|----:----|----:----|----:----|----:----|----:----|----:----|----:----|----:----|

5501 CGGCCGGGCCGGATGCCACACGCTTAACCTTGCACCAGCCTCTGCCGGCAAGCGTGAGCCCCTCGTCCACTCACCAGGGCCTGAGCCAGGCCGGGGGAGG 5600
     ----:----|----:----|----:----|----:----|----:----|----:----|----:----|----:----|----:----|----:----|

                                                                       T  C  R  E  A  A  A  A  A  H  G  V     F1
                                                                        P  A  G  R  Q  R  R  Q  R  T  V  F    F2
                                                                      D  L  Q  G  G  S  G  G  S  A  R  C  S   F3
5601 GGGGCTGGGGACCATCTGGAGGTGGGCTTCGTCTGACCCTCCTTCTCCCCCAATGGCTCTCAGGACCTGCAGGGAGGCAGCGGCGGCAGCGCACGGTGTT 5700
     ----:----|----:----|----:----|----:----|----:----|----:----|----:----|----:----|----:----|----:----|

     Q  Q  R  T  V  T  R  A  *  G  T  L  C  K  Q  P  V  P  E  L  L  G  S  *  G  L  G  G  Q  A  Q  P  G  G  F1
      N  K  E  Q  L  R  E  L  E  E  H  F  V  N  N  Q  Y  P  S  Y  W  A  R  E  D  L  A  A  R  L  N  L  E   F2
       T  K  N  S  Y  A  S  L  R  N  T  L  *  T  T  S  T  R  A  T  G  L  V  R  T  W  R  P  G  S  T  W  R  F3
5701 CAACAAAGAACAGTTACGCGAGCTTGAGGAACACTTTGTAAACAACCAGTACCCGAGCTACTGGGCTCGTGAGGACTTGGCGGCCAGGCTCAACCTGGAG 5800
     ----:----|----:----|----:----|----:----|----:----|----:----|----:----|----:----|----:----|----:----|
```

```
      V  Q  S  A                                                                                           F1
     E  Y  K  V  Q                                                                                         F2
      S  T  K  C                                                                                           F3
5801 GAGTACAAAGTGCAGGTCAGATCCCACCCCCACCCACCGTGCCCACAGCTATACCAGGGCCAGCTCCCACCTTGGCACTTACCACGTGCCGCATCCGGGG 5900
     ----:----|----:----|----:----|----:----|----:----|----:----|----:----|----:----|----:----|----:----|

5901 GCCAGCTTTCCGCCCCTCCAAGGTCCAAGGGGCCCGAGCCGGGCCCTGGGTCACTCAGGAAGCAAGGGCTCTCCTGGGGTCCGGATCTCAGGGCCTCTGG 6000
     ----:----|----:----|----:----|----:----|----:----|----:----|----:----|----:----|----:----|----:----|

6001 GAACCCAGGGAGCCCCTCTGGCAGACAGTCTTCCTGGCGCTCGAGCTCCTGAGTGACCAAGGCTGGCCACTCCTGCCCTCTTCAGGTCTCCTCTGCGGCC 6100
     ----:----|----:----|----:----|----:----|----:----|----:----|----:----|----:----|----:----|----:----|

6101 TCTTGAAAACTCCAGGAGCCTCTAATTGGGCCTGGGGGGACCGAGGCTGGCTTCTGCTCCAACGGCGGGCAGCCTCACACCCACCCCCGGAGGCCGCAAA 6200
     ----:----|----:----|----:----|----:----|----:----|----:----|----:----|----:----|----:----|----:----|
                                                                        V  V  Q  E  P  P  G  Q  K  R  S  A  E  A  I    F1
                                                                       V  W  F  K  N  R  R  A  K  N  A  R  L  K  R  L  F2
                                                                        C  G  S  R  T  A  G  P  K  T  L  G  *  S  D  * F3
6201 TGCGCACGGCCCCTCCTTTGACGCCTCCTCCCCGGCCCCTCTGTCTTCCCCAGGTGTGGTTCAAGAACCGCCGGGCCAAAAACGCTCGGCTGAAGCGATT 6300
     ----:----|----:----|----:----|----:----|----:----|----:----|----:----|----:----|----:----|----:----|

     D  P  G  A  W  P  G  F  P  Q  R  F  H  R  R  R  S  S  R  R  P  R  S  Q  D  C  L  Y  P  C  R  R  G  C   F1
      T  Q  G  P  G  Q  G  S  R  S  A  S  T  D  G  G  V  P  D  D  P  A  P  R  T  A  S  I  P  A  A  A  A    F2
       P  R  G  L  A  R  V  P  A  A  L  P  P  T  A  E  F  P  T  T  P  L  P  G  L  P  L  S  L  P  P  R  L  F3
6301 GACCCAGGGGCCTGGCCAGGGTTCCCGCAGCGCTTCCACCGACGGCGGAGTTCCCGACGACCCCGCTCCCAGGACTGCCTCTATCCCTGCCGCCGCGGCT 6400
     ----:----|----:----|----:----|----:----|----:----|----:----|----:----|----:----|----:----|----:----|

      S  L  C  A  P  R  R  P  G  V  P  Q  P  L  S  A  Q  P  R  Q  H  A  Q  R  T  S  T  R  R  R  P  *  P  F1
     A  V  S  A  P  P  E  G  P  G  F  R  S  P  S  P  P  S  P  A  S  M  L  S  A  P  A  P  G  G  V  P  S  Q  F2
      Q  S  L  R  P  Q  K  A  R  G  S  A  A  P  L  R  P  A  P  P  A  C  S  A  H  Q  H  P  A  A  S  L  A   F3
6401 GCAGTCTCTGCGCCCCCAGAAGGCCCGGGGTTCCGCAGCCCCTCTCCGCCCAGCCCCGCCAGCATGCTCAGCGCACCAGCACCCGGCGGCGTCCCTAGCC 6500
     ----:----|----:----|----:----|----:----|----:----|----:----|----:----|----:----|----:----|----:----|

      R  P  G  Q  L  R  P  G  A  G  R  P  G  R  L  P  Y  G  S  S  S  L  D  P  R  S  N  F  S  P  D  S  R   F1
       G  Q  A  S  C  A  L  A  Q  G  A  Q  D  G  F  P  M  A  A  P  A  S  T  P  G  L  T  S  A  P  T  P  D  F2
     K  A  R  P  A  A  P  W  R  R  A  P  R  T  A  S  L  W  Q  L  Q  P  R  P  Q  V  *  L  Q  P  R  L  Q  T  F3
6501 AAGGCCAGGCCAGCTGCGCCCTGGCGCAGGGCGCCCAGGACGGCTTCCCTATGGCAGCTCCAGCCTCGACCCCAGGTCTAACTTCAGCCCCGACTCCAGA 6600
     ----:----|----:----|----:----|----:----|----:----|----:----|----:----|----:----|----:----|----:----|

     L  G  S  G  P  L  R  R  F  G  L  L  A  R  L  C  C  H  L  *  F  H  R  P  L  P  A  P  R  T  I  P  R  T  F1
      W  V  Q  D  P  C  A  A  S  D  S  L  P  D  S  V  V  I  F  D  F  T  D  L  F  P  L  Q  E  P  S  H  E   F2
       G  F  R  T  P  A  P  L  R  T  P  C  Q  T  L  L  S  S  L  I  S  Q  T  S  S  R  S  K  N  H  P  T  N  F3
6601 CTGGGTTCAGGACCCCTGCGCCGCTTCGGACTCCTTGCCAGACTCTGTTGTCATCTTTGATTTCACAGACCTCTTCCCGCTCCAAGAACCATCCCACGAA 6700
     ----:----|----:----|----:----|----:----|----:----|----:----|----:----|----:----|----:----|----:----|
```

```
      S  L  R  L  V  L  G  V  P  K  G  R  C  L  C  G  *  E  R  L  R  P  P  A  V  S  E  S  I  E  S  S  V     F1
     P  P  S  V  L  F  S  E  Y  Q  K  G  D  V  S  A  D  E  N  D  S  G  P  Q  Q  F  L  S  L  *  S  H  L  S   F2
      L  P  P  S  C  S  R  S  T  K  R  E  M  S  L  R  M  R  T  T  P  A  P  S  S  F  *  V  Y  R  V  I  C    F3
6701 CCTCCCTCCGTCTTGTTCTCGGAGTACCAAAAGGGAGATGTCTCTGCGGATGAGAACGACTCCGGCCCCCAGCAGTTTCTGAGTCTATAGAGTCATCTGT 6800
     ----:----|----:----|----:----|----:----|----:----|----:----|----:----|----:----|----:----|----:----|

      P  A  K  S  C  K  P  W  P  A  L  G  C  R  N  P  G  G  L  A  N  H  Q  V  M  A  S  H  C  R  E  R  R     F1
       Q  Q  S  P  A  N  L  G  L  H  W  G  A  E  T  L  A  A  W  P  I  T  K  *  W  L  V  T  V  G  R  D  A    F2
     P  S  K  V  L  Q  T  L  A  C  T  G  V  Q  K  P  W  R  L  G  Q  S  P  S  N  G  *  S  L  S  G  E  T  H   F3
6801 CCCAGCAAAGTCCTGCAAACCTTGGCCTGCACTGGGGTGCAGAAACCCTGGCGGCTTGGCCAATCACCAAGTAATGGCTAGTCACTGTCGGGAGAGACGC 6900
     ----:----|----:----|----:----|----:----|----:----|----:----|----:----|----:----|----:----|----:----|

     T  *  V  Y  P  A  C  G  G  G  H  L  C  S  P  R  S  G  P  R  G  S  R  C  S  E  C  S  R  H  F  I  *  G   F1
      P  K  C  I  Q  P  A  E  G  V  I  C  V  R  R  E  A  A  L  G  G  R  A  A  L  S  V  P  A  T  L  F  R    F2
      L  S  V  S  S  L  R  R  G  S  F  V  F  A  E  K  R  P  S  G  V  A  L  L  *  V  F  P  P  L  Y  L  G    F3
6901 ACCTAAGTGTATCCAGCCTGCGGAGGGGGTCATTTGTGTTCGCCGAGAAGCGGCCCTCGGGGGTCGCGCTGCTCTGAGTGTTCCCGCCACTTTATTTAGG 7000
     ----:----|----:----|----:----|----:----|----:----|----:----|----:----|----:----|----:----|----:----|

      T  L  G  Q  F  S  W  F  L  S  Q  P  W  N  C  C  S  R  C  V  L  G  F  G  G  C  H  P  Q  E  L  P  R     F1
     A  H  S  G  S  S  A  G  S  *  A  S  H  G  I  A  A  P  G  V  C  L  A  L  V  G  A  T  R  R  N  F  P  G   F2
      H  T  R  A  V  Q  L  V  P  K  P  A  M  E  L  L  L  P  V  C  A  W  L  W  W  V  P  P  A  G  T  S  Q    F3
7001 GCACACTCGGGCAGTTCAGCTGGTTCCTAAGCCAGCCATGGAATTGCTGCTCCCGGTGTGTGCTTGGCTTTGGTGGGTGCCACCCGCAGGAACTTCCCAG 7100
     ----:----|----:----|----:----|----:----|----:----|----:----|----:----|----:----|----:----|----:----|

      G  H  L  T  G  L  D  R  P  E  L  L  L  I  P  R  D  I  *  D  V  S  L  C  D  V  C  P  S  A  V  W  R     F1
       D  T  *  P  A  S  I  A  Q  S  S  C  *  S  P  G  T  F  R  M  *  A  F  V  M  F  A  H  L  L  S  G  D    F2
     G  T  L  D  R  P  R  S  P  R  A  P  A  D  P  Q  G  H  L  G  C  E  P  L  *  C  L  P  I  C  C  L  A  T   F3
7101 GGGACACTTGACCGGCCTCGATCGCCCAGAGCTCCTGCTGATCCCCAGGGACATTTAGGATGTGAGCCTTTGTGATGTTTGCCCATCTGCTGTCTGGCGA 7200
     ----:----|----:----|----:----|----:----|----:----|----:----|----:----|----:----|----:----|----:----|

     L  L  C  D  F  S  Y  *  F  L  D  E  R  E  T  E  P  Y  S  C  L  L  G  I  R  N  T  C  R  V  P  F  F  I   F1
      F  S  V  I  F  H  T  D  S  L  M  R  E  K  L  S  P  T  H  V  Y  *  A  L  G  I  L  A  E  C  P  F  L    F2
      S  L  *  F  F  I  L  I  P  *  *  E  R  N  *  A  L  L  M  F  I  R  H  *  E  Y  L  Q  S  A  L  F  Y    F3
7201 CTTCTCTGTGATTTTTCATACTGATTCCTTGATGAGAGAGAAACTGAGCCCTACTCATGTTTATTAGGCATTAGGAATACTTGCAGAGTGCCCTTTTTTA 7300
     ----:----|----:----|----:----|----:----|----:----|----:----|----:----|----:----|----:----|----:----|

      F  *  I  F  W  L  I  F  L  N  L  F  E  F  I  I  L  S  L  L  G  I  K  L  *  L  P  L  K  C  C  K  H    F1
     F  F  E  F  F  G  *  F  F  L  I  C  L  N  L  S  F  Y  P  F  W  G  *  N  F  N  Y  H  *  S  V  A  N  I   F2
      F  L  N  F  L  V  N  F  S  *  F  V  *  I  Y  H  F  I  P  F  G  D  K  T  L  I  T  I  K  V  L  Q  T    F3
7301 TTTTTTGAATTTTTTGGTTAATTTTTCTTAATTTGTTTGAATTTATCATTTTATCCCTTTTGGGGATAAAACTTTAATTACCATTAAAGTGTTGCAAACA 7400
     ----:----|----:----|----:----|----:----|----:----|----:----|----:----|----:----|----:----|----:----|

      F  F  S  H  S  C  S  L  E  V  E  F  P  D  F  P  V  Y  F  S  A  S  P  Y  L  L  W  S  T  S  V  F  L     F1
       S  F  P  T  H  V  L  W  K  *  N  F  L  I  F  L  S  I  S  V  P  L  L  I  C  C  G  Q  P  V  F  S  F    F2
```

```
      F  L  F  P  L  M  F  F  G  S  R  I  S  *  F  S  C  L  F  Q  C  L  S  L  S  V  V  V  N  Q  C  F  P  F     F3
7401 TTTCTTTTCCCACTCATGTTCTTTGGAAGTAGAATTTCCTGATTTTCCTGTCTATTTCAGTGCCTCTCCTTATCTGTTGTGGTCAACCAGTGTTTTCCTT 7500
     ----:----|----:----|----:----|----:----|----:----|----:----|----:----|----:----|----:----|----:----|
                                  Cloning primer <----------------------

      F  F  F  G  L  H  Q  W  H  T  D  M  L  *  N  K  M  N  L  L  I  K  T  *  N  N                          F1
       F  F  L  V  S  I  S  G  T  L  I  C  C  K  T  K  *  I  Y  *  *  R  L  K  I  T                         F2
        F  F  W  S  P  S  V  A  H  *  Y  A  V  K  Q  N  E  F  I  N  K  D  L  K  *  P                        F3
7501 TTTTTTTTTGGTCTCCATCAGTGGCACACTGATATGCTGTAAAACAAAATGAATTTATTAATAAAGACTTAAAATAACC                      7600
     ----:----|----:----|----:----|----:----|----:----|----:----|----:----|----:----
```
