# Supplementary material for: Molecular cloning of PRD-like homeobox genes expressed in bovine oocytes and early IVF embryos
Source: BMC Genomics. 2024 Nov 6;25:1048. doi: 10.1186/s12864-024-10969-w (PMC11542365; doi:10.1186/s12864-024-10969-w)
Supplement: Supplementary file 11 — Supplementary Material 11: Additional file 24: Text S1. UCSC genome browser tracks for PRDL TF genes. (.pdf) Additional file 25: Text S2. UCSC genome browser track for visualizing SNVs. [file 12864_2024_10969_MOESM11_ESM.pdf]

Supplementary Text 1. UCSC genome browser tracks for PRDL TF genes.

track name="ARGFX" description="bovine ARGFX cDNA clone and the putative ORF" visibility=2 itemRgb="On" colorByStrand="198,83,55 198,83,55"

chr1 66070409 66087657 ARGFX_16c_01 1000 + 66070474 66087511
0 6 93,68,51,81,149,557, 0,3330,3398,9251,15283,16691,

track name="DUXA" description="bovine DUXA cDNA clones and the putative ORFs" visibility=2 itemRgb="On" colorByStrand="198,83,55 198,83,55"

chr18 64398319 64406694 DUXA_16c_01 1000 - 64399058 64403851
0 6 236,115,146,103,155,19 0,723,1958,2665,5383,8356,

chr18 64398328 64403879 DUXA_16c_02 1000 - 64399058 64403879
0 5 227,115,146,103,178, 0,714,1949,2656,5373,

track name="LEUTX" description="bovine LEUTX cDNA clones and the putative ORFs" visibility=2 itemRgb="On" colorByStrand="198,83,55 198,83,55"

chr18 49330782 49336014 LEUTX_8c_01 1000 + 49330817 49335666
0 4 42,152,493,323, 0,3527,4416,4909,

chr18 49330772 49334896 LEUTX_8c_02 1000 + 49330817 49334667
0 3 52,152,277, 0,3537,3847,

track name="NOBOX" description="bovine NOBOX cDNA clones and the putative ORFs" visibility=2 itemRgb="On" colorByStrand="198,83,55 198,83,55"

chr4 107667138 107674196 NOBOX_MII_02 1000 - 107667390 107674179
0 8 557,302,232,74,110,107,540,54,
0,1468,2240,2695,3543,3972,4464,7004,

chr4 107667138 107674196 NOBOX_MII_01 1000 - 107667390 107674179
0 8 548,302,232,74,110,107,540,54,
0,1468,2240,2695,3543,3972,4464,7004,

chr4 107667138 107674196 NOBOX_MII_03 1000 - 107667390 107674179
0 8 548,302,232,74,110,107,132,54,
0,1468,2240,2695,3543,3972,4464,7004,

track name="TPRX1" description="bovine TPRX1 cDNA clones and the putative ORFs" visibility=2 itemRgb="On" colorByStrand="198,83,55 198,83,55"

chr18 54684554 54686759 TPRX1_8c_06 1000 - 54685159 54686749
0 3 734,113,35, 0,946,2170,

chr18 54684557 54686747 TPRX1_16c_05 1000 - 54685126 54686747
0 3 695,111,23, 0,1601,2167,

chr18 54684552 54686759 TPRX1_16c_04 1000 - 54684914 54686749
0 4 457,115,170,35, 0,946,1547,2172,

chr18 54684552 54686759 TPRX1_16c_03 1000 - 54685215 54686749
0 2 740,35, 0,2172,

chr18 54684557 54686747 TPRX1_16c_02 1000 - 54685215 54686747
0 5 727,150,24,170,23, 0,802,1032,1542,2167,

chr18 54684557 54686747 TPRX1_16c_01 1000 - 54685031 54686747
0 3 1056,170,23, 0,1542,2167,

track name="TPRX2" description="bovine TPRX2 cDNA clones and the putative ORFs" visibility=2 itemRgb="On" colorByStrand="198,83,55 198,83,55"

chr18 54725806 54727918 TPRX2_16c_01 1000 + 54725839 54727473
0 3 58,170,940, 0,516,1172,

chr18 54725806 54727922 TPRX2_16c_02 1000 + 54727559 54727922
0 2 54,395, 0,1721,

chr18 54725806 54727909 TPRX2_16c_03 1000 + 54725839 54727358
0 4 58,170,84,821, 0,516,1172,1282,

chr18 54725806 54727922 TPRX2_16c_04 1000 + 54727559 54727922
0 2 51,404, 0,1712,

track name="TPRX3" description="bovine TPRX3 cDNA clones and the putative ORFs" visibility=2 itemRgb="On" colorByStrand="198,83,55 198,83,55"

chr18 62775050 62777476 TPRX3_8c_01 1000 - 62775727 62777443
0 3 1214,152,37, 0,1652,2389,

chr18 62775020 62777461 TPRX3_8c_02 1000 - 62775727 62776054
0 2 1244,22, 0,2419,

Supplementary Text 2. UCSC genome browser track for visualizing SNVs.

track name="BLAT Results" indelQueryInsert on description="BLAT Results in bigPsl Format" visibility=full type=bigPsl showDiffBasesAllScales=. baseColorUseSequence=lfExtra baseColorDefault=diffBases bigDataUrl=https://zenodo.org/records/10606461/files/myBigPsl.bb?download=1
